# Role of microRNAs in Osteosarcopenic Obesity/Adiposity: A Scoping Review

**DOI:** 10.3390/cells14110802

**Published:** 2025-05-29

**Authors:** Mariantonia Braile, Adriano Braile, Chiara Greggi, Virginia Veronica Visconti, Giuseppe Toro, Maria Consiglia Trotta, Gianluca Conza, Umberto Tarantino

**Affiliations:** 1 Department of Woman, Child and of General and Specialized Surgery, Università Degli Studi Della Campania “Luigi Vanvitelli”, 81031 Napoli, Italy; 2 Unit of Orthopaedics and Traumatology, Ospedale del Mare, 80147 Naples, Italy; 3 Department of Medical and Surgical Specialties and Dentistry, University of Campania “Luigi Vanvitelli”, 80138 Naples, Italy; 4 Department of Clinical Sciences and Translational Medicine, University of Rome “Tor Vergata”, via Montpellier 1, 00133 Rome, Italyumberto.tarantino@uniroma2.it (U.T.); 5 Department of Biomedicine and Prevention, University of Rome “Tor Vergata”, via Montpellier 1, 00133 Rome, Italy; 6 Department of Experimental Medicine, University of Campania “Luigi Vanvitelli”, 80138 Naples, Italy; 7 Faculty of Medicine and Surgery, University “Our Lady of Good Counsel”, Rruga Dritan Hoxha, 1000 Tirana, Albania

**Keywords:** microRNAs (miRNAs), osteosarcopenic adiposity (OSA), osteosarcopenic obesity (OSO), osteosarcopenia

## Abstract

**Background:** Osteosarcopenic obesity (OSO) syndrome, also defined as osteosarcopenic adiposity (OSA), is characterized by the concurrent loss of bone and muscle mass, accompanied by excess fat, leading to reduced functionality and metabolic imbalances. Recent studies have highlighted the role of microRNAs (miRNAs) in the pathophysiology of OSO/OSA, showing differential expression in individuals with osteosarcopenia and obesity. However, a thorough investigation in this area has been limited. **Methods**: A comprehensive search of international bibliographic databases, including Embase, PubMed and Scopus, was conducted. **Results**: From an initial search yielding 1311 records, 19 studies met the eligibility criteria for final evaluation. These findings highlight how physical exercise and nutritional factors can influence miRNA expression, emphasizing their role in promoting better health outcomes in aging populations. Furthermore, the critical role of miRNAs as indicators of muscle atrophy and the biological processes associated with aging and sarcopenia have been documented in various animal studies. **Conclusions**: Despite the limitations of this review, the findings indicate that miRNAs could serve as promising biomarkers and therapeutic targets for managing OSO/OSA. These results suggest that targeted interventions, such as resistance training and lifelong exercise, may effectively influence miRNA expression, potentially alleviating the impacts of OSO/OSA.

## 1. Introduction

Osteosarcopenic obesity (OSO) was first defined in 2014 to describe the intricate cellular communication between fat, bone and muscle [1], leading to a syndrome characterized by the co-occurrence of overweight/obesity, osteopenia/osteoporosis and sarcopenia [2,3]. Recent developments have also led to its definition as osteosarcopenic adiposity (OSA), highlighting the complex interplay between adipose tissue and the musculoskeletal system in this condition [4].

Overweight/obesity, osteopenia/osteoporosis and sarcopenia develop concurrently, contributing to the complex pathophysiology of OSO/OSA, which poses significant health risks, particularly in aging populations [5]. Advances in diagnostic techniques have allowed for a more precise identification of OSO/OSA, highlighting the importance of recognizing this syndrome as a public health concern as the global population ages. Chronic inflammation, insulin resistance and altered cellular metabolism are key factors associated with the syndrome, making it increasingly prevalent, as age-related changes promote fat redistribution in muscle and bone [6,7,8,9]. Given this background, the urgency of identifying biomarkers for the early detection of OSO/OSA has risen, enabling healthcare providers to implement targeted interventions that can reduce the related morbidity and healthcare costs.

Recent studies have shown that microRNAs (miRNAs), which are non-coding RNAs that regulate different gene pathways, may play a critical role in the development and progression of OSO/OSA, negatively affecting muscle fiber quantity and quality [10]. In fact, specific miRNAs, known as myomiRNAs, are critical regulators for both pro-inflammatory cytokines and skeletal muscle function. The most studied are miR-1, miR-133, miR-206, miR-208, miR-486, miR431 and miR-499, which modulate myogenic factors such as serum response factor (SRF), myocyte enhancer factor-2 (MEF2) and myostatin [11]. The influence of obesity on miRNA profiles in the elderly remains poorly understood [12,13]. In general, age-related and obesity-related alterations in silent information regulator sirtuin 1 (SIRT1) expression, a member of a class III histone deacetylase family proteins (HDACs) dependent on nicotinamide adenine dinucleotide (NAD+), have been linked to changes in miRNA expression [14,15]. For instance, there are metabolic consequences to miR-34a’s control of SIRT1 expression; in the liver, activated Farnesoid X Receptor (FXR) stimulates the transcription of Small Heterodimer Partner (SHP), which suppresses p53 transcriptional activity [16]. As a result, miR-34a levels fall and SIRT1 expression is positively regulated [17]. SIRT1 deacetylates and stimulates FXR transcriptional activity, which in turn activates its own expression, resulting in a positive feedback loop of the FXR/miR-34a/SIRT1 pathway [18,19]. miR-34a is increased in nonalcoholic fatty livers in humans [20] and the fatty livers of diet-induced obese mice [21]. The liver of elderly rats showed increased expression of miR-34a and miR-93, which are both involved in SIRT1’s decreased expression [22]. Still, more investigations are needed to fully examine this issue.

Adipose-derived miRNAs have been identified in rodent models as potent mediators of metabolic functions, capable of impacting muscle, liver and immune cell health, underscoring their potential involvement in OSO/OSA etiology [23,24,25,26].

Importantly, regular physical activity emerges as a vital intervention for combating the components of OSO/OSA. Exercise not only improves muscle mass, enhances bone density and reduces excess body fat, but it also mitigates inflammation, improves insulin sensitivity and modulates miRNA expression. In fact, some miRNAs appear to be involved in the response to different types of exercise, thus playing a relevant role in the modulation of myofiber gene expression in the muscle tissue of adults and elderly people. The literature data have shown that after 3 h of one acute exercise, miR-1, miR-133a, miR-133-b and miR181a expression levels were increased [27,28,29], whereas miR-9, miR23a, miR-23b and miR-31 were decreased [30,31]. In addition, Spakova and colleagues also identified miRNA206 as a predictor of response to strength training. High levels of miRNA206 and the resulting improved functional adaptation may be explained by the increased proliferative activity of satellite cells, through the TGF-β/SMAD2-3/MyoD signaling pathway [32]. For instance, it has demonstrated that a resistance training program significantly impacted miRNA expression and osteoporosis markers in elderly women with OSO/OSA [33]. Likewise, low-intensity, lifelong exercise routines could alter miRNA expression and prevent OSO/OSA by mitigating inflammatory processes [34]. These findings highlight the importance of exercise as a strategic approach to alter the disease trajectory of OSO/OSA, emphasizing its beneficial role in addressing the multifaceted aspects of this syndrome.

As society increasingly recognizes the therapeutic potential of miRNAs [35]—previously explored as therapeutic alternatives in conditions such as cancer and cardiovascular diseases—this review seeks to provide an overview of the literature surrounding the role of miRNAs in the etiology of OSO/OSA. By advancing our understanding of these mechanisms, we hope to contribute to the development of effective interventions that leverage the benefits of physical activity and miRNA modulation in addressing this challenging syndrome.

## 2. Materials and Methods

### 2.1. Information Sources and Search Strategy

A scoping review was performed to identify all studies reporting outcomes of miRNA in OSO/OSA. This study was registered on the PROSPERO database [36], number CRD42025642098. Three different databases were searched for the studies: Embase, Pubmed and Scopus. The following keywords were mixed in different combinations for each data bank: “microRNA” or “miRNA” and “Osteosarcopenic Obesity” or “Osteosarcopenic Adiposity” or “Osteosarcopenia”. Boolean operators “AND” and “OR” were employed in our search plan. The research in databases ended on 22 March 2025. To assess the eligibility of each article, each PICO (Population, Intervention, Comparison, Outcome) [37] element was identified as follows: Population (P): human and animal subjects; Intervention (I): miRNA measurement; Comparison (C): case–control; Outcome (O): role of miRNAs. The various topics analyzed in this scoping review are summarized in a checklist according to the Preferred Reporting Items for Systematic reviews and Meta-Analyses extension for Scoping Reviews (PRISMA-ScR) [38,39]. The PRISMA-ScR checklist is reported in Supplementary File S1.

### 2.2. Eligibility Criteria

One author searched the combination of keywords using the Boolean operators AND and OR across the three different databases. Afterwards, the author combined the three spreadsheets into a single spreadsheet, eliminating duplicates (Supplementary File S1). Then, independently, three authors screened each remaining article based on the following inclusion criteria: (i) English language; (ii) studies that evaluate the role of miRNA in OSO/OSA.

Articles were considered to be not eligible considering the following specific exclusion criteria: (i) articles missing one or more keywords; (ii) conference abstract, paper or meeting; Editorial, letter, note or short survey; (iii) articles irrelevant to the main subject; (iv) no English language; (v) retracted; or (vi) review, systematic review or meta-analysis.

The reason for the exclusion of each record is reported in Supplementary File S2. The title and abstract of all studies found in the search were independently examined by two reviewers, who applied the eligibility criteria. In the case of disagreement between the reviewers, a third reviewer was consulted.

### 2.3. Data Extraction and Quality Process

Following selection, data extraction was carried out by manual curation. The data were extracted by 3 authors, who then independently summarized each article’s findings.

## 3. Results

### 3.1. Literature Research

**Figure 1:**
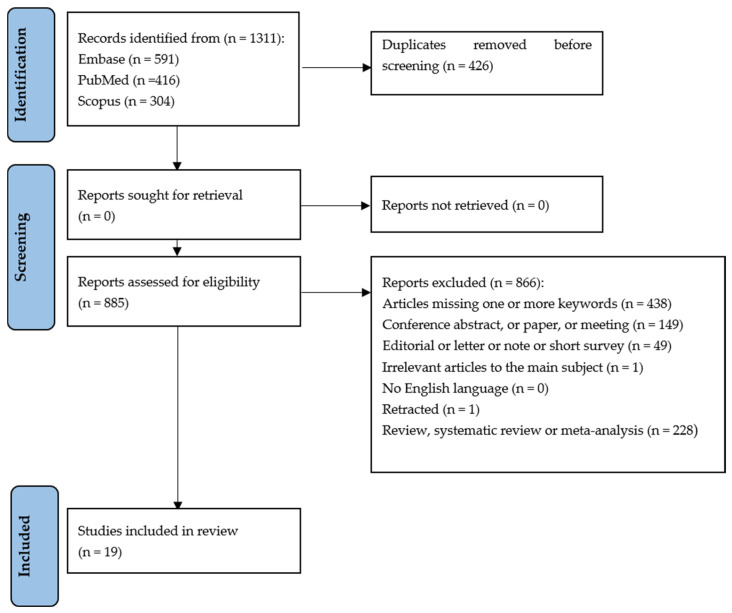
PRISMA-ScR flow diagram showing research strategy [38,39].

The flowchart provides a clear illustration of the process for literature searching (Figure 1). A total of 1311 articles were obtained by searching Pubmed, Embase and Scopus based on the following keywords: “microRNA” or “miRNA” and “Ostosarcopenic Obesity”.

After discarding duplicates, 885 articles were assessed according to the eligibility criteria. After completing the screening procedure, 19 articles were included in this scoping review in order to elucidate the role of miRNA in OSO/OSA.

### 3.2. Study Characteristics

The main characteristics of the 19 included studies are reported in Table 1. The included articles were published between 2010 and 2024. Eight articles were conducted on humans, seven on animal models and four on both. The range of participants included was from 21 to 534, with a total of 1011 participants. Of these, 302 were women and 302 were men. The sex of the remaining individuals was not specified. Regarding the animal model, the range varied from 12 to 80 animals included, with a total of 255 animals. Of these, 143 were female and 62 were male. A total of 128 mice and 173 rats were also used. As for the methodologies, 13 articles used only PCR methods (qPCR, ddPCR, RT-PCR), 4 used the array and 7 articles employed sequencing methodology. Finally, these studies were conducted on 13 samples from peripheral blood (blood, serum and plasma) and 6 on tissue. Based on the outcomes of each study, we analyzed the role of miRNAs in OSO/OSA by distinguishing them into results obtained on human subjects and animal subjects.

### 3.3. Clinical Outcome

#### 3.3.1. Human Studies

The articles by Banitalebi E et al. [33] and Huang L et al. [40] investigate the expression of microRNAs (miRNAs) following physical exercise (Table 2). The study by Banitalebi E et al. [33] focuses on miR-133 and miR-206 in Iranian women with osteopenia/osteoporosis and examines their correlations with various health indicators after 12 weeks of resistance training using elastic bands. The results showed significant correlations between miR-133 levels and FRAX score (r = −0.845, *p* < 0.001), vitamin D (r = −0.551, *p* = 0.025) and ALP (r = 0.620, *p* = 0.012). Similarly, miR-206 showed analogous correlations with FRAX score (r = −0.847, *p* < 0.001), vitamin D (r = −0.500, *p* = 0.041) and ALP (r = 0.662, *p* = 0.007). Meanwhile, the study by Huang et al. [40] examines the expression of miR-126, miR-146a and miR-222 in community-dwelling older adults after an 8-week cycling training program. They found that exercise increased the expression of these miRNAs for up to 16 weeks post-training, suggesting their involvement in improvements in body composition and cardiorespiratory fitness. Additionally, miR-21 was suggested as a potential mediator of the effects of exercise on strength and muscle health. Both studies highlight the potential role of exercise in modulating miRNA expression and its relevance to health outcomes.

Similar to the study conducted by Banitalebi E et al. [33], the research by Faraldi M et al. [41] also focuses on post-menopausal women, specifically aiming to identify circulating miRNAs as potential biomarkers for muscle mass wasting in this population (Table 2). The cohort was divided into tertiles based on appendicular skeletal muscle mass index (ASMMI) to better highlight the differences in skeletal muscle mass: the first tertile comprised nine participants with an ASMMI of 4.88 ± 0.40 kg·m^−2^; the second tertile included ten participants with an ASMMI of 5.73 ± 0.23 kg·m^−2^; and the third tertile had nine participants with an ASMMI of 6.40 ± 0.22 kg·m^−2^. The study found that five miRNAs (miR-221-3p, miR-374b-5p, miR-146a-5p, miR-126-5p and miR-425-5p) were downregulated, while two miRNAs (miR-145-5p and miR-25-3p) were upregulated in the first tertile (relatively low ASMMI) compared to the third tertile (relatively high ASMMI).

The two articles by He N et al. [42] and Qaisar R et al. [43] focus on the interaction between miRNAs, sarcopenia and cardiovascular issues, providing important insights into the health of the elderly (Table 2). In the study by He N et al. [42], the relationship between sarcopenia and cardiovascular risk factors (CVRF) in elderly Chinese individuals is investigated. The authors highlight how the presence of CVRF is associated with a higher prevalence of sarcopenia in the elderly population, underscoring the crucial role of factors such as hypertension and dyslipidemia in predisposing individuals to sarcopenia. Furthermore, the results show a significant correlation between the levels of the miRNA miR-29b and appendicular muscle mass, suggesting that this miRNA could serve as a potential biomarker for sarcopenia, which is also useful in assessing cardiovascular risk. Similarly, Qiasar et al. [43] explore the correlation between circulating levels of specific miRNAs and indices of sarcopenia in patients with congestive heart failure (CHF). The results indicate that patients with CHF exhibit reduced muscle strength and physical capacity compared to healthy controls. In addition, miR-133a shows the strongest correlation with handgrip strength, an important indicator of sarcopenia, while other miRNAs such as miR-434-3p demonstrate strong diagnostic potential for CHF. Changes in the levels of these miRNAs are associated with signs of inflammation and oxidative stress, suggesting a significant connection between sarcopenia and cardiovascular complications. Both articles emphasize the importance of understanding miRNA levels as indicators in the diagnosis and management of sarcopenia, particularly in vulnerable populations such as the elderly with cardiovascular conditions.

Iannone F et al. [44] conducted a study focused on the association between myomiRs, specifically miR-133b, and sarcopenia, investigating the importance of nutrition as a mediating factor in this relationship (Table 2). The results revealed that reduced levels of miR-133b were significantly correlated with the presence of sarcopenia. This suggests that poor nutrition could contribute to the lowering of miR-133b, with negative repercussions on muscle mass. The research therefore highlights the importance of nutrition in managing sarcopenia and the need to consider myomiRs as potential biomarkers to monitor and address this health issue, especially in the elderly.

La Rosa F et al. [45] examined whether rehabilitation reduces inflammation in sarcopenic patients and explored the biological factors associated with this effect (Table 2). They found a significant increase in the levels of miR-335-3p, a post-transcriptional regulator of IL-37 production, as a result of the rehabilitation program.

Finally, Millet M et al. [46] aimed to identify a microRNA signature linked to sarcopenia among older adults living in the community from the SarcoPhAge cohort (Table 2). Their findings revealed that in individuals with sarcopenia, the expression levels of serum miRNA-133a-3p and miRNA-200a-3p were decreased. This reduction aligns with the role of these microRNAs in regulating the proliferation and differentiation of muscle cells, suggesting they may play a significant role in the development of sarcopenia.

#### 3.3.2. Animal Studies

All the articles focus on miRNAs as key indicators of muscle atrophy and the biological processes involved in aging and sarcopenia conditions in animal models, suggesting potential therapies and interventions for managing age-related muscle issues.

Gao H et al. [47] and Pedraza-Vázquez G et al. [34] evaluate the expression of miRNAs following physical exercise, highlighting their regulatory roles in skeletal muscle growth and aging (Table 3). In the study by Gao H et al. [47], the authors focused on the effects of lifelong moderate-intensity continuous training (MICT) using a treadmill on age-related changes in muscle miRNA expression profiles as well as on muscle atrophy, apoptosis and mitochondrial dysfunction. They found that adult rats engaged in MICT experienced notable improvements in their muscle miRNA profiles and a reduction in age-related muscle deterioration, suggesting that an increased expression of miR-486 may be linked to the beneficial effects observed. Similarly, the research conducted by Pedraza-Vázquez G et al. [34] involved a comparison between rats subjected to low-intensity running exercise and a sedentary control group. The study revealed significant alterations in miRNA expression in the gastrocnemius muscle across different age ranges. They discovered that several miRNAs were differentially expressed before and after physical activity, indicating that exercise can modulate miRNA levels. This modulation was associated with inflammation and muscle aging, with a clear relationship between miRNA expression profiles and the inflammatory status of the exercised versus sedentary rats.

Hamrick M et al. [48] investigated the changes in miRNA expression linked to muscle mass loss and myofiber size reduction due to aging in mice. Their research revealed that aging is associated with significant alterations in the expression of 57 specific miRNAs within the mouse skeletal muscle, many of which correlate with age-related muscle atrophy. To assess the therapeutic potential of leptin, the researchers administered recombinant leptin to aged mice. The treatment resulted in a noteworthy increase in hindlimb muscle mass and the size of the extensor digitorum longus fibers in these aged subjects. These findings underscore the intricate relationships between miRNA regulation, muscle mass and potential interventions for combating muscle atrophy related to aging (Table 3).

In another study, Jung H et al. [49] pointed out that while circulating miRNA levels change with age, their potential as noninvasive biomarkers for muscle atrophy remains largely uncharted (Table 3). They conducted a comprehensive miRNA-seq analysis in the tibialis anterior muscle and serum of a mouse model designed to mimic disuse-induced atrophy, analogous to the acute atrophy observed after prolonged bed rest. The comparative analysis revealed that miR-455-3p was significantly reduced in both the atrophy model and older mice. Additionally, miR-434-3p levels were lower in the serum and muscle of aged mice compared to younger counterparts. These results suggest that the deregulation of miR-455-3p may play a vital role in the mechanisms underlying muscle atrophy, and miR-434-3p might serve as a potential serum biomarker for muscle aging.

To delve deeper into the genome-wide changes in miRNA and mRNA expressions associated with muscle aging, Kim J et al. [50] performed sequencing on samples from the gastrocnemius muscles of mice at two different ages (6 and 24 months). Their analysis identified 34 differentially expressed miRNAs (15 upregulated and 19 downregulated) with age. Among them, miR-206 and miR-434 showed significant changes that were corroborated by previous studies. Intriguingly, eight miRNAs from a clustered region at the Dlk1-Dio3 locus on chromosome 12 exhibited coordinated downregulation. Furthermore, the research unveiled 16 novel miRNAs. The integrative analysis brought to light that these miRNAs might impact muscle aging through their positive regulation of transcription, metabolic processes and kinase activity. A number of age-related miRNAs identified in this study have also been implicated in human muscular diseases, pointing to the broader relevance of these findings (Table 3).

Additionally, Lee H et al. [51]. confirmed that muscle mass and functional decline, along with mitochondrial dysfunction, occur in the quadriceps of ovariectomized (OVX) mice. Their research highlighted that miR-141-3p is upregulated in OVX models, with perturbed mitochondrial function through the inhibition of Fkbp5 and Fibin. This suggests that targeting miR-141-3p might represent a promising therapeutic strategy for addressing obesogenic sarcopenia (Table 3).

Lastly, the study of Pardo P et al. [52] also established a connection between the aging process and the alterations in miRNA expressions in skeletal muscles, noting the downregulation of miR-434-3p, which they identified as an anti-apoptotic miRNA. As such, miR-434-3p may have therapeutic potential in treating muscle atrophy across various pathophysiological contexts, including sarcopenia (Table 3).

#### 3.3.3. Studies on Both Animals and Humans

In a series of studies assessing the role of specific microRNAs (miRNAs) in muscle health and pathology, several key findings emerged from both human and mouse models. Rivas D et al. [53] conducted a randomized controlled trial and identified six differentially expressed miRNAs (miR-1-3p, miR-19b-3p, miR-92a, miR-126, miR-133a-3p and miR-133b) between gainers and losers in older adults following a 6-month progressive resistance exercise training intervention. Notably, miR-19b-3p was strongly associated with increased fat-free mass and exhibited higher levels in young mice compared to aged counterparts, correlating positively with muscle mass and grip strength. Similarly, Yang S et al. [54]. found elevated serum levels of miR-193b in individuals with type 2 diabetes, which negatively correlated with muscle mass and was linked to muscle loss in both healthy and db/db mice. In a related study, Itokazu M et al. [55] demonstrated that the transplantation of perimuscular adipose tissue to young mice inhibited muscular stem cell proliferation via Let-7d-3p, highlighting the inhibitory role of this miRNA on crucial stem cell transcription factors. Finally, Okamura T et al. [56]’s microarray analysis revealed that let-7e-5p was significantly downregulated in orchiectomized mice, suggesting a critical involvement in muscle degradation associated with androgen depletion, as its suppression led to enhanced muscle-specific protein expression and improved mitochondrial function. Collectively, these findings underscore the complex interplay of miRNAs in muscle mass regulation, atrophy and the physiological adaptations to exercise and other metabolic conditions (Table 4).

## 4. Discussion

The purpose of the present study was to provide a broad and recent overview of which miRNAs are involved in modulating the mechanisms that control skeletal muscle development and maintenance. Since the aging process is accompanied by muscle decay and the concomitant accumulation of adipose tissue, the correlation between the modulation of expression levels of miRNAs involved in the regulation of skeletal muscle status and the onset of OSO/OSA syndrome was also investigated. For this purpose, studies conducted both in the human model, using postmenopausal women and older adult men as study populations, and in the animal model were analyzed. It was first found that there are several miRNAs related to the processes of muscle development, differentiation and regeneration. Indeed, Faraldi and colleagues [41] identified miR-146a as a molecule involved in the regulation of muscle quality and mass; this miRNA, along with others, was indeed found to be differentially expressed in postmenopausal women characterized by high and low ASMMI [41]. In agreement, Millet et al. [46] identified miRNAs 133a-3p and 200a-3p as potentially involved in the modulation of muscle cell proliferation and differentiation processes, as decreased expression of these miRNAs was observed in sarcopenic patients, suggesting their role in the mechanisms of sarcopenia onset [46]. The study by Hamrick and colleagues [48] conducted in a mouse model further confirms these findings, as this study showed that aging is associated with significant alterations in the expression of 57 miRNAs specific to the skeletal muscle in mice, many of which are related to age-related muscle atrophy [48]. The correlation between age-dependent muscle decay and modulation of the expression of certain miRNAs is also highlighted by the study by Kim and colleagues [50], in which genome-wide changes in the expression of miRNAs and mRNAs associated with muscle aging were investigated. In fact, the analysis conducted identified 34 differentially expressed miRNAs in 6- and 24-month-old mice, including miR-206 and miR-434, that might have an impact on muscle aging [50]. miR-434-3p could also represent a potential biomarker of musculoskeletal status, as it was observed to be downregulated in aged mice by Pardo and colleagues, further suggesting a link between the aging process and alterations in miRNA expression in skeletal muscles [52].

To date, miRNAs have emerged as promising early biomarkers for a variety of pathologies due to their stability in biological fluids and crucial roles in gene regulation. For instance, miR-134-5p is associated with acute myocardial infarction [57]. Similarly, miR-125a-3p is recognized for its involvement in cancer and autoimmune diseases [58,59], suggesting its utility in early diagnostics. Other miRNAs, such as miR-122-5p, have been studied for their roles in metabolic disorders and liver health [60]. Moreover, miR-146a-5p plays a role in inflammation, indicating chronic inflammatory states [61]. Some of these diseases are known to be accompanied by concomitant muscle decay. In agreement, Qiasar and colleagues have indeed shown that there is a correlation between the expression of certain miRNAs, such as miR-133a, and muscle atrophy typical of sarcopenia in patients with CHF [43]. In contrast, He et al. identified miR-29b as a potential biomarker for sarcopenia associated with cardiovascular issues, since its expression levels were found to correlate with Appendicular Muscle Mass in a sarcopenic population characterized by high cardiovascular risk [42]. Moreover, Yang et al. found that elevated serum levels of miR-193b in subjects with type II diabetes correlated negatively with muscle mass [54]. Therefore, monitoring the expression levels of these miRNAs could facilitate the early diagnosis of various diseases, thereby improving intervention strategies and health outcomes. This understanding is crucial not only for early detection but also for elucidating the pleiotropic functions of miRNAs in general, as they often regulate multiple target genes involved in diverse biological processes [30]. As research continues, these miRNAs stand out as valuable tools for enhancing our understanding of disease mechanisms and developing effective clinical applications.

Several studies have also strongly demonstrated that the performance of exercise is able to modulate the expression of several miRNAs, thus exerting an effect on muscle mass and muscle response to the physical activity performed. Indeed, the work of Huang et al. highlights that 8-week cycling training was able to modulate the expression of several miRNAs involved in muscle health, and in particular in modulating body composition and cardiorespiratory capacity [40]. In agreement, Gao and colleagues [47] also highlighted the role of exercise (continuous high intensity training) in modulating the expression of miRNAs that have regulatory roles in skeletal muscle development and maintenance. Indeed, they found that adult rats subjected to this type of training showed significant improvements in the expression pattern of muscle miRNAs, and a concomitant reduction in age-related muscle deterioration; specifically, the increased expression of miR-486 may be related to the observed beneficial effects [47]. Therefore, it appears that modulating the expression of miRNAs involved in the regulation of muscle quality status in high-intensity exercise, as also shown in the study by Rivas and colleagues, who identified miRNAs miR-1-3p, miR-19b-3p, miR-92a, miR-126, miR-133a-3p, and miR-133b differentially expressed in groups of older adults who had shown loss or gain of muscle mass after 6 months of progressive resistance training [53].

Unfortunately, to date there are only two studies in the literature analyzing the modulation of muscle miRNA expression levels in response to the performance of exercise in humans and animals characterized by OSO/OSA syndrome. In detail, Banitalebi et al. [33] found that a 12-week elastic band resistance training program significantly improved myomiR (miR-133 and miR-206) expression and markers of osteoporosis in elderly women with OSO/OSA. Both miR-133 and miR-206 have been shown to be members of a family of muscle-specific miRNAs expressed predominantly in skeletal and cardiac muscle tissues, playing a crucial role in muscle development, differentiation and regeneration [62,63,64,65,66]. In fact, these miRNAs target several key genes involved in muscle biology, including Mef2c (a transcription factor crucial for muscle differentiation) [67,68] and Hdac4 (Histone deacetylase that represses muscle gene expression) [67,68], SRF and Cx43 (which participate in skeletal muscle differentiation) [69]. As myomiRs are responsible for intercellular communication, they may also be essential for the one health improvements provided by exercise programs [70], supporting the data in our scoping review. This aligns with the findings of a study by Yang et al. (2023) [71], which also reported that resistance training notably enhanced muscular strength and bone mineral density in older adults, indicating a positive impact on the components of OSO/OSA. Furthermore, these improvements in myomiRs, which are crucial in muscle regeneration and adaptation, suggest potential biomarker utility for assessing the efficacy of exercise interventions, as supported by evidence from Spakova et al. (2020) [32], who emphasized the role of myomiRs in muscle physiology and pathology.

In contrast, Pedraza-Vázquez et al. [34] demonstrated that a low-intensity lifelong exercise routine could modulate miRNA expression and reduce inflammation, ultimately preventing OSO/OSA. Most of the DEmiRs found in rats are involved in both osteogenesis and metabolic syndromes. Among them, the following miRNAs were identified: miR-125a-3p [72,73,74,75,76], miR-134-5p [77,78], miR-23a-5p [78,79,80,81,82], miR-30e-5p [83,84], miR-494-3p [85,86,87,88], miR-122-5p [89,90,91,92], miR-152-3p [93,94] and miR-20a-5p [95,96,97]. Some of these are involved mainly in osteogenesis, such as miR-194-5p [98,99], miR-497-5p [100], miR-127-3p [101] and miR-672-5p [102], while miR-32-3p [103,104] is associated with both functions. In contrast, miR-10b-5p appears to be involved in both osteogenesis [105,106,107,108] and atrophy [109], and miR-146a-5p regulates bone mass and bone remodeling [110,111]. Also identified was miR-3075, specifically associated with obesity [112], and miR-296-3p [113,114]. Finally, unlike the previous ones, miR-678, miR-1839-5p and miR-6332 appear to be unknown in the main mechanisms involved in OSO/OSA, so deeper investigations would be interesting to carry out in this context.

It is interesting that the authors demonstrate how exercise can alter the epigenetic factor, represented by miRNAs, as people age, decreasing muscle atrophy and significantly affecting how the inflammatory component is regulated [115,116]. According to the study, two miRNAs linked to inflammation in the 8–12 month SED and LRER groups are miR-134-5p and miR-23a-5p. miR-134-5p may control inflammation and the loss of muscle tissue in SED rats by inducing apoptosis and inflammation in fibroblasts and epithelial cells [104]. The expression of miR-134-5p may be inhibited by the LRER exercise regimen. However, miR-23a-5p may be used to trigger compensatory processes including myoblast differentiation and proliferation. LRER (12–18 months) has high expressions of four miRNAs (miR-10b-5p, miR-494-3p, miR-127-3p, and miR-122-5p) linked to pro-inflammatory processes [108,117,118,119], indicating their involvement in metabolic disorders linked to the buildup of lipid tissue in response to inflaming or injury. At least three miRNAs shared with the LRER group, such as miR-30e-5p, cause a protective inflammatory response in SED rats 12–18 months of age [120,121,122]. When miRNA regulation is examined in the later stages of life (18–24 months), in the aging process, four miRNAs (miR146a-5p, miR-152-3p, miR-296-3p and miR-20a-5p) are upregulated in LRER and linked to inflammation suppression [123,124,125,126,127,128,129]. When combined, these findings lend more credence to the idea that miRNAs are linked to inflammation, one of the major factors that causes OSO/OSA. The authors demonstrated through the LRER exercise program that lower body fat also lowers inflammation, and that this process is linked to the differentiating expression of different miRNAs as people age. Additionally, the authors affirm that long-term low-intensity exercise is protective to muscle function [130,131]; by contrast, sedentarism is linked to a stronger pro-inflammatory profile in older adults [132]. By allowing adipocytes to infiltrate the muscle tissue, this regulatory mechanism may reduce the inflammatory profile.

Their findings are consistent with research by Fan et al. [11], which highlighted that regular physical activity leads to favorable changes in inflammatory markers and enhances metabolic health in older adults. This synergy between exercise and inflammatory regulation is critical, as chronic inflammation is a known contributor to OSO/OSA and other age-related diseases. Moreover, the focus on low-intensity exercise reflects a growing body of literature emphasizing its benefits for older adults, such as the study by Ultimo et al. [31], which reported significant health improvements with moderate exercise, making it feasible for broader populations.

Both the studies by Banitalebi et al. [33] and Pedraza-Vázquez et al. [34] provide significant insights into the relationship between exercise, miRNA expression and the management of OSO/OSA; however, they face several weaknesses and criticisms. Banitalebi et al.’s [16] randomized controlled trial is limited by its relatively small sample size, which may restrict the generalizability of the findings to broader populations. Additionally, the study focuses exclusively on elderly Iranian women, potentially overlooking the effects of resistance training in other demographics, such as men or younger individuals. The intervention duration of 12 weeks may also be insufficient to ascertain the long-term sustainability of the observed changes in myomiRs and osteoporosis markers, raising questions about the lasting impact of the training regimen.

Conversely, the observational design of Pedraza-Vázquez et al.’s study [34] limits the ability to draw causal inferences regarding the changes in miRNA expression and inflammation resulting from the low-intensity lifelong exercise routine. The study’s reliance on subjective measures of exercise intensity could result in variability in adherence and outcomes, further complicating the interpretation of results. Moreover, while the research identifies important relationships between exercise and various health markers, it does not delve deeply into the underlying mechanisms by which exercise modifies miRNA profiles and inflammation, leaving gaps in understanding. Both studies would benefit from larger, more diverse cohorts and more rigorous methodologies to enhance the robustness of their findings and their applicability to the broader population.

Such improvements could lead to a more comprehensive understanding of the effects of physical activity on miRNA expression and its implications for managing OSO/OSA, ultimately informing the development of effective public health strategies and clinical interventions aimed at improving health outcomes for aging populations.

To our knowledge, this is the first scoping review that specifically discusses the effects of miRNA expression on patients with OSO/OSA. Nevertheless, the number of studies included in this analysis was very limited, with only two articles, which may have had some influence on the reliability of the outcomes, similar to that found by the group of Yang et al. [133] in discussing the effects of exercise in patients with OSO/OSA. Despite this, both the existing literature, as previously described, and a clear and well-defined methodological protocol (PROSPERO) [36] solidly support the data obtained by the two research groups, lending significant scientific relevance to our scoping review although the discussion is limited to these two papers.

## 5. Conclusions

In conclusion, the emerging relationship between exercise, miRNA expression and osteoporosis underscores the potential of physical activity as a pivotal intervention for combating OSO/OSA. Continued research in this domain is essential for unraveling the biological mechanisms involved and for translating these findings into effective preventative and therapeutic strategies.

## Figures and Tables

**Table 1:** Key features of the studies included in our scoping review.

Authors	Study Population	Number of Participants	Experimental Subdivision	Time of Measurement	Sample	miRNA Detection Technique	Effect
Banitalebi E et al. (2021) [33]	Human	63 postmenopausal women	Divided into resistance training/control group	Before and 48 h after training	Serum	RT-PCR	After exercise, different correlations were found between miRNAs and Bone Health Parameter.
Huang L et al. (2024) [40]	Human	Not specified	Randomized into exercise/control groups	3 time points: Baseline, Week 8, Week 24	Plasma	qPCR	Exercise improved body fat and cardiorespiratory fitness; specific miRNAs increased after training.
Faraldi M et al. (2024) [41]	Human	28 postmenopausal women	Divided into tertiles based on ASMMI	At enrollment	Plasma	qPCR	ROC curves indicated that discovered miRNAs had excellent diagnostic potential.
He N et al. (2022) [42]	Human	186 participants (59 female, 34 male)	Divided into sarcopenia/ non-sarcopenia groups	November 2016 to March 2017	Plasma	RT-PCR	miR-29b downregulated in sarcopenia group; potential biomarker for sarcopenia, useful in assessing cardiovascular risks in the elderly.
Qaisar R et al. (2021) [43]	Human	181 male participants	Divided into CHF/controls	January 2019 to September 2019	Plasma	RT-PCR	CHF patients showed lower physical capacity; specific miRNAs linked to muscle health and inflammation.
Iannone F et al. (2020) [44]	Human	218 participants (139 females, 79 males)	Divided into sarcopenia/ non-sarcopenia groups	Specific time points not detailed	Plasma	RT-PCR	Lower miR-133b levels associated with sarcopenia; nutrients mediating effect observed.
La Rosa F et al. (2021) [45]	Human	21 elderly patients (13 females, 8 males)	All participants received a rehabilitative treatment program and underwent the same intervention	Baseline (T0) and post-rehabilitation (T1)	Plasma	ddPCR	Improved physical and cognitive parameters; highlighted importance of comprehensive geriatric assessment.
Millet M et al. (2024) [46]	Human	534 older Belgian individuals	Divided into sarcopenia/ non-sarcopenia groups	January 2019 to September 2019	Serum	NGS RT-PCR	Higher malnourished or at-risk individuals in sarcopenic group; miR-133b and miR-206 levels associated with sarcopenia.
Gao H et al. (2021) [47]	Rats	48 female Sprague Dawley rats	Divided into four groups based on training/sedentary conditions	After specified training periods	Skeletal muscles	qRT-PCR	Lifelong MICT improved age-related miR-486 expression; upregulation of mitochondrial activity.
Pedraza-Vázquez G et al. (2023) [34]	Rats	80 female Wistar rats	Divided into treadmill LRER/sedentary control	At 8, 12, 18, 24 months	Gastrocnemius muscle	miRNA array	DEmiRs associated with inflammatory profiles identified across age groups.
Hamrick M et al. (2010) [48]	Mice	48 C57BL/6 mice	Divided by age and leptin treatment	After 10-day treatment period	Serum, Quadriceps muscles	miRNA arrays	Aging altered 57 miRNAs; leptin treatment increased muscle mass and altered miRNAs associated with muscle’s repair.
Jung H et al. (2017) [49]	Mice	15 C57BL/6 mice	Divided into young/aged groups	After designated experimental conditions	Serum, Tibialis anterior muscle	miRNA sequencing qRT-PCR	Findings suggest a significant link between adipose tissue-derived miRNAs and aging-associated muscle atrophy, contributing to our understanding of sarcopenia.
Kim J et al. (2014) [50]	Mice	12 C57BL/6 mice	Divided into young/aged groups	After designated experimental conditions	Gastrocnemius muscles	miRNA sequencing qRT-PCR	Gene expression analysis provided insights into the molecular changes associated with aging and muscle decline, emphasizing the role of miRNAs and their interactions with mRNAs in the context of sarcopenia and muscle atrophy.
Lee H et al. (2021) [51]	Mice	Not specified female C57/BL6 mice	Divided into ovariectomized/sham models	After designated experimental conditions	Gastrocnemius muscle	qRT-PCR miRNA sequencing	Identified miRNA interactions in obesogenic sarcopenia
Pardo P et al. (2017) [52]	Mice	44 C57BL/6J mice	Divided into young/aged	15 weeks post-surgery	Skeletal muscles	Microarray analysis qRT-PCR	miR-434-3p downregulated in aging muscle; it is considered to be an anti-apoptotic miRNA with potential therapeutic applications for addressing muscle atrophy, particularly in the context of sarcopenia and other pathophysiological conditions.
Rivas D et al. (2021) [53]	Human and Mice	73 community-dwelling older adults (43 females, 30 males)	Categorized into losers (those who lost muscle mass)/ gainers (those who gained muscle mass)	January 2019–September 2019 with assessments carried out before and at the end of a 6-month rehabilitation protocol	Plasma	qRT-PCR	Six miRNAs were discovered in humans, while miR-19b-3p was identified as significant in murine models as well, with a particular focus on the comparison between young and older mice. This suggested that it is significantly associated with an increase in lean mass.
		12 male C57BL/6 mice	Mice divided into young/aged groups	After designated experimental conditions	Plasma		
Yang S et al. (2022) [54]	Human and Mice	40 individuals (17 females, 23 males)	Categorized into individuals with type 2 diabetes/ healthy	Assessment was carried out after an overnight fast of at least 10 h	TA and gastrocnemius muscle	RT-PCR, miRNA analysis	The findings suggest that miR-193b plays a critical role in muscle mass regulation and could be a potential therapeutic target for muscle loss associated with type 2 diabetes.
		20 mice: 10 mouse models of type 2 diabetes (BKS.C g–m +/+ Leprdb/J 10 C57BLKS/J wild-type	Categorized into HFD/ control diet	After a 16-week treatment, all mice were euthanized following a 16 h fasting period, after which blood and muscle samples were taken for further analysis	Serum		
Itokazu M et al. (2022) [55]	Human and Mice	18 human participants (10 females, 8 males) divided by age	Humans divided by age	After surgical procedures	Muscle	miRNA array qPCR	Changes in the microRNA profile were linked to the interaction of aged adipocytes, indicating that adipose-derived miRNAs could have an important role in the development of sarcopenia.
		C57BL/6 mice	Mice divided by age	After surgical procedures	Adipose tissue		
Okamura T et al. (2021) [56]	Human and Mice	32 male patients from KAMOGAWA-DM cohort	Humans were divided into two groups based on muscle mass: those with decreased muscle mass/those without	Assessments during August 2015–September 2017	Tissue	miRNA arrays RT-PCR	Investigated association of serum miRNAs with sarcopenia; highlighted roles of specific miRNAs in muscle health.
		12 male C57BL/6 Mice	Mice were divided into two age groups: young (6 months)/ aged (24 months)	Measurements related to muscle dissection were performed after the mice were aged accordingly, with treatments applied following baseline assessments	The soleus muscle		

**Abbreviation:** CHF, congestive heart failure; ddPCR, droplet digital polymerase chain reaction; DEmiRs, differential expression microRNAs; LRER, low-intensity running exercise routine; miRNA, microRNA; NGS, next-generation sequencing; qPCR, quantitative polymerase chain reaction; qRT-PCR, quantitative reverse transcription polymerase chain reaction; ROC, receiver operating characteristic curves; RT-PCR, reverse transcription polymerase chain reaction; T0, baseline time point; T1, post-rehabilitation time point.

**Table 2:** Key features of the human studies included in our scoping review.

Authors	Study Design	Age	Experimental Division	Type	miRNA	Effect
Banitalebi E et al. (2021) [33]	Randomized Controlled	Over 65 years	Divided into Exercise Group: 32 subjects underwent EBRT. Control Group: 31 subjects represented the sedentary control group.	Circulating	miR-133 miR-206	Both miRNAs showed a negative correlation with the FRAX score and vitamin D levels, indicating unfavorable effects on bone health; a positive correlation with ALP levels suggests an increase in bone turnover.
Huang L et al. (2024) [40]	Assessor-Blinded, Parallel, Randomized Controlled	Over 60 years	Divided into Exercise Group: Received supervised cycling training. Control Group: No exercise intervention.	Circulating	miR-126 miR-146a miR-222 miR-21	Exercise improved body fat and cardiorespiratory fitness; increased expression of specified miRNAs post-training. Potential mediating effect of miR-21 on body composition, cardiorespiratory fitness, and lower limb strength, but no significant indirect effect.
Faraldi M et al. (2024) [41]	Cohort	Over 60 years	Divided into tertiles based on the ASMMI: First tertile: 9 participants (ASMMI = 4.88 ± 0.40 kg·m^−2^). Second tertile: 10 participants (ASMMI = 5.73 ± 0.23 kg·m^−2^). Third tertile: 9 participants (ASMMI = 6.40 ± 0.22 kg·m^−2^).	Circulating	miR-221-3p miR-374b-5p miR-146a-5p miR-126-5p miR-425-5p miR-145-5p miR-25-3p	miR-221-3p, miR-374b-5p, miR-146a-5p, miR-126-5p and miR-425-5p were downregulated, while miR-145-5p and miR-25-3p were upregulated in the first tertile.
He N et al. (2022) [42]	Paired Case–Control	Over 65 years	Divided into Sarcopenia group: 93 individuals. Non-sarcopenia group: 93 individuals.	Circulating	miR-29b	miR-29b levels were significantly reduced in elderly patients with sarcopenia and CVRF. Additionally, a strong correlation was found between miR-29b and appendicular skeletal muscle mass (ASM) relative to height squared.
Qaisar R et al. (2021) [43]	Case–Control	Over 65 years	Divided into Patients with CHF: 89 participants. Healthy Controls: 92 participants.	Circulating	miR-21 miR-181a miR-133a miR-434-3p miR-455-3p	CHF patients have elevated levels of miR-21 and reduced levels of miR-181a, miR-133a, miR-434-3p and miR-455-3p compared to healthy controls. HGS showed the strongest correlation with miR-133a.
Iannone F et al. (2020) [44]	Case–Control	Over 65 years	Divided into Sarcopenia Group: 109 participants. Non-Sarcopenic Group: 109 participants.	Circulating	miR-133b	Lower levels of miR-133b were significantly associated with the presence of sarcopenia (*p* = 0.006), though this relationship was influenced by nutritional status, indicating a mediating effect of nutrition on the connection between miR-133b and sarcopenia.
La Rosa F et al. (2021) [45]	Cohort	Over 65 years	The study did not specify comparison groups, as all participants underwent the same rehabilitative treatment and had severe sarcopenia.	Circulating	miR-335-3p miR-657	Upregulation of both miRNAs was observed in severe sarcopenia as a result of the rehabilitation program.
Millet M et al. (2024) [46]	Case–Control	Over 65 years	Divided into Sarcopenic Group: 18 individuals in the screening phase and 92 in the validation phase. Non-Sarcopenic Group: 19 healthy individuals in the screening phase and 92 matched for the validation phase.	Circulating	miR-133a-3p miR-200a-3p miR-744-5p miR-151a-3p	miR-133a-3p, miR-200a-3p and miR-744-5p were downregulated, while miR-151a-3p was upregulated in sarcopenic patients.

**Abbreviation:** ALP, alkaline phosphatase; ASM, appendicular skeletal muscle; ASMMI, appendicular skeletal muscle mass index; CHF, congestive heart failure; CVRF, cardiovascular risk factors; EBRT, elastic band resistance training; FRAX^®^, fracture risk assessment tool; HGS, handgrip strength; miR, microRNA.

**Table 3:** Key features of the animal studies included in our scoping review.

Authors	Study Design	Age	Experimental Division	Sample	miRNA	Effect
Gao H et al. (2021) [47]	Experimental	Starting from 8 months old	Rats underwent treadmill training and were divided into Group 1: Adult-MICT (12 rats)—18 months of moderate-intensity continuous training (MICT) initiated at 8 months. Group 2: Presarcopenia-MICT (12 rats)—8 months of MICT initiated at 18 months. Group 3: Adult-SED (12 rats)—sedentary controls at 8 months. Group 4: Old-SED (12 rats)—aging sedentary controls at 26 months.	Skeletal muscle	miR-486	Age-related loss of miR-486 expression was improved, skeletal muscle atrophy and apoptosis were downregulated and mitochondrial activity and autophagy were upregulated in the adult-MICT group.
Pedraza-Vázquez G et al. (2023) [34]	Experimental	8–12 months 12–18 months 18–24 months	Divided into 33 rats that underwent a treadmill LRER. 47 rats represented the sedentary control group.	Tissue	miR-134-5p miR-678 miR-23a-5p miR-125a-3p miR-6332 miR-3075 miR-30e-5p miR-1839-5p miR-194-5p miR-10b-5p miR-497-5p miR-494-3p miR-127-3p miR-672-5p miR-32-3p miR-122-5p miR-152-3p miR-146a-5p miR-1839-5p (duplicate) miR-296-3p miR-20a-5p	Identification of DEmiRs linked to inflammatory profiles in different experimental age categories.
Hamrick M et al. (2010) [48]	Experimental	12 months and 24 months	24 mice per age group, divided into Age group 1: 12 months (control and leptin-injected groups) Age group 2: 24 months (control and leptin-injected groups).	Quadriceps muscles	miR-685 miR-142-3p miR-206 miR-155	miR-685, miR-142-3p were upregulated, while miR-206 miR-155 were downregulated in leptin treatment compared to control aged mice.
Jung H et al. (2017) [49]	Experimental	6 months and 24 months	Divided into 5 young mice (6 months). 5 old mice (24 months). 5 young mice (6 months) for the induction of disuse muscle atrophy.	Circulating And Tissue	miR-455-3p miR-434-3p	miR-455-3p is involved in muscle atrophy, and an increase in its expression may promote muscle growth. miR-434-3p could be a potential serum biomarker for muscle aging, as it is negatively regulated in the presence of atrophy.
Kim J et al. (2014) [50]	Experimental	6 months and 24 months	Divided into 6 young mice (6 months). 6 old mice (24 months).	Tissue	miR-148a miR-411 miR-136 miR-34a/c miR-92b miR-132 miR-146a miR-152 miR-155 miR-185 miR-203 miR-206 miR-215	miR-148a, miR-411, miR-136 were downregulated, while miR-34a/c, miR-92b, miR-132, miR-146a, miR-152, miR-155, miR-185, miR-203, miR-206, miR-215 were downregulatedin aged mice.
Lee H et al. (2021) [51]	Experimental	Eight-week-old	Divided into Ovariectomized group. Sham-operated group.	Tissue	miR-141-3p	miR-141-3p is upregulated in OVX mice, and this could be a therapeutic target for alleviating obesogenic sarcopenia.
Pardo P et al. (2017) [52]	Experimental	3 months and 26 months	Divided into 20 young group. 2 aged group.	Tissue	miR-434-3p	miR-434-3p as a highly downregulated miRNA in the skeletal muscle of aging mice.

**Abbreviation:** DEmiRs, differentially expressed microRNAs; MICT, moderate-intensity continuous training; OVX, ovariectomized; SED, sedentary; LRER, low-intensity running exercise routine; miRNA, microRNA.

**Table 4:** Key features of the studies involving both humans and animals included in our scoping review.

Authors	Study Design	Age	Experimental Division	Sample	miRNA	Effect
Rivas D et al. (2021) [53]	Randomized Controlled Trial	Over 65 years	All participants underwent PRET and a diet with protein supplementation. They were categorized into 33 losers; 40 gainers.	Circulating	miR-1-3p miR-19b miR-92a miR-126 miR-133a-3p miR-133b	miR-1-3p, miR-19b-3p, miR-92a, miR-126, miR-133a-3p and miR-133b were not identified as differentially expressed between gainers and losers in older adults. The expression of miR-19b-3p was higher in young mice compared to older mice.
	Experimental	3 months and 21 months	Mice were categorized into 6 young mice (3 months); 6 old mice (21 months).	Circulating		
Yang S et al. (2022) [54]	Case–Control	Over 50 years	Categorized into 20 individuals with type 2 diabetes. 20 healthy participants.	Circulating	miR-193b	Increased miR-193b levels: serum levels of miR-193b were found to be elevated in individuals with type 2 diabetes. There is a negative correlation between miR-193b levels and muscle mass in participants with type 2 diabetes, indicating that higher levels of miR-193b are associated with lower muscle mass.
	Experimental	8 ± 0.5 weeks old	Divided into Group 1: Control diet (5 diabetic mice vs. 5 wild type). Group 2: HFD (5 diabetic mice vs. 5 wild type).	Tissues		
Itokazu M et al. (2022) [55]	Translational	mean age = 31.8 years and mean age = 77.5	Divided into young patients. aged patients.	Tissue	miR-8113 miR-6239 miR-7075 let-7d miR-7653 miR-7052 miR-365-2 miR-1940 miR-6906 miR-6898 miR-677 miR-7040 miR-6966 miR-709 miR-7025 miR-1224 miR-7030 miR-6236 miR-490 miR-92a miR-3544 miR-7016 miR-7032 miR-101a miR-3093 miR-30c miR-7076 miR-3104	Reduction in Let-7 miRNA repressor Lin28 A/B and activation of nuclear factor-kappa B signaling can lead to the accumulation of Let-7d-3p in the exosomes of aged PMAT.
	Experimental	5-week-old and 2-year-old	Divided into young mice (5-week-old). old mice (2-year-old).	Tissue		
Okamura T et al. (2021) [56]	Experimental	8-week-old	Divided into ORX Group Sham Group Androgen Treatment Group	Tissue	let-7e-5p	let-7e-5p is reduced in ORX mice and increased after androgen treatment. Finally, serum levels of let-7e-5p were significantly lower in subjects with decreased muscle mass compared to those without decreased muscle mass.

**Abbreviation:** HFD, high-fat diet; miRNA, microRNA; ORX, orchiectomized; PMAT, perimuscular adipose tissue; PRET, progressive resistance exercise training intervention.

## Data Availability

All data generated or analyzed during this study are included in this published article and its Supplementary Files.

## Supplementary Materials

The following supporting information can be downloaded at https://www.mdpi.com/article/10.3390/cells14110802/s1, Supplementary File S1: PRISMA Checklist; Supplementary File S2: List of studies not included and exclusion reasons.

## Abbreviations

The following abbreviations are used in this manuscript:

ALP	Alkaline Phosphatase;
AMSTAR 2	A MeaSurement Tool to Assess systematic Reviews 2
DEmiRs	Differential expression microRNAs
FRAX^®^ score	Fracture Risk Assessment Tool Score
LRER	Low-Intensity Running Exercise Routine
miRNAs	microRNAs
OSA	Osteosarcopenic Adiposity
OSO	Osteosarcopenic Obesity
PRISMA	Preferred Reporting Items for Systematic Reviews and Meta-Analyses
RT-PCR	Reverse transcription polymerase chain reaction
