# Supplementary material for: Role of microRNAs in Osteosarcopenic Obesity/Adiposity: A Scoping Review
Source: Cells. 2025 May 29;14(11):802. doi: 10.3390/cells14110802 (PMC12154469; doi:10.3390/cells14110802)
Supplement: Supplementary file 1 [file cells-14-00802-s001.zip › cells-3652784-supplementary.pdf]

Supplementary File S1. Preferred Reporting Items for Systematic reviews and Meta-Analyses extension for Scoping Reviews (PRISMA-ScR) Checklist

SECTION	ITEM	PRISMA-ScR CHECKLIST ITEM	REPORTED ON PAGE #
**TITLE**			
Title	1	Identify the report as a scoping review.	1
**ABSTRACT**			
Structured summary	2	Provide a structured summary that includes (as applicable): background, objectives, eligibility criteria, sources of evidence, charting methods, results, and conclusions that relate to the review questions and objectives.	1
**INTRODUCTION**			
Rationale	3	Describe the rationale for the review in the context of what is already known. Explain why the review questions/objectives lend themselves to a scoping review approach.	2-3
Objectives	4	Provide an explicit statement of the questions and objectives being addressed with reference to their key elements (e.g., population or participants, concepts, and context) or other relevant key elements used to conceptualize the review questions and/or objectives.	3
**METHODS**			
Protocol and registration	5	Indicate whether a review protocol exists; state if and where it can be accessed (e.g., a Web address); and if available, provide registration information, including the registration number.	3
Eligibility criteria	6	Specify characteristics of the sources of evidence used as eligibility criteria (e.g., years considered, language, and publication status), and provide a rationale.	3
Information sources*	7	Describe all information sources in the search (e.g., databases with dates of coverage and contact with authors to identify additional sources), as well as the date the most recent search was executed.	3
Search	8	Present the full electronic search strategy for at least 1 database, including any limits used, such that it could be repeated.	3
Selection of sources of evidence†	9	State the process for selecting sources of evidence (i.e., screening and eligibility) included in the scoping review.	3
Data charting process‡	10	Describe the methods of charting data from the included sources of evidence (e.g., calibrated forms or forms that have been tested by the team before their use, and whether data charting was done independently or in duplicate) and any processes for obtaining and confirming data from investigators.	3
Data items	11	List and define all variables for which data were sought and any assumptions and simplifications made.	3
Critical appraisal of individual sources of evidence§	12	If done, provide a rationale for conducting a critical appraisal of included sources of evidence; describe the methods used and how this information was used in any data synthesis (if appropriate).	3-4

St. Michael's
Inspired Care.
Inspiring Science.

SECTION	ITEM	PRISMA-ScR CHECKLIST ITEM	REPORTED ON PAGE #
Synthesis of results	13	Describe the methods of handling and summarizing the data that were charted.	4
**RESULTS**			
Selection of sources of evidence	14	Give numbers of sources of evidence screened, assessed for eligibility, and included in the review, with reasons for exclusions at each stage, ideally using a flow diagram.	4
Characteristics of sources of evidence	15	For each source of evidence, present characteristics for which data were charted and provide the citations.	4-11
Critical appraisal within sources of evidence	16	If done, present data on critical appraisal of included sources of evidence (see item 12).	4-11
Results of individual sources of evidence	17	For each included source of evidence, present the relevant data that were charted that relate to the review questions and objectives.	6-9
Synthesis of results	18	Summarize and/or present the charting results as they relate to the review questions and objectives.	6-9
**DISCUSSION**			
Summary of evidence	19	Summarize the main results (including an overview of concepts, themes, and types of evidence available), link to the review questions and objectives, and consider the relevance to key groups.	20-23
Limitations	20	Discuss the limitations of the scoping review process.	23
Conclusions	21	Provide a general interpretation of the results with respect to the review questions and objectives, as well as potential implications and/or next steps.	23
**FUNDING**			
Funding	22	Describe sources of funding for the included sources of evidence, as well as sources of funding for the scoping review. Describe the role of the funders of the scoping review.	23

JBI = Joanna Briggs Institute; PRISMA-ScR = Preferred Reporting Items for Systematic reviews and Meta-Analyses extension for Scoping Reviews.
* Where *sources of evidence* (see second footnote) are compiled from, such as bibliographic databases, social media platforms, and Web sites.
† A more inclusive/heterogeneous term used to account for the different types of evidence or data sources (e.g., quantitative and/or qualitative research, expert opinion, and policy documents) that may be eligible in a scoping review as opposed to only studies. This is not to be confused with *information sources* (see first footnote).
‡ The frameworks by Arksey and O'Malley (6) and Levac and colleagues (7) and the JBI guidance (4, 5) refer to the process of data extraction in a scoping review as data charting.
§ The process of systematically examining research evidence to assess its validity, results, and relevance before using it to inform a decision. This term is used for items 12 and 19 instead of "risk of bias" (which is more applicable to systematic reviews of interventions) to include and acknowledge the various sources of evidence that may be used in a scoping review (e.g., quantitative and/or qualitative research, expert opinion, and policy document).

From: Tricco AC, Lillie E, Zarin W, O'Brien KK, Colquhoun H, Levac D, et al. PRISMA Extension for Scoping Reviews (PRISMAScR): Checklist and Explanation. Ann Intern Med. 2018;169:467–473. doi: 10.7326/M18-0850.

Supplementary File S2. List of studies not included and exclusion reasons.

Author		Title	Year	DOI	Exclusion Criteria
Aare S	et al.	Failed reinnervation in aging skeletal muscle	2016	10.1186/s13395-016-0101-y	One or more keywords are missing
Abdulsalam A	et al.	Diagnosing osteosarcopenia: rethinking the role of computed tomography scans	2025	10.1007/s40618-024-02432-0	Letter
Abdulsalam A	et al.	Osteosarcopenia: Adjust for the BMI or Bare the Bias	2024	10.1007/s00223-024-01203-6	Letter
Abe K	et al.	Osteosarcopenia impacts treatment outcomes for Barcelona Cancer Liver Classification stage A hepatocellular carcinoma	2024	10.1016/j.suronc.2024.102043	One or more keywords are missing
Abe T	et al.	Clinical impact of preoperative osteosarcopenia for patients with pancreatic cancer after curative resection,	2024	10.1016/j.hpb.2024.07.052	Conference Abstract
Abe T	et al.	Prognostic Impact of Preoperative Osteosarcopenia for Patients with Pancreatic Ductal Adenocarcinoma After Curative Resection	2023	10.1245/s10434-023-13936-z	One or more keywords are missing
Abidin NZ	et al.	Total vs. bioavailable: Determining a better 25(OH)D index in association with bone density and muscle mass in postmenopausal women	2021	10.3390/metabo11010023	One or more keywords are missing
Abidin NZ	et al.	Determination of Cutoff Values for the Screening of Osteosarcopenia in Obese Postmenopausal Women	2021	10.1155/2021/6634474	One or more keywords are missing
Abraham L	et al.	Quantifying Sarcopenia and Treatment Effects Using Imaging Biomarkers in a	2024		Conference Abstract

		Preclinical Model of Bone Metastasis			
Ackert-Bicknell C	et al.	Proceedings of the Post-Genome Analysis for Musculoskeletal Biology Workshop	2023	10.1007/s11914-023-00781-y	Review
Aggarwal I	et al.	A Pilot Study To Assess The Prevalence Of Sarcopenia, Sarcopenic Obesity And Osteosarcopenic Obesity In Asian Patients With Endometrial Cancer	2025	10.1016/j.ijgc.2024.100678	Conference Abstract
Aggarwal V	et al.	Prevalence of testosterone deficiency in elderly male and its association with frailty and mobility at a tertiary care centre	2021	10.4103/ijem.ijem_289_21	One or more keywords are missing
Agostini S	et al.	Evaluation of serum miRNAs expression in frail and robust subjects undergoing multicomponent exercise protocol (VIVIFRAIL)	2023	10.1186/s12967-023-03911-3	One or more keywords are missing
Agostini S	et al.	Sarcopenia associates with SNAP-25 SNPs and a miRNAs profile which is modulated by structured rehabilitation treatment	2021	10.1186/s12967-021-02989-x	One or more keywords are missing
Ahmad N	et al.	MicroRNA-672-5p Identified during Weaning Reverses Osteopenia and Sarcopenia in Ovariectomized Mice	2019	10.1016/j.omtn.2019.01.002	One or more keywords are missing
Ahmadinezhad M	et al.	Prevalence of osteosarcopenic obesity and related factors among Iranian older people: Bushehr Elderly Health (BEH) program	2023	10.1007/s11657-023-01340-9	One or more keywords are missing
Akarirmak U	et al.	Risk factors associated with falls in inpatients of a physical medicine and rehabilitation clinic	2019	10.1007/s00198-019-04993-w	Conference Abstract

Al Saedi A	et al.	Fat, as a disturbing factor in Muscle, and Bone crosstalk	2023	10.1002/jbm4.10726	Conference Abstract
Al Saedi A	et al.	Comparative analysis of fat composition in marrow, serum, and muscle from aging C57BL6 mice	2022	10.1016/j.mad.2022.111690	One or more keywords are missing
Al Saedi A	et al.	Lipid Signaling Mediators Regulate Bone-Muscle Crosstalk During Ageing	2022	10.1007/s40520-022-02147-3	Conference Abstract
Al Saedi A	et al.	Association Between Tryptophan Metabolites, Physical Performance, and Frailty in Older Persons	2022	10.1177/11786469211069951	One or more keywords are missing
Al Saedi A	et al.	Association between circulating osteoprogenitor cells and sarcopenia	2021	10.1159/000520488	One or more keywords are missing
Al Saedi A	et al.	Lamin A expression in circulating osteoprogenitors as a potential biomarker for frailty: The Nepean Osteoporosis and Frailty (NOF) Study	2018	10.1016/j.exger.2017.11.015	One or more keywords are missing
Al Saedi A	et al.	A new flow cytometry method to quantify lamina expression in circulating osteoprogenitor (COP) cells: A new biomarker for frailty, osteoporosis and sarcopenia	2018	10.1007/s00198-018-4465-1	Conference Abstract
Al Saedi A	et al.	A non-invasivemethodtoanalyze lamin a expression in circulating osteopro-genitor (cop) cells as a biomarker for musculoskeletal disease	2017	10.1007/s00198-017-3950-2	Conference Abstract
Al Saedi A	et al.	Comparative analysis of fat composition in marrow, serum, and muscle from aging C57BL6 mice	2022	10.1016/j.mad.2022.111690	One or more keywords are missing

Albala C	et al.	Falls predict osteosarcopenia in Chilean older people,	2019	10.1007/s00198-019-04993-w	Conference Abstract
Albala C	et al.	Osteosarcopenia and mortality in older Chileans,	2018	10.1007/s41999-018-0097-4	Conference Abstract
Alfonzo M	et al.	Extracellular Vesicles as Communicators of Senescence in Musculoskeletal Aging	2022	10.1002/jbm4.10686	Review
Ali H	et al.	Non-Pharmacological Approach to Diet and Exercise in Metabolic-Associated Fatty Liver Disease: Bridging the Gap between Research and Clinical Practice	2024	10.3390/jpm14010061	Review
Alizadeh M	et al.	MicroRNAs in disease States	2025	10.1016/j.cca.2025.120187	Review
Alonso N	et al.	Role of Vitamin K in Bone and Muscle Metabolism,	2023	10.1007/s00223-022-00955-3	Review
Altana V	et al.	MicroRNAs and Physical Activity	2015		Review
Ambudkar I	et al.	ROS in Ca2+ signaling and disease-part 2	2016	10.1016/j.ceca.2016.08.001	Editorial
An L	et al.	Potential roles of miRNA-1245a regulatory networks in sarcopenia	2021	10.2147/IJGM.S334501	One or more keywords are missing
Anbarasu K	et al.	To study the prevalence of sarcopenia and it's associated factors among ambulatory community dwelling older subjects with Type 2 Diabetes mellitus	2022		Conference Abstract
Angileri V	et al.	The pain patterns in frail sarcopenic and osteosarcopenic subjects	2018	10.1007/s41999-018-0097-4	Conference Abstract
Anker M	et al.	Novel biomarkers in heart failure and cardio-oncology	2019	10.5603/KP.2019.0051	Editorial
Aoi W	et al.	Roles of Skeletal Muscle-Derived Exosomes in Organ Metabolic and Immunological Communication	2021	10.3389/fendo.2021.697204	Review

Araújo MM	et al.	What Is the Best Method for Diagnosing Osteosarcopenic Adiposity in Women After Long-Term Bariatric Surgery? A Comparison and Validation of Different Criteria	2024	10.3390/nu16223965	One or more keywords are missing
Arcari I	et al.	Letter: Prioritising osteosarcopenia assessment in the ongoing care of patients with cholestatic liver diseases	2024	10.1111/apt.17842	Letter
Arcidiacono G	et al.	Taking care of inpatients with fragility hip fractures: the hip-padua osteosarcopenia (Hip-POS) fracture liaison service model	2025	10.1007/s40618-024-02425-z	One or more keywords are missing
Armandi A	et al.	The Impact of Dysmetabolic Sarcopenia Among Insulin Sensitive Tissues: A Narrative Review	2021	10.3389/fendo.2021.716533	Review
Armstrong W	et al.	Effects of muscle quantity and bone mineral density on injury and outcomes in older adult motor vehicle crash occupants	2022	10.1080/15389588.2022.2124864	One or more keywords are missing
Aryana IGPS	et al.	Denosumab's Therapeutic Effect for Future Osteosarcopenia Therapy : A Systematic Review and Meta-Analysis	2023	10.4235/agmr.22.0139	Retracted
Aryana IGPS	et al.	Importance of Sclerostin as Bone-Muscle Mediator Crosstalk	2022	10.4235/agmr.22.0036	One or more keywords are missing
Asavamongkolkul A	et al.	Prevalence of osteoporosis, sarcopenia, and high falls risk in healthy community-dwelling Thai older adults: a nationwide cross-sectional study	2024	10.1093/jbmrpl/ziad020	One or more keywords are missing
Assaf S	et al.	Unraveling the Evolutionary Diet Mismatch and Its Contribution to the	2024	10.3390/metabo14070379	Review

		Deterioration of Body Composition			
Asuncion C	et al.	Sarcopenia Screening in Very Elderly Patients Belonging to the Spanish Multicenter Study 'Pippas' Admitted to the Guadalajara Hospital (Spain) for Peri-Prosthetic and Peri-Implant Fractures	2023	10.1177/21514593231164064	Conference Abstract
Atkinson S	et al.	A preview of selected articles	2021	10.1002/sctm.21-0103	Note
Atlihan R	et al.	Non-Pharmacological Interventions in Osteosarcopenia: A Systematic Review	2021	10.1007/s12603-020-1537-7	Review
Avey A	et al.	Muscle-tendon cross talk during muscle wasting	2021	10.1152/AJPCELL.00260.2021	One or more keywords are missing
Azuma K	et al.	Functional mechanisms of mitochondrial respiratory chain supercomplex assembly factors and their involvement in muscle quality	2020	10.3390/ijms21093182	Review
Bae GC	et al.	Effect of Osteosarcopenia on Postoperative Functional Outcomes and Subsequent Fracture in Elderly Hip Fracture Patients	2020	10.1177/2151459320940568	One or more keywords are missing
Bahat G	et al.	The Current Landscape of Pharmacotherapies for Sarcopenia	2024	10.1007/s40266-023-01093-7	One or more keywords are missing
Bakinowska E	et al.	Pathogenesis of Sarcopenia in Chronic Kidney Disease—The Role of Inflammation, Metabolic Dysregulation, Gut Dysbiosis, and microRNA	2024	10.3390/ijms25158474	Review
Balaeva M	et al.	Functional and physical status depending on the state of bone and muscle tissue in patients with geriatric syndromes	2019	10.1007/s41999-019-00221-0	Conference Abstract

Ballesteros J	et al.	The Role of the Kynurenine Pathway in the Pathophysiology of Frailty, Sarcopenia, and Osteoporosis	2023	10.3390/nu15143132	Review
Balogun S	et al.	Prospective associations of osteosarcopenia and osteodynapenia with incident fracture and mortality over 10 years in community-dwelling older adults	2019	10.1016/j.archger.2019.01.015	One or more keywords are missing
Bani Hassan E	et al.	Appendicular and mid-thigh lean mass are associated with muscle strength, physical performance, and dynamic balance in older persons at high risk of falls	2022	10.1016/j.gaitpost.2022.01.022	One or more keywords are missing
Bani Hassan E	et al.	Thigh and forearm as potential regions of interest to diagnose osteosarcopenia by dxa	2020	10.1007/s00198-020-05696-3	Conference Abstract
Bani Hassan E	et al.	Hemoglobin Levels are Low in Sarcopenic and Osteosarcopenic Older Persons	2020	10.1007/s00223-020-00706-2	One or more keywords are missing
Bani Hassan E	et al.	Bone Marrow Adipose Tissue Quantification by Imaging	2019	10.1007/s11914-019-00539-5	Review
Bani Hassan E	et al.	Diagnostic Value of Mid-Thigh and Mid-Calf Bone, Muscle, and Fat Mass in Osteosarcopenia: A Pilot Study	2019	10.1007/s00223-019-00582-5	One or more keywords are missing
Baniasadi M	et al.	Role of non-coding RNAs in osteoporosis	2024	10.1016/j.prp.2023.155036	Review
Barberi L	et al.	Circulating Extracellular Vesicles in Alcoholic Liver Disease Affect Skeletal Muscle Homeostasis and Differentiation	2025	10.1002/jcsm.13675	One or more keywords are missing
Barden J	et al.	Regulation of miR-206 in denervated and dystrophic muscles, and	2024	10.1242/jcs.262303	One or more keywords are missing

		its effect on acetylcholine receptor clustering			
Barnsley J	et al.	Pathophysiology and treatment of osteoporosis: challenges for clinical practice in older people	2021	10.1007/s40520-021-01817-y	Review
Bauer JM	et al.	Is There Enough Evidence for Osteosarcopenic Obesity as a Distinct Entity? A Critical Literature Review,	2019	10.1007/s00223-019-00561-w	Review
Bazdyrev E	et al.	Respiratory Muscle Strength in Patients with Coronary Heart Disease and Different Musculoskeletal Disorders	2022	10.20996/1819-6446-2022-08-04	One or more keywords are missing
Bazdyrev E	et al.	Prevalence of musculoskeletal disorders in patients with coronary artery disease	2021	10.20996/1819-6446-2021-06-03	One or more keywords are missing
Bellelli F	et al.	Osteosarcopenia to Raise Awareness on the Complexity of the Older Person	2022	10.14283/jfa.2022.10	Letter
Benoit B	et al.	Treatment with fibroblast growth factor 19 increases skeletal muscle fiber size, ameliorates metabolic perturbations and hepatic inflammation in 5/6 nephrectomized mice	2023	10.1038/s41598-023-31874-4	One or more keywords are missing
Berardi E	et al.	Molecular and cell-based therapies for muscle degenerations: A road under construction	2014	10.3389/fphys.2014.00119	Review
Berg von Linde M	et al.	Insights from the Den: How Hibernating Bears May Help Us Understand and Treat Human Disease	2015	10.1111/cts.12279	One or more keywords are missing
Bermeo S	et al.	The role of the proteins of the nuclear envelope in the pathophysiology of osteosarcopenia,	2012		Conference Abstract
Binkley N	et al.	Dxa use in diagnosis and monitoring of osteosarcopenia,	2017	10.1007/s00198-017-3943-1	Conference Abstract

Biressi S	et al.	The quasi-parallel lives of satellite cells and atrophying muscle	2015	10.3389/fnagi.2015.00140	Review
Blain H	et al.	The benefits of physical activity on successful bone, spine and joint ageing: Highlights of the recent literature,	2022	10.1016/j.jbspin.2022.105434	Editorial
Blomqvist M	et al.	Osteosarcopenia in Finland: prevalence and associated factors	2024	10.1007/s11657-024-01439-7	One or more keywords are missing
Bonanni R	et al.	Osteosarcopenia and Pain: Do We Have a Way Out?	2023	10.3390/biomedicines11051285	One or more keywords are missing
Borja-Gonzalez M	et al.	Inflamma-MiR-21 negatively regulates myogenesis during ageing	2020	10.3390/antiox9040345	One or more keywords are missing
Borja-Gonzalez M	et al.	Aging Science Talks: The role of miR-181a in age-related loss of muscle mass and function	2020	10.1016/j.tma.2020.07.001	Editorial
Bosco F	et al.	Pathophysiological Aspects of Muscle Atrophy and Osteopenia Induced by Chronic Constriction Injury (CCI) of the Sciatic Nerve in Rats	2023	10.3390/ijms24043765	One or more keywords are missing
Bosco F	et al.	The muscle to bone axis (and viceversa): An encrypted language affecting tissues and organs and yet to be codified?	2021	10.1016/j.phrs.2021.105427	Review
Boshnjaku A	et al.	Bone/Muscle Interaction as a Good Biomarker for Lifespan and Quality	2022	10.1177/21514593221111646	Letter
Bottai V	et al.	Bone turnover profile and muscular status in major orthopaedic surgery: a case series	2023	10.23750/abm.v94i3.13880	One or more keywords are missing
Brandi ML	et al.	A perspective on muscle phenotyping in musculoskeletal research	2024	10.1016/j.tem.2024.01.004"	One or more keywords are missing

Brotto M	et al.	Old and new roles of amino acids and amino butyric acids in musculoskeletal diseases	2020	10.1007/s00198-020-05695-4	Conference Abstract
Brown D	et al.	MicroRNAs: Modulators of the underlying pathophysiology of sarcopenia?	2015	10.1016/j.arr.2015.08.007	Review
Bruyere O	et al.	Vitamin D and osteosarcopenia: An update from epidemiological studies	2017	10.1097/MCO.0000000000000411	Review
Bruyere O	et al.	Non-pharmacological therapies for the management of osteosarcopenia	2019	10.1093/rheumatology/kez109.027	Conference Abstract
Brzeszczyńska J	et al.	Role of microRNA in muscle regeneration and diseases related to muscle dysfunction in atrophy, cachexia, osteoporosis, and osteoarthritis	2020	10.1302/2046-3758.911.bjr-2020-0178.r1	Review
Buehring B	et al.	Diagnostic approach to osteosarcopenia	2019	10.1007/s00198-019-04986-9	Conference Abstract
Buratto J	et al.	Safety and Efficacy of Testosterone Therapy on Musculoskeletal Health and Clinical Outcomes in Men: A Systematic Review and Meta-Analysis of Randomized Placebo-Controlled Trials	2023	10.1016/j.eprac.2023.04.013	Review
Burgueno-Aguilar K	et al.	Dysmobility syndrome: a case-series study describing a musculoskeletal syndrome in postmenopausal Mexican women	2021	10.1007/s11657-021-00897-7	One or more keywords are missing
Burton M	et al.	The serum small non-coding RNA (SncRNA) landscape as a molecular biomarker of age associated muscle dysregulation and insulin resistance in older adults	2024	10.1096/fj.202301089RR	One or more keywords are missing
Büyükşireci DE	et al.	The exercise recommendation for	2023	10.4274/tod.galenos.2023.97059	Review

		patients with osteoporosis: Which type of exercise and when?			
Cacciatore S	et al.	Emerging Targets and Treatments for Sarcopenia: A Narrative Review	2024	10.3390/nu16193271	Review
Cai M	et al.	Research progress of osteosarcopenia	2021	10.3969/j.issn.1674-8115.2021.05.020	Review
Call J	et al.	Meeting Summary 3rd International Conference on Musculoskeletal & Neuronal Interactions	2023	10.1002/jbm4.10726	Conference Pa[illegible]
Candow D	et al.	The potential of creatine monohydrate supplementation in the management of osteosarcopenia	2025	10.1097/MCO.0000000000001118	One or more keywords are missing
Candow D	et al.	Current evidence and possible future applications of creatine supplementation for older adults	2021	10.3390/nu13030745	Review
Cannataro R	et al.	Sarcopenia: Etiology, nutritional approaches, and mirnas	2021	10.3390/ijms22189724	Review
Cao M	et al.	Association of calf circumference with osteoporosis and hip fracture in middle-aged and older adults: a secondary analysis	2024	10.1186/s12891-024-08237-9	One or more keywords are missing
Cariati I	et al.	Role of Physical Activity in Bone-Muscle Crosstalk: Biological Aspects and Clinical Implications	2021	10.3390/jfmk6020055	One or more keywords are missing
Carla L	et al.	Prevalence of osteo-sarcopenia and osteo-sarcopenia obesity in healthy ambulatory subjects older than 45 years in France	2018	10.1007/s41999-018-0097-4	Conference Abstract
Casabella A	et al.	Aromatase inhibitor-induced bone loss osteosarcopenia in older patients with breast cancer: effects of the	2024	10.1007/s11739-024-03725-1	One or more keywords are missing

		RANK/RANKL system's inhibitor denosumab vs. bisphosphonates			
Casas-Martinez J	et al.	Redox regulation of UPR signalling and mitochondrial ER contact sites	2024	10.1007/s00018-024-05286-0	Review
Cava E	et al.	Review	2024	10.1038/s41430-024-01513-w	Review
Cedeno-Veloz B	et al.	BENEFIT OF A MULTIFACTORIAL APPROACH WITH TELEREHABILITATION IN OLDER ADULTS AFTER HIP FRACTURE: THE ACTIVE FLS PRAGMATIC RANDOMIZED CLINICAL TRIAL PROTOCOL	2022	10.1007/s40520-022-02147-3	Conference Abstract
Cedeno-Veloz B	et al.	Osteosarcopenia: A narrative review	2019	10.1016/j.regg.2018.09.010	Review
Çelik Eroğlu B	et al.	Evaluation of muscle and bone composition and function in aging women with polycystic ovary syndrome	2024	10.1016/j.maturitas.2024.107982	One or more keywords are missing
Chang S	et al.	miR-320 regulates myogenesis by targeting growth factor receptor-bound protein-2 and ameliorates myotubes atrophy	2022	10.1016/j.biocel.2022.106212	One or more keywords are missing
Chao C	et al.	Extracellular MicroRNAs as Potential Biomarkers for Frail Kidney Phenotype: Progresses and Precautions	2024	10.14336/AD.2023.0818	One or more keywords are missing
Chao C	et al.	Uremic toxins and frailty in patients with chronic kidney disease: A molecular insight	2021	10.3390/ijms22126270	One or more keywords are missing
Chapurlat R	et al.	Osteosarcopenia: Toward a single entity?	2021	10.1016/j.monrhu.2021.03.006	Short Survey
Chaudry O	et al.	Effects of High-Intensity Resistance Training (HIT-RT) on Visceral Adipose Tissue and Abdominal	2022	10.1016/j.bonr.2022.101429	Conference Abstract

		Aortic Calcifications in Men with Osteosarcopenia,			
Chaytow H	et al.	Spinal muscular atrophy: From approved therapies to future therapeutic targets for personalized medicine	2021	10.1016/j.xcrm.2021.100346	Review
Che J	et al.	MiR-1290 promotes myoblast differentiation and protects against myotube atrophy via Akt/p70/FoxO3 pathway regulation	2021	10.1186/s13395-021-00262-9	One or more keywords are missing
Chen B	et al.	Bibliometric Analysis on Research Trend of Accidental Falls in Older Adults by Using Citespace-Focused on Web of Science Core Collection (2010-2020)	2021	10.3390/ijerph18041663	One or more keywords are missing
Chen C	et al.	Sarcopenia, Frailty and Fall Risk-Narrative Review,	2023	10.6890/IJGE.202307_17(3).0001	Review
Chen F	et al.	Inflammation-dependent downregulation of miR-532-3p mediates apoptotic signaling in human sarcopenia through targeting BAK1	2020	10.7150/ijbs.41641	One or more keywords are missing
Chen J	et al.	Integrating transcriptomic and proteomic data for a comprehensive molecular perspective on the association between sarcopenia and osteoporosis	2024	10.1016/j.archger.2024.105486	One or more keywords are missing
Chen J	et al.	Research Progress on the Effect and Mechanism of Exercise Intervention on Sarcopenia Obesity	2024	10.2147/CIA.S473083	Review
Chen J	et al.	SIRT3 as a potential therapeutic target for heart failure	2021	10.1016/j.phrs.2021.105432	Review
Chen L	et al.	Osteosarcopenia: Potential therapeutic interventions,	2019	10.1007/s00198-019-04986-9	Conference Abstract

Chen L	et al.	Aging, Body Composition, and Cognitive Decline: Shared and Unique Characteristics	2023	10.1007/s12603-023-2022-x	Editorial
Chen L	et al.	Crosstalk between bone and muscle for healthy aging	2019	10.33879/AMH.2019.1913	Editorial
Chen S	et al.	Global epidemiological features and impact of osteosarcopenia: A comprehensive meta-analysis and systematic review	2024	10.1002/jcsm.13392	Review
Chen X	et al.	Keratocan Improves Muscle Wasting in Sarcopenia by Promoting Skeletal Muscle Development and Fast-Twitch Fibre Synthesis	2025	10.1002/jcsm.13724	One or more keywords are missing
Chen Y	et al.	Molecular insights into sarcopenia: ferroptosis-related genes as diagnostic and therapeutic targets	2023	10.1080/07391102.2023.2298390	One or more keywords are missing
Chen Z	et al.	Aerobic exercise enhances mitochondrial homeostasis to counteract D-galactose-induced sarcopenia in zebrafish	2023	10.1016/j.exger.2023.112265	One or more keywords are missing
Chen Z	et al.	Bone and muscle specific circulating microRNAs in postmenopausal women based on osteoporosis and sarcopenia status	2019	10.1016/j.bone.2018.11.001	One or more keywords are missing
Chen ZT	et al.	The value of ultrasound measured rectus femoris thickness, cross-sectional area and shear wave velocity in assessment of muscle in postmenopausal women with osteosarcopenia	2025	10.1093/bjr/tqaf012	One or more keywords are missing
Cheng C	et al.	Physiology and metabolism of tissue-engineered skeletal muscle	2014	10.1177/1535370214538589	One or more keywords are missing

Cheng KY	et al.	Identification of Osteosarcopenia by High-Resolution Peripheral Quantitative Computed Tomography	2024	10.3390/jpm14090935	One or more keywords are missing
Chennamadhavuni A	et al.	Risk Factors and Biomarkers for Immune-Related Adverse Events: A Practical Guide to Identifying High-Risk Patients and Rechallenging Immune Checkpoint Inhibitors	2022	10.3389/fimmu.2022.779691	Review
Chew J	et al.	Nutrition mediates the relationship between osteosarcopenia and frailty: A pathway analysis	2020	10.3390/nu12102957	One or more keywords are missing
Chiapparelli E	et al.	The association between lumbar paraspinal muscle functional cross-sectional area on MRI and regional volumetric bone mineral density measured by quantitative computed tomography	2022	10.1007/s00198-022-06430-x	One or more keywords are missing
Chiapparelli E	et al.	The association of spinal lean muscle volume on lumbar spine MRI and regional volumetric bone mineral density measured by quantitative computed tomography	2021	10.1016/j.spinee.2021.05.124	Conference Abstract
China S	et al.	Globular adiponectin reverses osteo-sarcopenia and altered body composition in ovariectomized rats	2017	10.1016/j.bone.2017.08.005	One or more keywords are missing
Cho SW	et al.	Metabolic phenotyping with computed tomography deep learning for metabolic syndrome, osteoporosis and sarcopenia predicts mortality in adults	2024	10.1002/jcsm.13487	One or more keywords are missing
Choi J	et al.	FGF9 is highly expressed in an osteocyte-like â€œmini-boneâ€ cell line and inhibits C2C12	2015	10.1002/jbmr.2763	Conference Abstract

		myogenesis via overexpression of Myostatin			
Choi M	et al.	Dietary calcium, phosphorus, and osteosarcopenic adiposity in Korean adults aged 50 years and older	2021	10.1007/s11657-021-00961-2	One or more keywords are missing
Choi M	et al.	Protein intake and osteosarcopenic adiposity in Korean adults aged 50 years and older	2020	10.1007/s00198-020-05529-3	One or more keywords are missing
Chou YY	et al.	The associations of osteoporosis and possible sarcopenia with disability, nutrition, and cognition in community-dwelling older adults	2023	10.1186/s12877-023-04431-x	One or more keywords are missing
Close G	et al.	The harder you run, the longer (and smoother) the road: exercise, muscle and ageing	2016	10.1007/s10522-016-9649-5	Editorial
Clynes MA	et al.	Osteosarcopenia: Where osteoporosis and sarcopenia collide	2021	10.1093/rheumatology/keaa755	One or more keywords are missing
Connolly M	et al.	miR-424-5p reduces ribosomal RNA and protein synthesis in muscle wasting	2018	10.1002/jcsm.12266	One or more keywords are missing
Contreras M	et al.	Osteosarcopenia prevalence and its association with comorbidity, frailty, and grip strength in patients with hip fracture	2019	10.1007/s41999-019-00221-0	Conference Abstract
Conzade R	et al.	Changes in Nutritional Status and Musculoskeletal Health in a Geriatric Post-Fall Care Plan Setting	2019	10.3390/nu11071551	One or more keywords are missing
Corrêa T	et al.	Diabetes, microRNA, and Nutrition in Geriatrics	2020	10.1007/s13670-020-00336-4	Review
Cosarderelioglu C	et al.	Relation of osteosarcopenia and sarcopenia alone with fragility vertebral fractures in older adults	2019	10.1007/s41999-019-00221-0	Conference Abstract

Coteli S	et al.	Frequency and related factors of osteosarcopenia in elderly outpatients,	2018	10.1007/s00198-018-4465-1	Conference Abstract
Cowan PTLaunico MVKahai P	et al.	Anatomy, Bones	2025		One or more keywords are missing
Craven BC	et al.	Reducing endocrine metabolic disease risk in adults with chronic spinal cord injury: strategic activities conducted by the Ontario-Quebec RIISC team,	2024	10.1080/09638288.2023.2284223	Review
Cvijetić S	et al.	Osteosarcopenic Adiposity and Nutritional Status in Older Nursing Home Residents during the COVID-19 Pandemic	2023	10.3390/nu15010227	One or more keywords are missing
Cvijetić S	et al.	Prevalence of Osteosarcopenic Adiposity in Apparently Healthy Adults and Appraisal of Age, Sex, and Ethnic Differences	2024	10.3390/jpm14080782	One or more keywords are missing
Cvijetić S	et al.	Body composition and nutritional status in nursing home residents during the COVID-19 lockdown: a 15-month follow-up	2024	10.2478/aiht-2024-75-3886	One or more keywords are missing
da Silva TG	et al.	Spectrochemical analysis of blood combined with chemometric techniques for detecting osteosarcopenia	2023	10.1038/s41598-023-36834-6	One or more keywords are missing
Dai H	et al.	Hierarchically Injectable Hydrogel Sequentially Delivers AntagomiR-467a-3p-Loaded and AntagomiR-874-5p-Loaded Satellite-Cell-Targeting Bioengineered Extracellular Vesicles Attenuating Sarcopenia	2023	10.1002/adhm.202203056	One or more keywords are missing
Daly R	et al.	Lifestyle approaches to prevent falls, fractures and frail bones: An update of the evidence	2016	10.1080/13697137.2016.1242242	Conference Abstract

Dao T	et al.	Prevalence of Sarcopenia and its Association with Antirheumatic Drugs in Middle-Aged and Older Adults with Rheumatoid Arthritis: A Systematic Review and Meta-analysis	2021	10.1007/s00223-021-00873-w	Review
Das C	et al.	Sarcopenia and Osteoporosis	2023	10.1007/s43465-023-01022-1	One or more keywords are missing
Davenport A	et al.	Frailty, appendicular lean mass, osteoporosis and osteosarcopenia in peritoneal dialysis patients	2022	10.1007/s40620-022-01390-1	One or more keywords are missing
Dawson-Hughes B	et al.	Effect of a GH Secretagogue, Anamorelin, on Serum Irisin and Inflammation Levels in Osteosarcopenic Adults	2024	10.1210/jendso/bvae028	One or more keywords are missing
De Rui M	et al.	Parkinson's disease and the non-motor symptoms: hyposmia, weight loss, osteosarcopenia	2020	10.1007/s40520-020-01470-x	Review
De Rui M	et al.	Dietary strategies for mitigating osteosarcopenia in older adults: a narrative review	2019	10.1007/s40520-019-01130-9	Review
De Sanctis P	et al.	Non-coding rnas in the transcriptional network that differentiates skeletal muscles of sedentary from long-term endurance-and resistance-trained elderly	2021	10.3390/ijms22041539	One or more keywords are missing
de Sire A	et al.	Role of Dietary Supplements and Probiotics in Modulating Microbiota and Bone Health: The Gut-Bone Axis	2022	10.3390/cells11040743	Review
de Villiers T	et al.	Update on bone health: the International Menopause Society White Paper 2021	2021	10.1080/13697137.2021.1950967	Review

Debruin D	et al.	Exploring new balance and gait factors that are associated with osteosarcopenia in patients with a previous fall and/or fracture history	2024	10.1016/j.archger.2023.105221	One or more keywords are missing
Deguchi K	et al.	Chrebp Deletion and Mild Protein Restriction Additively Decrease Muscle and Bone Mass and Function	2025	10.3390/nu17030488	One or more keywords are missing
Deldicque L	et al.	Endoplasmic reticulum stress in human skeletal muscle: Any contribution to sarcopenia?	2013	10.3389/fphys.2013.00236	Review
Dempewolf S	et al.	What Are the Barriers to Incorporating Nutrition Interventions Into Care of Older Adults With Femoral Fragility Fractures?	2023		Review
Dennison E	et al.	Osteosarcopenia in the rheumatology clinic,	2019	10.1093/rheumatology/kez109.025	Conference Abstract
Dhote V	et al.	Aging of brain related with mitochondrial dysfunctions	2021	10.2174/138945012199920120920224 7	Review
Di Filippo E	et al.	Myomir dysregulation and reactive oxygen species in aged human satellite cells	2016	10.1016/j.bbrc.2016.03.030	One or more keywords are missing
Di Monaco M	et al.	Sarcopenia, osteoporosis and the burden of prevalent vertebral fractures: a cross-sectional study of 350 women with hip fracture	2020	10.23736/S1973-9087.20.05991-2	One or more keywords are missing
Di Monaco M	et al.	Is there a definition of low lean mass that captures the associated low bone mineral density? A cross-sectional study of 80 men with hip fracture	2018	10.1007/s40520-018-1058-y	One or more keywords are missing
Di Monaco M	et al.	Sarcopenia, osteoporosis and the burden of prevalent vertebral fractures: a cross-sectional	2020	10.23736/S1973-9087.20.05991-2	One or more keywords are missing

		study of 350 women with hip fracture			
DinÃ§el A	et al.	SARCOPENIA AND NEW BIOMARKERS	2023	10.1007/s40520-023-02442-7	Conference Abstract
Dionyssiotis Y	et al.	Osteosarcopenia School	2022	10.1007/s40520-022-02147-3	Conference Abstract
Dionyssiotis Y	et al.	Osteosarcopenia and neurodisability	2022	10.1007/s00198-021-06123-x	Conference Abstract
Dobrowolny G	et al.	The Role of Skeletal Muscle in Neuromuscular Diseases: From Cellular and Molecular Players to Therapeutic Interventions	2022	10.3390/cells11071207	Editorial
Dobrowolny G	et al.	Age-related alterations at neuromuscular junction: Role of oxidative stress and epigenetic modifications	2021	10.3390/cells10061307	Review
Dong D	et al.	Research progress on the osteosarcopenia,	2023	10.3760/cma.j.cn311282-20230130-00046	One or more keywords are missing
Dovjak P	et al.	Prediction of Fragility Fractures and Mortality in a Cohort of Geriatric Patients,	2024	10.1002/jcsm.13631	One or more keywords are missing
Dowling L	et al.	MicroRNAs in obesity, sarcopenia, and commonalities for sarcopenic obesity: a systematic review	2022	10.1002/jcsm.12878	Review
Drey M	et al.	Osteosarcopenia	2021	10.1007/s00108-021-01025-w	Review
Drey M	et al.	Assessment of Bone and Muscle Measurements by Peripheral Quantitative Computed Tomography in Geriatric Patients	2020	10.1016/j.jocd.2018.10.002	One or more keywords are missing
Drey M	et al.	Osteosarcopenia is more than sarcopenia and osteopenia alone	2016	10.1007/s40520-015-0494-1	One or more keywords are missing
Drey M	et al.	Assessment of Bone and Muscle Measurements by Peripheral Quantitative Computed Tomography in Geriatric Patients	2020	10.1016/j.jocd.2018.10.002	One or more keywords are missing

Drummond M	et al.	Aging and microRNA expression in human skeletal muscle: A microarray and bioinformatics analysis	2011	10.1152/physiolgenomics.00148.2010	One or more keywords are missing
Du Y	et al.	The Function of Body Mass Index in the Older with Osteosarcopenia: A Systematic Review and Meta-analysis	2023	10.7570/jomes22057	One or more keywords are missing
Duan X	et al.	Low geriatric nutritional risk index is associated with osteosarcopenia in older patients with type 2 diabetes mellitus	2024	10.1186/s12891-024-08091-9	One or more keywords are missing
Duque G	et al.	Physiology and pathophysiology of circulating osteoprogenitor cells	2023		Conference Abstract
Duque G	et al.	The role of extracellular vesicles in the pathophysiology and treatment of osteosarcopenia	2022	10.1007/s00198-021-06123-x	Conference Abstract
Duque G	et al.	Response to the comment on 'Osteosarcopenia: A Geriatric Giant of the XXI Century	2021	10.1007/s12603-021-1661-z	Letter
Duque G	et al.	Osteosarcopenia: A Geriatric Giant of the XXI Century	2021	10.1007/s12603-021-1640-4	Editorial
Duque G	et al.	Fat as a therapeutic target in osteosarcopenia	2020	10.1007/s00198-020-05695-4	Conference Abstract
Duque G	et al.	Beyond energy regulation: New insights into fat, muscle, and bone interactions	2019	10.002/jbm4.10260	Conference Abstract
Duque G	et al.	Bone and muscle as a whole: Osteosarcopenia	2019		Conference Abstract
Duque G	et al.	Meet-the-expert session: Osteosarcopenia	2019	10.1007/s00198-018-04809-3	Conference Abstract
Duque G	et al.	Osteosarcopenia: Where bone, muscle and fat collide	2019	10.1007/s00198-019-04986-9	Conference Abstract
Duque G	et al.	Pharmacological treatment of	2018	10.1007/s00198-018-4439-3	Conference Abstract

		osteosarcopenia: How to target bone and muscle at the same time?			
Duque G	et al.	Sarco-osteopeniavs. sarco-osteoporosis: Towardsaunified definitionofosteosar-copenia,	2017	10.1007/s00198-017-3950-2	Conference Abstract
Duque G	et al.	Targeting osteosarcopenia: A practical approach for the prevention of falls and osteoporotic fractures	2015	10.1007/s00198-015-3062-9	Conference Abstract
Ebner N	et al.	Silver linings on the horizon: highlights from the 10th Cachexia Conference	2018	10.1002/jcsm.12290	Conference Pa[illegible]
Ebner N	et al.	Recent developments in the field of cachexia, sarcopenia, and muscle wasting: highlights from the 11th Cachexia Conference	2019	10.1002/jcsm.12408	Conference Pa[illegible]
El Miedany Y	et al.	Therapeutic approaches to osteosarcopenia: Denosumab effect on falls risk, physical performance and walking speed	2020	10.1136/annrheumdis-2020-eular.6521	Conference Abstract
Erdogan T	et al.	Some aspects need to be considered in assessment and treatment of sarcopenia	2018	10.2147/CIA.S176216	Letter
Erickson K	et al.	Bone Mineral Density and Muscle Mass in Masters Olympic Weightlifters and Runners	2020	10.1123/japa.2019-0426	One or more keywords are missing
Eroğlu I	et al.	Nonalcoholic Fatty Liver Disease, Bone and Muscle Quality in Prolactinoma: A Pilot Study	2024	10.1016/j.jocd.2024.101479	One or more keywords are missing
Eroğlu I	et al.	Osteosarcopenia in acromegaly: reduced muscle quality and increased vertebral fat deposition	2023	10.1007/s40618-023-02114-3	One or more keywords are missing

Estevinho M et al.	Role of ATP-binding Cassette Transporters in Sorafenib Therapy for Hepatocellular Carcinoma: An Overview	2022	10.2174/138945012266621041212501 8	Review
Fagundes Belchior G et al.	Osteosarcopenia: beyond age-related muscle and bone loss	2020	10.1007/s41999-020-00355-6	Review
Fahimfar N et al.	The Association of Cardiovascular Diseases Risk Scores and Osteosarcopenia Among Older Adult Populations: The Results of Bushehr Elderly Health (BEH) Program	2023	10.1007/s00223-022-01059-8	One or more keywords are missing
Fahimfar N et al.	The association of cardio-metabolic risk factors and history of falling in men with osteosarcopenia: a cross-sectional analysis of Bushehr Elderly Health (BEH) program	2022	10.1186/s12877-021-02657-1	One or more keywords are missing
Fahimfar N et al.	The association of osteosarcopenia and who cardiovascular risk scores in the elderly population: Results from bushehr elderly health (BEH) program	2022	10.1007/s00198-021-06125-9	Conference Abstract
Fahimfar N et al.	Prevalence of Osteosarcopenia and Its Association with Cardiovascular Risk Factors in Iranian Older People: Bushehr Elderly Health (BEH) Program	2020	10.1007/s00223-019-00646-6	One or more keywords are missing
Fan J et al.	MicroRNA-regulated proinflammatory cytokines in sarcopenia	2016	10.1155/2016/1438686	Review
Fang XY et al.	The efficacy of nutritional screening indexes in predicting the incidence of osteosarcopenia and major osteoporotic fracture in the elderly	2024	10.1007/s00774-024-01514-6	One or more keywords are missing

Fanò-Illic G	et al.	Editorial for the special issue "molecular bases of senescence"	2021	10.3390/ijms222111873	Editorial
Fariyike B	et al.	Role of MicroRNA-141 in the Aging Musculoskeletal System: A Current Overview	2019	10.1016/j.mad.2018.12.001	Review
Fathi M	et al.	Association between biomarkers of bone health and osteosarcopenia among Iranian older people: The Bushehr Elderly Health (BEH) program	2021	10.1186/s12877-021-02608-w	One or more keywords are missing
Fatima M	et al.	Osteosarcopenia and Frailty Risk in Community-Dwelling Older Adults: A Follow-Up of the I-Lan Longitudinal Aging Study	2025	10.2139/ssrn.5104021	One or more keywords are missing
Fatima M	et al.	Therapeutic approaches to osteosarcopenia: insights for the clinician	2019	10.1177/1759720X19867009	Review
Feehan J	et al.	Blood borne bone: Circulating Osteoprogenitors Associated with Bone and Muscle quality	2020	10.1002/jbmr.4206	Conference Abstract
Feehan J	et al.	Targeting fundamental aging mechanisms to treat osteoporosis	2019	10.1080/14728222.2019.1702973	Review
Feklistov A	et al.	Pathological phenotypes of body composition in patients with rheumatoid arthritis	2021	10.1136/annrheumdis-2021-eular.3381	Conference Abstract
Feklistov A	et al.	Osteoporosis, sarcopenia and osteosarcopenia in women with rheumatoid arthritis	2018	10.1136/annrheumdis-2018-eular.6141	Conference Abstract
Fernandes S	et al.	Cut-off points to screening for sarcopenia in community-dwelling older people residents in Brazil	2021	10.7717/peerj.12038	Review

Ferrara G	et al.	Abstracts of the Fourth Brainstorming Research Assembly for Young Neuroscientists (BraYn), Italy, 20–22 October 2021	2022	10.3390/neurolint14010010	One or more keywords are missing
Filoni G	et al.	Distrectual osteosarcopenia in limb disuse: case report and mini literature review	2020	10.23750/abm.v91i14-S.10785	One or more keywords are missing
Finke D	et al.	Cancer—A Major Cardiac Comorbidity With Implications on Cardiovascular Metabolism	2021	10.3389/fphys.2021.729713	Review
Fochi S	et al.	Regulation of micrornas in satellite cell renewal, muscle function, sarcopenia and the role of exercise	2020	10.3390/ijms21186732	Review
Foessl I	et al.	A perspective on muscle phenotyping in musculoskeletal research	2024	10.1016/j.tem.2024.01.004	Review
Franceschini G	et al.	Innovations in the Integrated Management of Breast Cancer	2022	10.3390/jpm12040531	Editorial
Franulic F	et al.	Deciphering Osteosarcopenia through the hallmarks of aging,	2024	10.1016/j.mad.2024.111997	One or more keywords are missing
Freitas RVM	et al.	Fourier-Transform Infrared Spectroscopy as a Screening Tool for Osteosarcopenia in Community-Dwelling Older Women	2023	10.1093/gerona/glad081	One or more keywords are missing
Fries N	et al.	Using CT imaging to identify sarcopenia as a risk factor for severe falls in older adults,	2025	10.1186/s12877-025-05707-0	One or more keywords are missing
Frisoli A	et al.	Obese osteosarcopenia with impairment of mobility and weakness in outpatient older adults,	2019	10.1007/s00198-019-04993-w	Conference Abstract
Frisoli A	et al.	Osteosarcopenia phenotype and frailty status by chs and sof criteria,	2019	10.1007/s00198-019-04993-w	Conference Abstract

Frisoli A	et al.	Clinical and biochemical phenotype of osteosarcopenia,	2017	10.1007/s00198-017-3943-1	Conference Abstract
Frisoli A	et al.	Association of Osteosarcopenia, Sarcopenia EWGSOP alone and osteoporosis alone with mobility in older adults: Data from sarcos study,	2017	10.1111/(ISSN)1445-5994	Conference Abstract
Frisoli A	et al.	Body composition phenotype of osteosarcopenia, osteoporosis and sarcopenia: SARCOS study,	2017	10.1002/jbmr.3107	Conference Abstract
Frisoli A	et al.	The association of osteosarcopenia, sarco-penia and osteoporosis with weakness and mobility in older adults with cardi-ovascular disease: Data from sarcos study,	2017	10.1007/s00198-017-3950-2	Conference Abstract
Frisoli A	et al.	Osteosarcopenia is more associated with disability compared to sarcopenia or osteoporosis alone, in older adults with cardiovascular disease: Data from sarcos study,	2017	10.1007/s00198-017-3950-2	Conference Abstract
Frisoli A	et al.	Are osteosarcopenia, sarcopenia and osteoporosis different syndromes? Results from the women's health and aging study (WHAS) II,	2012	10.1111/j.1532-5415.2012.04000.x	Conference Abstract
Fu W	et al.	The regulatory network of potential transcription factors and MiRNAs of mitochondria-related genes for sarcopenia	2022	10.3389/fgene.2022.975886	One or more keywords are missing
Fu Z	et al.	Progress in mechanism of lncRNA-mediated ceRNA network in sarcopenia	2022	10.3969/j.issn.1000-4718.2022.09.020	One or more keywords are missing
Fujimoto T	et al.	Osteosarcopenia: the coexistence of sarcopenia and osteopenia is predictive of prognosis	2025	10.1007/s00595-024-02883-1	One or more keywords are missing

		and postoperative complications after curative resection for colorectal cancer,			
Fukushima N	et al.	Prognostic significance of preoperative osteosarcopenia on patientâ€™ outcomes after emergency surgery for gastrointestinal perforation,	2024	10.1007/s00595-024-02849-3	One or more keywords are missing
Fulzele S	et al.	Muscle-derived miR-34a increases with age in circulating extracellular vesicles and induces senescence of bone marrow stem cells	2019	10.18632/aging.101874	One or more keywords are missing
Furukawa K	et al.	Occult Vertebral Fracture (OVF) in Patients Who Underwent Hepatectomy for Colorectal Liver Metastasis: Strong Association with Oncological Outcomes,	2023	10.3390/cancers15235513	One or more keywords are missing
Furukawa K	et al.	Osteosarcopenia is a potential predictor for the prognosis of patients who underwent hepatic resection for colorectal liver metastases,	2021	10.1002/ags3.12428	One or more keywords are missing
Gabellini D	et al.	16th Meeting of the Interuniversity Institute of Myology (IIM)-Assisi (Italy), October 17-20, 2019: Foreword, Program and Abstracts	2020	10.4081/ejtm.0.9345	Conference Pa[illegible]
Gabriele F	et al.	Distrectual osteosarcopenia in limb disuse: Case report and mini literature review,	2020	10.23750/abm.v91i14-S.10785	One or more keywords are missing
Gadecka A	et al.	Slowing down ageing: The role of nutrients and microbiota in modulation of the epigenome	2019	10.3390/nu11061251	Review
Gallagher I	et al.	Omics/systems biology and cancer cachexia	2016	10.1016/j.semcdb.2015.12.022	Review

Gallo-Soljancic P	et al.	Age- and sex-related development of osteosarcopenia in the aging Octodon degus rodent model	2025	10.3389/fragi.2025.1486670	One or more keywords are missing
García-Giménez J	et al.	Implementing precision medicine in human frailty through epigenetic biomarkers	2021	10.3390/ijerph18041883	Review
Genest F	et al.	Differential impact of osteoporosis, sarcopenia and obesity on physical performance in aging men,	2021	10.1530/EC-20-0580	One or more keywords are missing
Genest F	et al.	Combined efficacy of different exercise interventions in osteosarcopenic men,	2017	10.1002/jbmr.3363	Conference Abstract
Gerlinger-Romero F	et al.	Dysregulation between TRIM63/FBXO32 expression and soleus muscle wasting in diabetic rats: potential role of miR-1-3p, -29a/b-3p, and -133a/b-3p	2017	10.1007/s11010-016-2910-z	One or more keywords are missing
German I	et al.	New Trends to Treat Muscular Atrophy: A Systematic Review of Epicatechin	2024	10.3390/nu16020326	Review
Ghafouri-Fard S	et al.	Sarcopenia and noncoding RNAs: A comprehensive review	2023	10.1002/jcp.31031	Review
Ghafouri-Fard S	et al.	Emerging Role of Non-Coding RNAs in Senescence	2022	10.3389/fcell.2022.869011	Review
Ghasemikaram M	et al.	Detraining effects on muscle quality in older men with osteosarcopenia. Follow-up of the randomized controlled franconian osteopenia and sarcopenia trial (frost),	2021	10.3390/nu13051528	One or more keywords are missing
Ghasemikaram M	et al.	Effects of 16Â months of high intensity resistance training on thigh muscle fat infiltration in elderly	2021	10.1007/s11357-020-00316-8	One or more keywords are missing

		men with osteosarcopenia,			
Giakoumaki I	et al.	Postnatal Protein Intake as a Determinant of Skeletal Muscle Structure and Function in Mice—A Pilot Study	2022	10.3390/ijms23158815	One or more keywords are missing
Gielen E	et al.	Sarcopenia, osteoporosis and frailty,	2023	10.1016/j.metabol.2023.155638	Review
Giustina A	et al.	Vitamin D and hip protectors in osteosarcopenia: a combined hip fracture preventing approach,	2025	10.1007/s11154-024-09907-8	One or more keywords are missing
Goljanek-Whysall K	et al.	Ageing in relation to skeletal muscle dysfunction: redox homoeostasis to regulation of gene expression	2016	10.1007/s00335-016-9643-x	Review
Gomez F	et al.	Effects of the falls and fractures clinic as an integrated multidisciplinary model of care in Australia: A pre-post study,	2019	10.1136/bmjopen-2018-027013	One or more keywords are missing
Gonera-Furman A	et al.	Osteosarcopenia-The Role of Dual-Energy X-ray Absorptiometry (DXA) in Diagnostics	2022	10.3390/jcm11092522	Review
Gonzalez A	et al.	Characterization of sarcopenia in patients with postmenopausal osteoporosis,	2019	10.1007/s00198-019-04993-w	Conference Abstract
Gonzalez A	et al.	Prevalence of sarcopenia in patients with diagnosis of primary osteoporosis,	2018	10.1007/s00198-018-4465-1	Conference Abstract
Granchi D	et al.	Osteosarcopenia in hip fracture: Taking cues from pathophysiology for clinical practice,	2020		One or more keywords are missing
Grassi F	et al.	Report and Abstracts of the 18th Meeting of the Interuniversity Institute of Myology: Virtual meeting, October 21-24, 2021	2021	10.4081/EJTM.2021.10270	One or more keywords are missing

Grebennikova T	et al.	Osteosarcopenia: Pathogenesis, diagnosis and therapeutic approaches,	2020	10.15690/vramn1243	Review
Gregson C	et al.	The epidemiology of osteoporosis and sarcopenia in a high HIV prevalence setting in rural South Africa: a cross-sectional study,	2021	10.1002/jbm4.10552	Conference Abstract
Gregson C	et al.	Osteosarcopenia in a clinical setting: How is it diagnosed, and what are the clinical consequences?,	2019	10.1093/rheumatology/kez109.026	Conference Abstract
Gruneisen E	et al.	Fat as a Friend or Foe of the Bone,	2024	10.1007/s11914-024-00864-4	One or more keywords are missing
Gu S	et al.	Frontiers and hotspots of adipose tissue and NAFLD: a bibliometric analysis from 2002 to 2022	2023	10.3389/fphys.2023.1278952	One or more keywords are missing
Guarnotta V	et al.	Circulating Irisin levels as a marker of osteosarcopenic-obesity in cushingâ€™s disease,	2020	10.2147/DMSO.S249090	One or more keywords are missing
Gubergrits N	et al.	The digest of investigations in the diagnosis and treatment of pancreatic diseases: what’s new in 2019?	2020	10.30978/MG-2020-1-76	Review
Guerra R	et al.	INFRARED SPECTROSCOPY AS A DIAGNOSTIC TOOL FOR OSTEOSARCOPENIC WOMEN	2022	10.1007/s12603-022-1772-1	Conference Abstract
Gumucio J	et al.	Aging-associated exacerbation in fatty degeneration and infiltration after rotator cuff tear	2014	10.1016/j.jse.2013.04.011	One or more keywords are missing
Gunawardene P	et al.	Age, gender, and percentage of circulating osteoprogenitor (COP) cells: The COP Study	2017	10.1016/j.exger.2017.06.004	One or more keywords are missing

Guo R	et al.	Hypoxic preconditioning-engineered bone marrow mesenchymal stem cell-derived exosomes promote muscle satellite cell activation and skeletal muscle regeneration via the miR-210-3p/KLF7 mechanism	2024	10.1016/j.intimp.2024.113143	One or more keywords are missing
Hackl M	et al.	The Role of microRNAs in Osteoporosis Diagnostics	2021	10.1055/a-1514-1800	Review
Hackl M	et al.	Circulating microRNAs as novel biomarkers for bone diseases – Complex signatures for multifactorial diseases?	2016	10.1016/j.mce.2015.10.015	Review
Hadzimuratovic B	et al.	Longitudinal course of circulating miRNAs in a patient with hypophosphatasia and asfotase alfa treatment: a case report	2024	10.1093/jbmrpl/ziae107	One or more keywords are missing
Haeri N	et al.	Does Zoledronic Acid Improve Appendicular Lean Mass in Older Women with Osteoporosis? A Sub-Analysis of a Randomized Clinical Trial	2022	10.14283/jfa.2022.54	One or more keywords are missing
Haffer H	et al.	Osteosarcopenia in the Spine Beyond Bone Mineral Density: Association Between Paraspinal Muscle Impairment and Advanced Glycation Endproducts	2023	10.1097/BRS.0000000000004683	One or more keywords are missing
Haffer H	et al.	Osteosarcopenia in the spine - association between paraspinal muscle impairment and advanced glycation endproducts in lumbar fusion patients	2022	10.1007/s00586-022-07413-6	Conference Abstract
Hamad B	et al.	Osteosarcopenia among postmenopausal women and handgrip strength as	2020	10.1007/s40520-019-01399-w	One or more keywords are missing

		a practical method for predicting the risk			
Hamrick M	et al.	When Wolff's law isn't enough: training students to consider soft tissues in promoting skeletal health	2019	10.1096/fasebj.2019.33.1_supplement.80.1	Conference Abstract
Han K	et al.	The relationship between sarcopenia and osteoporosis in a Korean population-based cohort	2023	10.1016/j.maturitas.2023.04.171	Conference Abstract
Harijanto C	et al.	Does Whole-Body Vibration Training Have a Concurrent Effect on Bone and Muscle Health? A Systematic Review and Meta-Analysis	2022	10.1159/000519511	Review
Hashimoto H	et al.	The Effect of Antihypertensive Therapy on Skeletal Muscle Mass and Bone Mineral Density in Patients With End-Stage Kidney Disease	2024	10.1053/j.jrn.2023.10.008	One or more keywords are missing
Hassan EB	et al.	Osteosarcopenia: A new geriatric syndrome	2017		One or more keywords are missing
Hayasaka T	et al.	Sarcopenia-derived exosomal micro-RNA 16-5p disturbs cardio-repair via a pro-apoptotic mechanism in myocardial infarction in mice	2021	10.1038/s41598-021-98761-8	One or more keywords are missing
He C	et al.	Bone and Muscle Crosstalk in Aging,	2020	10.3389/fcell.2020.585644	Review
He N	et al.	Increasing Fracture Risk Associates With Plasma Circulating MicroRNAs in Aging People's Sarcopenia	2021	10.3389/fphys.2021.678610	One or more keywords are missing
He N	et al.	Circulating MicroRNAs in Plasma Decrease in Response to Sarcopenia in the Elderly	2020	10.3389/fgene.2020.00167	One or more keywords are missing
Heng MWY	et al.	Individual and combined associations of sarcopenia, osteoporosis and obesity with frailty in	2023	10.1186/s12877-023-04500-1	One or more keywords are missing

		a multi-ethnic asian older adult population,			
Henzen C	et al.	[Hormones and Mobility]	2019	10.1024/1661-8157/a003267	One or more keywords are missing
Herrmann M	et al.	The role of bile acid metabolism in bone and muscle: from analytics to mechanisms,	2024	10.1080/10408363.2024.2323132	Review
Heshmat R	et al.	ASSOCIATION BETWEEN MALNUTRITION AND OSTEOSARCOPENIC OBESITY AMONG IRANIAN OLDER PEOPLE: BUSHEHR ELDERLY HEALTH (BEH) PROGRAM	2023	10.1007/s40520-023-02442-7	Conference Abstract
Heshmat R	et al.	ASSESSMENT OF CARDIOVASCULAR DISEASE RISKS USING FRAMINGHAM RISK SCORES AMONG OLDER WOMEN WITH OSTEOSARCOPENIC OBESITY: THE BUSHEHR ELDERLY HEALTH (BEH) PROGRAM	2022	10.1007/s40520-022-02147-3	Conference Abstract
Heshmat R	et al.	Socioeconomic inequalities in osteosarcopenia among community dwelling older people: Findings from the bushehr elderly health (BEH) program	2022	10.1007/s00198-021-06125-9	Conference Abstract
Himoto T	et al.	Current trends of essential trace elements in patients with chronic liver diseases	2020	10.3390/nu12072084	Review
Hirase Y	et al.	Prognostic significance of osteosarcopenia in patients with stage IV gastric cancer undergoing conversion surgery	2025	10.1007/s00423-024-03574-8	One or more keywords are missing
Hirschfeld H	et al.	Osteosarcopenia: where bone, muscle, and fat collide	2017	10.1007/s00198-017-4151-8	Review

Homa-Mlak I	et al.	Three Pathways of Cancer Cachexia: Inflammation, Changes in Adipose Tissue and Loss of Muscle Mass—The Role of miRNAs	2022	10.3390/jpm12091438	Review
Hong A	et al.	Effects of resistance exercise on bone health	2018	10.3803/EnM.2018.33.4.435	Review
Hong S	et al.	Host-specific effects of Eubacterium species on Rg3-mediated modulation of osteosarcopenia in a genetically diverse mouse population	2024	10.1186/s40168-024-01971-1	One or more keywords are missing
Hori M	et al.	Coexistence of Low Muscle Mass and Osteoporosis as a Predictor of Fragility Fractures in Long-Term Kidney Transplant Recipients	2023	10.1159/000534019	One or more keywords are missing
Hosoi T	et al.	Sarcopenia phenotype and impaired muscle function in male mice with fast-twitch muscle-specific knockout of the androgen receptor	2023	10.1073/pnas.2218032120	One or more keywords are missing
Hou J	et al.	Aged bone marrow macrophages drive systemic aging and age-related dysfunction via extracellular vesicle-mediated induction of paracrine senescence	2024	10.1038/s43587-024-00694-0	One or more keywords are missing
Hu K	et al.	Understanding the Consequences of Fatty Bone and Fatty Muscle: How the Osteosarcopenic Adiposity Phenotype Uncovers the Deterioration of Body Composition	2023	10.3390/metabo13101056	Review
Hu M	et al.	Effect and mechanism of miRNA-144-5p-regulated autophagy in older adults with Sarcopenia	2025	10.1186/s12979-025-00499-8	One or more keywords are missing

Hu Z	et al.	MicroRNA-29 induces cellular senescence in aging muscle through multiple signaling pathways	2014	10.18632/aging.100643	One or more keywords are missing
Huang J	et al.	Fibroblast growth factor 9 (FGF9) inhibits myogenic differentiation of C2C12 and human muscle cells	2019	10.1080/15384101.2019.1691796	One or more keywords are missing
Huang L	et al.	Strontium zinc silicate simultaneously alleviates osteoporosis and sarcopenia in tail-suspended rats via Piezo1-mediated Ca(2+) signaling	2024	10.1016/j.jot.2024.07.014	One or more keywords are missing
Huang R	et al.	Lacto-ovo-vegetarian diet is inversely associated with the osteosarcopenia in older adults	2024	10.1186/s12877-024-04959-6	One or more keywords are missing
Huang S	et al.	Identification of the shared gene signatures and pathways between sarcopenia and type 2 diabetes mellitus	2022	10.1371/journal.pone.0265221	One or more keywords are missing
Huang T	et al.	Prevalence and risk factors of osteosarcopenia: a systematic review and meta-analysis	2023	10.1186/s12877-023-04085-9	One or more keywords are missing
Huang W	et al.	A review on the Role of Oral Nutritional Supplements in Chronic Obstructive Pulmonary Disease	2022	10.1007/s12603-022-1822-8	Review
Huh J	et al.	Osteosarcopenia: A new geriatric syndrome with great impact on cancer patients	2022	10.1186/s40644-022-00479-x	Conference Abstract
Huo Y	et al.	Phenotype of sarcopenic obesity in older individuals with a history of falling	2016	10.1016/j.archger.2016.04.003	One or more keywords are missing
Hurtado Y	et al.	Challenges in Delivering Effective Care for Older Persons with Fragility Fractures	2024	10.2147/CIA.S433999	Review

Ibáñez-Ventoso C	et al.	MicroRNAs in C. elegans aging: Molecular insurance for robustness?	2009	10.2174/138920209788185243	Review
Ibrahim K	et al.	The feasibility of assessing sarcopeniaamong older people with arm fractureusing different criteria	2021	10.1093/ageing/afab030.115	Conference Abstract
Ichikawa T	et al.	Grip strength is a strong indicator of osteoporosis in both women and men with chronic liver disease	2025	10.1111/hepr.14179	One or more keywords are missing
Ignatyev O	et al.	POSTMENOPAUSAL OSTEOSARCOPENIA	2023	10.1007/s40520-023-02442-7	Conference Abstract
Ileri I	et al.	The role of thiol-disulfide homeostasis and ischemia-modified albumin in osteosarcopenia	2024	10.1007/s11845-024-03667-9	One or more keywords are missing
Ilich J	et al.	Osteosarcopenic adiposity and its relation to cancer and chronic diseases: Implications for research to delineate mechanisms and improve clinical outcomes	2025	10.1016/j.arr.2024.102601	Review
Ilich J	et al.	Osteosarcopenic adiposity (OSA) phenotype and its connection with cardiometabolic disorders: Is there a cause-and-effect?	2024	10.1016/j.arr.2024.102326	Review
Ilich J	et al.	Osteosarcopenic adiposity syndrome update and the role of associated minerals and vitamins	2021	10.1017/S0029665121000586	Conference Pa[illegible]
Ilich J	et al.	Nutritional and behavioral approaches to body composition and low-grade chronic inflammation management for older adults in the ordinary and covid-19 times	2020	10.3390/nu12123898	Note

Ilich J	et al.	Chronic stress contributes to osteosarcopenic adiposity via inflammation and immune modulation: The case for more precise nutritional investigation	2020	10.3390/nu12040989	Review
Imani M	et al.	Development of a Novel Fully Automatic Segmentation and Quantification Technique for Assessment of Musculoskeletal Organs	2022	10.1007/s40520-022-02147-3	Conference Abstract
Imani M	et al.	Validation of a Semiautomatic Image Analysis Software for the Quantification of Musculoskeletal Tissues	2022	10.1007/s00223-021-00914-4	One or more keywords are missing
Inceoglu SC	et al.	The Impact of Combination of Aerobic and Resistive Exercise on Activities of Daily Living and Risk of Fall in Osteosarcopenic Patients	2024	10.14744/SEMB.2024.56898	One or more keywords are missing
Ingelson-Filpula W	et al.	Muscles in winter: The epigenetics of metabolic arrest	2021	10.3390/EPIGENOMES5040028	Review
Inoue D	et al.	Mechanisms of osteoporosis associated with chronic obstructive pulmonary disease	2024	10.1007/s00774-024-01527-1	Review
Inoue T	et al.	Exploring biomarkers of osteosarcopenia in older adults attending a frailty clinic	2023	10.1016/j.exger.2022.112047	One or more keywords are missing
Inoue T	et al.	Osteosarcopenia, the co-existence of osteoporosis and sarcopenia, is associated with social frailty in older adults	2022	10.1007/s40520-021-01968-y	One or more keywords are missing
Inoue T	et al.	Association between osteosarcopenia and cognitive frailty in older outpatients visiting a frailty clinic	2022	10.1016/j.archger.2021.104530	One or more keywords are missing

Inoue T	et al.	Related factors and clinical outcomes of osteosarcopenia: A narrative review	2021	10.3390/nu13020291	One or more keywords are missing
Intriago M	et al.	Bone Mass Loss and Sarcopenia in Ecuadorian Patients	2020	10.1155/2020/1072675	One or more keywords are missing
Islam M	et al.	SuperAgers and centenarians, dynamics of healthy ageing with cognitive resilience	2024	10.1016/j.mad.2024.111936	One or more keywords are missing
Ito K	et al.	Skeletal Muscle Mass Index Is Positively Associated With Bone Mineral Density in Hemodialysis Patients	2020	10.3389/fmed.2020.00187	One or more keywords are missing
Iwasaki H	et al.	MicroRNA-494 plays a role in fiber type-specific skeletal myogenesis in human induced pluripotent stem cells	2015	10.1016/j.bbrc.2015.10.128	One or more keywords are missing
Jang SY	et al.	Bidirectional crosstalk between bone and muscle: the role of RANKL pathway in osteosarcopenia	2024	10.1530/JOE-24-0093	Review
Javanmardifard Z	et al.	MicroRNAs associated with signaling pathways and exercise adaptation in sarcopenia	2021	10.1016/j.lfs.2021.119926	Review
Jin H	et al.	The role of melatonin in sarcopenia: Advances and application prospects	2021	10.1016/j.exger.2021.111319	Review
Jin J	et al.	Conservative analysis of Synaptopodin-2 intron sense-overlapping lncRNA reveals its novel function in promoting muscle atrophy	2022	10.1002/jcsm.13012	One or more keywords are missing
Johnson K	et al.	Yield and cost-effectiveness of laboratory testing to identify metabolic contributors to falls and fractures in older persons,	2015	10.1007/s11657-015-0226-3	One or more keywords are missing

Johnson T	et al.	A 7T MRI Study of Fibular Bone Thickness and Density: Impact of Age, Sex and Body Weight, and Correlation with Bone Marrow Expansion and Muscle Fat Infiltration,	2025	10.3390/diagnostics15050564	One or more keywords are missing
Jones R	et al.	Biomarkers associated with lower limb muscle function in individuals with sarcopenia: a systematic review	2022	10.1002/jcsm.13064	Review
Jones T	et al.	Osteoporosis, fracture, osteoarthritis & sarcopenia: A systematic review of circulating microRNA association	2021	10.1016/j.bone.2021.116068	Review
José Neto N	et al.	Gut microbiota dysbiosis, sarcopenia, osteoporosis and osteosarcopenia in older people: A systematic review protocol	2025	10.1371/journal.pone.0313193	One or more keywords are missing
Juan Peng D	et al.	Analysis of related factors for sarco-osteoporosis in middle-aged and elderly inpatients and development and validation of a nomogram	2024	10.1186/s12891-023-06991-w	One or more keywords are missing
Jung H	et al.	MicroRNAs in Skeletal Muscle Aging: Current Issues and Perspectives	2019	10.1093/gerona/gly207	Review
Jung W	et al.	MicroRNA mediated regulation of muscular atrophy: Exploring molecular pathways and therapeutics (Review)	2024	10.3892/mmr.2024.13222	Review
Jung W	et al.	Identifying the potential therapeutic effects of miR?6516 on muscle disuse atrophy	2024	10.3892/mmr.2024.13243	One or more keywords are missing
Kai W	et al.	Impact of osteosarcopenia on short- and long-term outcomes in patients with gastric cancer	2025	10.1093/jjco/hyaf003	One or more keywords are missing
Kalhori M	et al.	MiR-1290: a potential therapeutic target for	2023	10.1007/s10238-022-00854-9	Review

		regenerative medicine or diagnosis and treatment of non-malignant diseases			
Kang Y	et al.	Differential gene expression profile by RNA sequencing study of elderly osteoporotic hip fracture patients with sarcopenia,	2021	10.1016/j.jot.2021.04.009	One or more keywords are missing
Kanjanavaikoon N	et al.	Age, body mass index, and function as the independent predictors of sarcopenia in axial spondyloarthritis: a cross-sectional analysis,	2023	10.1007/s10067-023-06770-x	One or more keywords are missing
Kao J	et al.	Highlights	2020	10.1016/j.jfma.2020.06.019	Editorial
Kao J	et al.	Highlights	2022	10.1016/j.jfma.2022.01.010	Editorial
Kara GK	et al.	Effect of osteosarcopenia on the development of a second compression fracture and mortality in elderly patients after vertebroplasty	2023	10.5152/j.aott.2023.23099	One or more keywords are missing
Karacan I	et al.	Exploring neuronal mechanisms of osteosarcopenia in older adults	2024	10.1113/JP285666	One or more keywords are missing
Kaufman J	et al.	Androgens: A role in bone and muscle disorders management?,	2019	10.1007/s00198-019-04980-1	Conference Abstract
Kaya S	et al.	The relationship between frailty and osteosarcopenia in geriatric patients	2017		Conference Abstract
Kazemi M	et al.	Osteosarcopenia in reproductive-aged women with polycystic ovary syndrome: a multicenter case-control study	2020	10.1210/clinem/dgaa426	Review
Kazemi M	et al.	Response to Letter to the Editor from Smith et al: osteosarcopenia in Reproductive-Aged Women with Polycystic Ovary Syndrome: A	2021	,10.1210/clinem/dgaa670	Letter

		Multicenter Case-Control Study			
Kelly OJ	et al.	Osteosarcopenic obesity: Current knowledge, revised identification criteria and treatment principles	2019	10.3390/nu11040747	One or more keywords are missing
Kemmler W	et al.	Changes in body composition and cardiometabolic health after detraining in older men with osteosarcopenia: 6-month follow-up of the randomized controlled franconian osteopenia and sarcopenia trial (frost) study	2021	10.2147/CIA.S299867	One or more keywords are missing
Kemmler W	et al.	Detraining effects after 18 months of high intensity resistance training on osteosarcopenia in older menâ€"Six-month follow-up of the randomized controlled Franconian Osteopenia and Sarcopenia Trial (FrOST)	2021	10.1016/j.bone.2020.115772	One or more keywords are missing
Kemmler W	et al.	Effects of High-Intensity Resistance Training on Osteopenia and Sarcopenia Parameters in Older Men with Osteosarcopeniaâ€"One-Year Results of the Randomized Controlled Franconian Osteopenia and Sarcopenia Trial (FrOST)	2020	10.1002/jbmr.4027	One or more keywords are missing
Kemmler W	et al.	Effects of high intensity dynamic resistance exercise and whey protein supplements on osteosarcopenia in older men with low bone and muscle Mass. Final results of the randomized controlled FrOST study	2020	10.3390/nu12082341	One or more keywords are missing

Kemmler W	et al.	Effects of High-Intensity Resistance Training on Fitness and Fatness in Older Men With Osteosarcopenia	2020	10.3389/fphys.2020.01014	One or more keywords are missing
Kemp P	et al.	Epigenetics and Susceptibility to Muscle Wasting in COPD	2017	10.1016/j.arbr.2017.01.003	One or more keywords are missing
Kern H	et al.	Electrical stimulation (ES) counteracts muscle decline in seniors	2014	10.3389/fnagi.2014.00189	One or more keywords are missing
Keser I	et al.	Assessment of Body Composition and Dietary Intake in Nursing-Home Residents: Could Lessons Learned from the COVID-19 Pandemic Be Used to Prevent Future Casualties in Older Individuals?	2021	10.3390/nu13051510	One or more keywords are missing
Keser I	et al.	Osteosarcopenic adiposity in croatian nursing home residents: indications for increased susceptibility to Covid-19 and possible ways for curtailment	2020	10.1016/j.clnesp.2020.09.847	Conference Abstract
Khan M	et al.	Sarcopenia in Men With Bone-Predominant Metastatic Castration-Resistant Prostate Cancer Undergoing Ra-223 Therapy	2023	10.1016/j.clgc.2023.01.009	One or more keywords are missing
Khan M	et al.	Sarcopenia assessment in men with metastatic castration-resistant prostate cancer (mCRPC) undergoing radium 223 (Ra223) therapy	2020	10.1016/j.annonc.2020.08.922	Conference Abstract
Kim BJ	et al.	Beyond Bone: Embracing Osteosarcopenia for Comprehensive Fracture Prevention	2024	10.3803/EnM.2024.2002	Letter
Kim H	et al.	Narirutin Improves Cisplatin-Induced Osteosarcopenia and Osteoblastogenesis via Wnt/catenin and MAPK	2024	10.1016/j.cdnut.2024.102636	Conference Abstract

		Signaling Pathways by Activating Irisin			
Kim H	et al.	Age Is Just a Number: Progress and Obstacles in the Discovery of New Candidate Drugs for Sarcopenia	2023	10.3390/cells12222608	Review
Kim IS	et al.	Impact of Fat Mass on Osteoporosis, Sarcopenia, and Osteosarcopenia in Peritoneal Dialysis Patients	2024	10.1159/000540948	One or more keywords are missing
Kim KT	et al.	Prevalence and Clinical Implications of Osteosarcopenia in Patients With Acute Stroke: A Cross-sectional Study	2025	10.1097/PHM.0000000000002526	One or more keywords are missing
Kircher K	et al.	Effects of high-intensity training on fatty infiltration in paraspinal muscles in elderly males with osteosarcopenia - the randomized controlled FrOST study	2024	10.1186/s12877-024-04736-5	One or more keywords are missing
Kirk B	et al.	Leukocyte telomere length is associated with MRI-thigh fat-free muscle volume: data from 16 356 UK Biobank adults	2024	10.1002/jcsm.13461	One or more keywords are missing
Kirk B	et al.	Comparing the Fracture Profile of Osteosarcopenic Older Adults with Osteopenia/Osteoporosis Alone	2023	10.1007/s00223-022-01044-1	One or more keywords are missing
Kirk B	et al.	Serum levels of C-Terminal Telopeptide (CTX) are Associated with Muscle Function in Community-Dwelling Older Adults	2022	10.1093/gerona/glac008	One or more keywords are missing
Kirk B	et al.	Associations between leukocyte telomere length and osteosarcopenia in 20,400 adults aged 60 years and over: Data from the UK Biobank	2022	10.1016/j.bone.2022.116425	One or more keywords are missing

Kirk B	et al.	Nutrients to mitigate osteosarcopenia: the role of protein, vitamin D and calcium	2021	10.1097/MCO.0000000000000711	Review
Kirk B	et al.	A clinical guide to the pathophysiology, diagnosis and treatment of osteosarcopenia	2020	10.1016/j.maturitas.2020.05.012	Review
Kirk B	et al.	Muscle, Bone, and Fat Crosstalk: the Biological Role of Myokines, Osteokines, and Adipokines	2020	10.1007/s11914-020-00599-y	Review
Kirk B	et al.	Osteosarcopenia: epidemiology, diagnosis, and treatmentâ€"facts and numbers	2020	10.1002/jcsm.12567	Editorial
Kirk B	et al.	Osteosarcopenia impairs balance in community-dwelling older adults	2019	10.002/jbm4.10300	Conference Abstract
Kirk B	et al.	Osteosarcopenia: A case of geroscience	2019	10.1002/agm2.12080	Review
Kirk B	et al.	Muscle, Bone, and Fat Crosstalk: the Biological Role of Myokines, Osteokines, and Adipokines	2020	10.1007/s11914-020-00599-y	One or more keywords are missing
Kirk B	et al.	Nutrients to mitigate osteosarcopenia: the role of protein, vitamin D and calcium	2021	10.1097/MCO.0000000000000711	One or more keywords are missing
Kirk B	et al.	Leukocyte telomere length is associated with MRI-thigh fat-free muscle volume: data from 16 356 UK Biobank adults	2024	10.1002/jcsm.13461	One or more keywords are missing
Klein G	et al.	Pharmacologic Treatments to Preserve Bone and Muscle Mass in Osteosarcopenia,	2020	10.1007/s11914-020-00576-5	Review
Knauer K	et al.	Effects of High-Intensity Resistance Training on Visceral Adipose Tissue and Abdominal Aortic Calcifications in Older Men with Osteosarcopenia â€"	2023	10.2147/CIA.S388026	One or more keywords are missing

		Results from the FrOST Study			
Kobayashi K	et al.	Epidemiology and effect on physical function of osteosarcopenia in community-dwelling elderly people in Japan	2020	10.1080/14397595.2019.1623455	One or more keywords are missing
Kolenda Paulin T	et al.	Reply to Letter to the Editor: Osteosarcopenia: Adjust for the BMI or Bare the Bias	2024	10.1007/s00223-024-01223-2	Letter
Kolodziej F	et al.	MicroRNAs as the Sentinels of Redox and Hypertrophic Signalling	2022	10.3390/ijms232314716	Review
Kong MTK	et al.	Evaluation of using grip strength and hand muscle cross-sectional area to predict secondary fractures post distal radius fracture	2025	10.1007/s11657-024-01465-5	One or more keywords are missing
Kositsawat J	et al.	Nutrients with anabolic/anticatabolic, antioxidant, and anti-inflammatory properties: Targeting the biological mechanisms of aging to support musculoskeletal health	2021	10.1016/j.exger.2021.111521	Review
Kostoglou-Athanassiou I	et al.	Osteosarcopenia	2020	10.1007/s00198-020-05695-4	Conference Abstract
Kostoglou-Athanassiou I	et al.	Editorial: (Osteo)sarcopenia & sarcopenic obesity, volume II	2023	10.3389/fendo.2023.1309645	Editorial
Kottorou A	et al.	Non-coding RNAs in cancer-associated cachexia: clinical implications and future perspectives	2021	10.1016/j.tranon.2021.101101	Review
Kozlova IV	et al.	Osteosarcopenia in chronic pancreatitis,	2021	10.26442/00403660.2021.08.200971	One or more keywords are missing
Krauss T	et al.	Specific miRNAs are associated with human cancer cachexia in an organ-specific manner	2023	10.1002/jcsm.13224	One or more keywords are missing

Krikelis M	et al.	LOW BIOAVAILABLE IGF-1 IS THE SINGLE BIOMARKER ASSOCIATED WITH OSTEOSARCOPENIA IN POSTMENOPAUSAL WOMEN WITH RHEUMATOID ARTHRITIS,	2022	10.1007/s40520-022-02147-3	Conference Abstract
Kumar P	et al.	Saliva as a potential non-invasive liquid biopsy for early and easy diagnosis/prognosis of head and neck cancer	2024	10.1016/j.tranon.2023.101827	One or more keywords are missing
Kuo CL	et al.	Very Low and High Levels of Vitamin D Are Associated with Shorter Leukocyte Telomere Length in 148,321 UK Biobank Participants,	2023	10.3390/nu15061474	One or more keywords are missing
Kutsal YG	et al.	Osteosarcopenia: Clinical Perspective,	2020	10.4274/tod.galenos.2020.65477	Review
Kuzma M	et al.	Predictive value of microRNAs in the diagnosis and prognosis of heart failure and osteoporosis	2017		Review
Kweon S	et al.	Sarcopenia and Its Association With Change of Bone Mineral Density and Functional Outcome in Old-Aged Hip Arthroplasty Patients	2022	10.1177/21514593221092880	One or more keywords are missing
LÃ³pez-Teros M	et al.	The Association of Osteosarcopenia With Functional Disability in Community-Dwelling Mexican Adults 50 and Older,	2021	10.3389/fmed.2021.674724	One or more keywords are missing
Lang S	et al.	Prognostic biomarkers for cholangiocarcinoma (CCA): state of the art	2021	10.1080/17474124.2021.1912591	One or more keywords are missing
Larijani B	et al.	Cardio-metabolic risk factors of falling in men with osteosarcopenia: The results of Bushehr Elderly Health (BEH) program,	2020		Conference Abstract

Larijani B	et al.	Association of osteosarcopenia and cognitive impairment in a community dwelling older population: The Bushehr Elderly Health (BEH) program,	2018		Conference Abstract
Laskou F	et al.	Associations of osteoporosis and sarcopenia with frailty and multimorbidity among participants of the Hertfordshire Cohort Study,	2022	10.1002/jcsm.12870	One or more keywords are missing
Laskou F	et al.	A pas de deux of osteoporosis and sarcopenia: osteosarcopenia,	2022	10.1080/13697137.2021.1951204	Review
Lathigara D	et al.	Molecular Mechanisms of Western Diet-Induced Obesity and Obesity-Related Carcinogenesis—A Narrative Review	2023	10.3390/metabo13050675	Review
Laurent M	et al.	Age-related bone loss and sarcopenia in men,	2019	10.1016/j.maturitas.2019.01.006	Review
Laurent M	et al.	Androgens have antiresorptive effects on trabecular disuse osteopenia independent from muscle atrophy,	2016	10.1016/j.bone.2016.09.011	One or more keywords are missing
Laurent M	et al.	Muscle-bone interactions: From experimental models to the clinic? A critical update,	2016	10.1016/j.mce.2015.10.017	One or more keywords are missing
Lee A	et al.	Associations between Osteosarcopenia and Falls, Fractures, and Frailty in Older Adults: Results From the Canadian Longitudinal Study on Aging (CLSA),	2024	10.1016/j.jamda.2023.09.027	One or more keywords are missing
Lee BC	et al.	Effects of resistance training and nutritional support on osteosarcopenia in older, community-dwelling postmenopausal Korean	2024	10.1186/s12877-024-04667-1	One or more keywords are missing

		females (ERTO-K study): a study protocol,			
Lee BC	et al.	Physical activity and osteosarcopenia in Korean adults aged 65 years and older: a national cross-sectional study using the KNHANES data,	2023	10.1186/s12877-023-04121-8	One or more keywords are missing
Lee I	et al.	Exenatide reverses dysregulated microRNAs in high-fat diet-induced obese mice	2016	10.1016/j.orcp.2015.07.011	One or more keywords are missing
Lee J	et al.	Role of MicroRNAs and Long Non-Coding RNAs in Sarcopenia	2022	10.3390/cells11020187	Review
Lee K	et al.	Association of osteosarcopenic obesity and its components: osteoporosis, sarcopenia and obesity with insulin resistance,	2020	10.1007/s00774-020-01104-2	One or more keywords are missing
Lee K	et al.	microRNA for determining the age-related myogenic capabilities of skeletal muscle	2015	10.5483/BMBRep.2015.48.11.211	One or more keywords are missing
Lee S	et al.	Daily Walking Accompanied with Intermittent Resistance Exercise Prevents Osteosarcopenia: A Large Cohort Study,	2022	10.11005/jbm.2022.29.4.255	One or more keywords are missing
Lee S	et al.	Epidemiology and effect on physical function of osteosarcopenia in patients with endstage knee osteoarthritis,	2020	10.1007/s00198-020-05696-3	Conference Abstract
Lee YH	et al.	Effects of progressive elastic band resistance exercise for aged osteosarcopenic adiposity women,	2021	10.1016/j.exger.2021.111272	One or more keywords are missing
Lei T	et al.	Genetic Influence of the Brain on Muscle Structure: A Mendelian	2025	10.1002/jcsm.13647	One or more keywords are missing

		Randomization Study of Sarcopenia			
Leser JM	et al.	Aging, Osteo-Sarcopenia, and Musculoskeletal Mechano-Transduction	2021	10.3389/fresc.2021.782848	One or more keywords are missing
Levinger I	et al.	Sarcopenia and Osteoporotic Fractures,	2016	10.1007/s12018-016-9204-6	Review
Lewiecki E	et al.	Proceedings of the 2022 Santa Fe Bone Symposium: Current Concepts in the Care of Patients with Osteoporosis and Metabolic Bone Diseases,	2022	10.1016/j.jocd.2022.10.002	Review
Lewis A	et al.	Increased expression of H19/miR-675 is associated with a low fat-free mass index in patients with COPD	2016	10.1002/jcsm.12078	One or more keywords are missing
Li C	et al.	Exosomal microRNAs in cancer-related sarcopenia: Tumor-derived exosomal microRNAs in muscle atrophy	2021	10.1177/1535370221990322	Review
Li G	et al.	Connexin 43 Channels in Osteocytes Are Necessary for Bone Mass and Skeletal Muscle Function in Aged Male Mice,	2022	10.3390/ijms232113506	One or more keywords are missing
Li H	et al.	Autophagy in striated muscle diseases	2022	10.3389/fcvm.2022.1000067	Review
Li S	et al.	Advances in the Study of Denosumab Treatment for Osteoporosis and Sarcopenia in the Chinese Middle-Aged and Elderly Population,	2024	10.2147/IJGM.S494759	Review
Li W	et al.	Vitamin D combined with whole-body vibration training for the treatment of osteo-sarcopenia: study protocol for a randomized controlled trial,	2024	10.1186/s13063-024-08498-8	One or more keywords are missing
Li W	et al.	MRI-based vertebral bone quality score is a comprehensive index reflecting the quality of	2024	10.1016/j.spinee.2023.11.007	One or more keywords are missing

		bone and paravertebral muscle,			
Li W	et al.	MRI AND QCT WERE USED TO ANALYZE MUSCULOSKELETAL CORRELATION IN PATIENTS WITH OSTEO-SARCOPENIA,	2023	10.1007/s40520-023-02442-7	Conference Abstract
Li X	et al.	Osteosarcopenia: Muscle-bone interactions,	2022	10.12307/2022.365	One or more keywords are missing
Li Y	et al.	Potential application of anti-osteoporotic therapy to relieve sarcopenia in the elderly,	2023	10.1097/MS9.0000000000001352	Review
Li Y	et al.	Myoblast-derived exosomal Prrx2 attenuates osteoporosis via transcriptional regulation of lncRNA-MIR22HG to activate Hippo pathway	2023	10.1186/s10020-023-00649-y	Irrelevant
Li Y	et al.	Targeting Protein Phosphatases for the Treatment of Chronic Liver Disease	2024	10.2174/0113894501278886231221092522	Review
Li Z	et al.	BMSC-Derived Exosomes Inhibit Dexamethasone-Induced Muscle Atrophy via the miR-486-5p/FoxO1 Axis	2021	10.3389/fendo.2021.681267	One or more keywords are missing
Li Z	et al.	LncIRS1 controls muscle atrophy via sponging miR-15 family to activate IGF1-PI3K/AKT pathway	2019	10.1002/jcsm.12374	One or more keywords are missing
Liang C	et al.	Recent advances in the diagnostic and therapeutic roles of microRNAs in colorectal cancer progression and metastasis	2022	10.3389/fonc.2022.911856	Review
Liang J	et al.	MicroRNA profiling of different exercise interventions for alleviating skeletal muscle atrophy in naturally aging rats	2023	10.1002/jcsm.13137	One or more keywords are missing

Liang J	et al.	Regulatory roles of microRNAs in sarcopenia and exercise intervention	2020	10.13294/j.aps.2020.0061	Review
Lichtenberg T	et al.	The favorable effects of a high-intensity resistance training on sarcopenia in older community-dwelling men with osteosarcopenia: The randomized controlled frost study,	2019	10.2147/CIA.S225618	One or more keywords are missing
Lima R	et al.	Stages of sarcopenia, bone mineral density, and the prevalence of osteoporosis in older women,	2019	10.1007/s11657-019-0591-4	One or more keywords are missing
Lin H	et al.	The effect of rapamycin and its analogues on age-related musculoskeletal diseases: a systematic review,	2022	10.1007/s40520-022-02190-0	Review
Lin YH	et al.	The Impact of the "Osteo" Component of Osteosarcopenia on Fragility Fractures in Post-Menopausal Women	2021	10.3390/ijms22105256	One or more keywords are missing
Lippi L	et al.	Osteosarcopenia in Patients with Chronic Obstructive Pulmonary Diseases: Which Pathophysiologic Implications for Rehabilitation?,	2022	10.3390/ijerph192114314	Review
Lissens M	et al.	Neurogenic osteoporosis due to sarcopenia in post poliomyelitis patients,	2020	10.1007/s00198-020-05695-4	Conference Abstract
Liu C	et al.	Osteoporosis and sarcopenia-related traits: A bi-directional Mendelian randomization study,	2022	10.3389/fendo.2022.975647	One or more keywords are missing
Liu D	et al.	Identification of the molecular link: STAT3 is a shared key gene linking postmenopausal osteoporosis and sarcopenia	2024	10.1302/2046-3758.138.BJR-2023-0351.R2	One or more keywords are missing

Liu H	et al.	Circulating MicroRNA-486 and MicroRNA-146a serve as potential biomarkers of sarcopenia in the older adults	2021	10.1186/s12877-021-02040-0	One or more keywords are missing
Liu J	et al.	β-Cell function is associated with osteosarcopenia in middle-aged and older nonobese patients with type 2 diabetes: A cross-sectional study	2021	10.1515/med-2021-0376	One or more keywords are missing
Liu L	et al.	Osteosarcopenia and its outcomes in Taiwan,	2019		Conference Abstract
Liu W	et al.	Current perspective on the regulation of FOXO4 and its role in disease progression	2020	10.1007/s00018-019-03297-w	Review
Liu Y	et al.	Research Progress on Emerging Signaling Pathways Related to Muscle Bone Symbiosis,	2024	10.12290/xhyxzz.2023-0277	Review
Liu Y	et al.	Effect of sarcopenia, osteoporosis, and osteosarcopenia on spine fracture in American adults with prediabetes,	2023	10.3389/fendo.2023.1163029	One or more keywords are missing
Liu Y	et al.	[Research progress on the role of regulatory T cells/Th17 cells balance in muscle-bone degeneration and reconstruction]	2024		One or more keywords are missing
Lombardo M	et al.	Functional Role of Extracellular Vesicles in Skeletal Muscle Physiology and Sarcopenia: The Importance of Physical Exercise and Nutrition	2024	10.3390/nu16183097	Review
Loncar G	et al.	Emerging biomarkers in heart failure and cardiac cachexia	2014	10.3390/ijms151223878	Review
Long Y	et al.	Does exercise influence skeletal muscle by modulating mitochondrial functions via regulating	2023	10.1016/j.arr.2023.102048	Review

		MicroRNAs? A systematic review			
Long Y	et al.	Low-Magnitude High-Frequency Vibration Attenuates Sarcopenia by Modulating Mitochondrial Quality Control via Inhibiting miR-378	2025	10.1002/jcsm.13740	Review
López-Teros MT	et al.	The Association of Osteosarcopenia With Functional Disability in Community-Dwelling Mexican Adults 50 and Older	2021	10.3389/fmed.2021.674724	One or more keywords are missing
Lu Y	et al.	Sex-and age-related changes in body composition among population-based healthy Chinese in Taiwan,	2018		Conference Abstract
Ma H	et al.	Human umbilical cord mesenchymal stem cell-derived exosomes ameliorate muscle atrophy via the miR-132-3p/FoxO3 axis	2024	10.1016/j.jot.2024.08.005	One or more keywords are missing
Ma J	et al.	Zhuanggu Zhitong Capsule alleviates osteosarcopenia in rats by up-regulating PI3K/Akt/Bcl2 signaling pathway,	2021	10.1016/j.biopha.2021.111939	One or more keywords are missing
Ma J	et al.	The impact of mRNA turnover and translation on age-related muscle loss	2012	10.1016/j.arr.2012.05.004	Review
Ma X	et al.	The Creation of a Rat Model for Osteosarcopenia via Ovariectomy	2025	10.3791/67539	One or more keywords are missing
Ma X	et al.	Lnc-MEG8 regulates yak myoblast differentiation via the miR-22-3p/RTL1 axis	2024	10.1186/s12864-024-11038-y	One or more keywords are missing
Ma Y	et al.	Adherence to a healthy dietary pattern mitigates the detrimental associations between osteosarcopenic adiposity	2025	10.1039/d4fo03732d	One or more keywords are missing

		and both all-cause mortality and life expectancy: a cohort study,			
MacDonald E	et al.	TGF? signaling: Its role in fibrosis formation and myopathies	2012	10.1097/BOR.0b013e328358df34	Review
Madrid-García A	et al.	Understanding the role and adoption of artificial intelligence techniques in rheumatology research: An in-depth review of the literature	2023	10.1016/j.semarthrit.2023.152213	Review
Maghbooli Z	et al.	The lower basal metabolic rate is associated with increased risk of osteosarcopenia in postmenopausal women,	2022	10.1186/s12905-022-01754-6	One or more keywords are missing
Majumder P	et al.	Prevalence of sarcopenia in osteoporosis and osteopenia patients: Study in a tertiary level hospital in Bangladesh,	2020	10.1007/s00198-020-05696-3	Conference Abstract
Makida K	et al.	Low energy irradiation of narrow-range UV-LED prevents osteosarcopenia associated with vitamin D deficiency in senescence-accelerated mouse prone 6,	2020	10.1038/s41598-020-68641-8	One or more keywords are missing
Mancuso S	et al.	Bone damage and health-related quality of life in Hodgkin lymphoma survivors: closing the gaps,	2024	10.3389/fonc.2024.1201595	Review
Mandelli A	et al.	The role of estrogens in osteosarcopenia: from biology to potential dual therapeutic effects,	2022	10.1080/13697137.2021.1965118	Review
Marc F	et al.	Osteoporosis and sarcopenia in patients with liver cirrhosis,	2019	10.1007/s00198-019-04993-w	Conference Abstract
Margolis L	et al.	Potential Role of MicroRNA in the Anabolic Capacity of Skeletal Muscle with Aging	2018	10.1249/JES.0000000000000147	Review

Marmol-Perez A	et al.	Co-morbid sarcopenia and low bone mineral density in young paediatric cancer survivors,	2024	10.1002/jcsm.13563	One or more keywords are missing
Marmol-Perez A	et al.	Risk of low bone mineral density in young pediatric cancer survivors with sarcopenia,	2024		Conference Abstract
Martín Giménez V	et al.	Melatonin as an Anti-Aging Therapy for Age-Related Cardiovascular and Neurodegenerative Diseases	2022	10.3389/fnagi.2022.888292	Review
Martín González C	et al.	Sclerostin in Excessive Drinkers: Relationships with Liver Function and Body Composition,	2022	10.3390/nu14132574	One or more keywords are missing
Martín González C	et al.	Serum Myostatin among Excessive Drinkers,	2023	10.3390/ijms24032981	One or more keywords are missing
Masiero S	et al.	Editorial: (Osteo)Sarcopenia & sarcopenic obesity	2023	10.3389/fendo.2023.1270350	One or more keywords are missing
Mathavan N	et al.	Assessing the use of the PolgA mouse model of premature aging to investigate the effects of aging on bone fracture healing,	2023	10.1002/jbm4.10738	Conference Abstract
Mathieu M	et al.	Association between bone mineral density and fat mass independent of lean mass and physical activity in women aged 75 or older,	2021	10.3390/nu13061994	One or more keywords are missing
Matos L	et al.	Osteosarcopenia in hip fracture patients,	2020	10.1016/j.clnesp.2020.09.712	Conference Abstract
Matsumoto M	et al.	Impact of Preoperative Osteosarcopenia and Postoperative Administration of Pancrelipase on the Prognosis of Borderline Resectable and Unresectable Locally	2025	10.1177/00031348241272420	One or more keywords are missing

		Advanced Pancreatic Cancer,			
Matsumoto M	et al.	Osteosarcopenia is a significant predictor of recurrence and the prognosis after resection for extrahepatic bile duct cancer,	2024	10.1007/s00595-023-02747-0	One or more keywords are missing
Maurer E	et al.	MRI-Based Phenotyping for Osteosarcopenic Adiposity in Subjects from a Population-Based Cohort	2024	10.3390/geriatrics9060150	One or more keywords are missing
Mazzola G	et al.	Bergamot (Citrus bergamia), a (Poly)Phenol-Rich Source for Improving Osteosarcopenic Obesity: A Systematic Review	2024	10.3390/foods13213422	One or more keywords are missing
McDonagh L	et al.	LEVELS OF PHYSICAL PERFORMANCE AND PHYSICAL ACTIVITY IN OLDER ATTENDEES AT A BONE HEALTH CLINIC,	2022	10.1093/ageing/afac218	Conference Abstract
McGregor R	et al.	Role of microRNAs in the age-related changes in skeletal muscle and diet or exercise interventions to promote healthy aging in humans	2014	10.1016/j.arr.2014.05.001	Review
Melouane A	et al.	Functional genomics applications and therapeutic implications in sarcopenia	2019	10.1016/j.mrrev.2019.04.003	Review
Messina C	et al.	Diagnostic imaging of osteoporosis and sarcopenia: A narrative review,	2018	10.21037/qims.2018.01.01	Review
Messina O	et al.	OBESITY, BONE METABOLISM, OSTEOPOROSIS AND FRACTURES,	2023	10.1007/s40520-023-02442-7	Conference Abstract
Miedany Y	et al.	Is there a potential dual effect of denosumab for treatment of osteoporosis and sarcopenia?,	2021	10.1007/s10067-021-05757-w	One or more keywords are missing

Mikovic J	et al.	MicroRNA and Long Non-coding RNA Regulation in Skeletal Muscle From Growth to Old Age Shows Striking Dysregulation of the Callipyge Locus	2018	10.3389/fgene.2018.00548	One or more keywords are missing
Mitchell C	et al.	Identification of human skeletal muscle miRNA related to strength by high-throughput sequencing	2018	10.1152/physiolgenomics.00112.2017	One or more keywords are missing
Miyagi M	et al.	Effect of osteosarcopenia on feeding status in hospitalized patients with suspected dysphagia,	2024	10.1371/journal.pone.0315091	One or more keywords are missing
Mogi M	et al.	Annual reports on hypertension research 2020	2022	10.1038/s41440-021-00766-3	Review
Molfino A	et al.	Novel therapeutic options for cachexia and sarcopenia	2016	10.1080/14712598.2016.1208168	Review
Mølmen K	et al.	Vitamin D3 supplementation does not enhance the effects of resistance training in older adults	2021	10.1002/jcsm.12688	One or more keywords are missing
Montalcini T	et al.	A call to action: Now is the time to screen elderly and treat osteosarcopenia, a position paper of the italian college of academic nutritionists med/49 (ican-49),	2020	10.3390/nu12092662	Review
Montano M	et al.	RNA surveillance-An emerging role for RNA regulatory networks in aging	2011	10.1016/j.arr.2010.02.002	Review
Montenegro J	et al.	Osteosarcopenia in patients with non-dialysis dependent chronic kidney disease,	2022	10.1016/j.clnu.2022.04.017	One or more keywords are missing
Moon K	et al.	Effects of osteo-sarcopenia on postoperative functional outcome and subsequent	2020	10.1016/j.bonr.2020.100528	Conference Abstract

		fracture in elderly hip fracture,			
Moreno-Aguilar M	et al.	Inverse association between body mass index and osteosarcopenia in community dwelling elderly,	2017	10.1016/j.soard.2017.09.419	Conference Abstract
Moretti A	et al.	Sclerostin: clinical insights in muscleâ€"bone crosstalk,	2023	10.1177/03000605231193293	Review
Moretti A	et al.	Osteosarcopenia and type 2 diabetes mellitus in post-menopausal women: a case-control study,	2022	10.52965/001c.38570	One or more keywords are missing
Morishita Y	et al.	Editorial: Frailty and Sarcopenia in Various Cachectic Kidney Diseases, Volume II	2022	10.3389/fmed.2022.936512	Editorial
Morishita Y	et al.	Editorial: Frailty and Sarcopenia in Various Cachectic Kidney Diseases	2021	10.3389/fmed.2020.627485	Editorial
Morley J	et al.	Pharmacologic Options for the Treatment of Sarcopenia	2016	10.1007/s00223-015-0022-5	Review
Morley J	et al.	The mTOR Conundrum: Essential for Muscle Function, but Dangerous for Survival	2016	10.1016/j.jamda.2016.09.001	Editorial
Moroni A	et al.	Discovering the Individualized Factors Associated with Sarcopenia and Sarcopenic Obesity Phenotypesâ€"A Machine Learning Approach,	2023	10.3390/nu15214536	One or more keywords are missing
Morozzi G	et al.	Oxidative stress-induced S100B accumulation converts myoblasts into brown adipocytes via an NF-? B/YY1/MIR-133 axis and NF-? B/YY1/BMP-7 axis	2017	10.1038/cdd.2017.132	One or more keywords are missing
Moser M	et al.	Correlation between MRI-based spinal muscle parameters and the vertebral bone quality	2023	10.1016/j.bas.2023.102684	One or more keywords are missing

		score in lumbar fusion patients,			
Moser M	et al.	Association of MRI-based spinal muscle parameters and vertebral bone quality score in lumbar fusion patients,	2022	10.1016/j.spinee.2022.06.222	Conference Abstract
Mueller M	et al.	Different molecular and structural adaptations with eccentric and conventional strength training in elderly men and women	2011	10.1159/000323267	One or more keywords are missing
Muellner M	et al.	The association between paraspinal muscle parameters and vertebral pedicle microstructure in patients undergoing lumbar fusion surgery,	2023	10.1007/s00264-022-05659-9	One or more keywords are missing
Murthy L	et al.	Higher Concentrations of Parathyroid Hormone (PTH) are Associated with Reduced Gait Velocity in Adults: A Systematic Review,	2022	10.1016/j.archger.2021.104579	Review
Musio A	et al.	Osteosarcopenia in NAFLD/MAFLD: An Underappreciated Clinical Problem in Chronic Liver Disease,	2023	10.3390/ijms24087517	Review
Nagendra L	et al.	Metabolic Bone Disease in the Tropics	2000		One or more keywords are missing
Naimi M	et al.	Comparative analysis of sarcopenia induced by long-term abiraterone (A) versus enzalutamide (E) therapy in men with metastatic castrationresistant prostate cancer (mCRPC),	2021	10.1200/JCO.2021.39.6_suppl.69	Conference Abstract
Nakano Y	et al.	Effect of osteosarcopenia on longitudinal mortality risk and chronic kidney disease progression in older adults,	2024	10.1016/j.bone.2023.116975	One or more keywords are missing

Nakano Y	et al.	Association Among Bone Mass, Muscle Mass and Strength, Mortality, and CKD Progression in Older Adults,	2023		Conference Abstract
Nazir S	et al.	Prevalence of Osteosarcopenia and Frailty in Patients with Chronic Liver Disease	2024	10.5005/jp-journals-10018-1442	One or more keywords are missing
Neshan M	et al.	Molecular Mechanisms of Cachexia: A Review	2024	10.3390/cells13030252	Review
Nguyen BN	et al.	Impacts of osteosarcopenia on musculoskeletal health, risks of falls and fractures, and activities of daily living among population aged 50 and above: an age- and sex-matched cross-sectional analysis,	2025	10.1007/s40520-024-02902-8	One or more keywords are missing
Nguyen BN	et al.	Dexamethasone-induced muscle atrophy and bone loss in six genetically diverse collaborative cross founder strains demonstrates phenotypic variability by Rg3 treatment,	2024	10.1016/j.jgr.2023.12.004	One or more keywords are missing
Nguyen M	et al.	Mir-302a/TWF1 Axis Impairs the Myogenic Differentiation of Progenitor Cells through F-Actin-Mediated YAP1 Activation	2023	10.3390/ijms24076341	One or more keywords are missing
Ni P	et al.	Indirect regulation of HIPPO pathway by miRNA mediates high-intensity intermittent exercise to ameliorate aging skeletal muscle function	2023	10.1111/sms.14338	One or more keywords are missing
Nie M	et al.	Noncoding RNAs, emerging regulators of skeletal muscle development and diseases	2015	10.1155/2015/676575	Review

Nielsen B	et al.	Sarcopenia and self-reported markers of physical frailty in patients with osteoporosis,	2024	10.1007/s11657-024-01437-9	One or more keywords are missing
Nielsen B	et al.	Prevalence of muscle dysfunction concomitant with osteoporosis in a home-dwelling Danish population aged 65â€“93 years - The Copenhagen Sarcopenia Study,	2020	10.1016/j.exger.2020.110974	One or more keywords are missing
Nielsen BR	et al.	Sarcopenia and self-reported markers of physical frailty in patients with osteoporosis	2024	10.1007/s11657-024-01437-9	One or more keywords are missing
Nielsen BR	et al.	Prevalence of muscle dysfunction concomitant with osteoporosis in a home-dwelling Danish population aged 65-93 years - The Copenhagen Sarcopenia Study	2020	10.1016/j.exger.2020.110974	One or more keywords are missing
O'Gara P	et al.	Osteosarcopenia and Mortality after Transcatheter Aortic Valve Replacement,	2024	10.1001/jamacardio.2024.1018	Editorial
Okamura H	et al.	Risk factors predicting osteosarcopenia in postmenopausal women with osteoporosis: A retrospective study,	2020	10.1371/journal.pone.0237454	One or more keywords are missing
Okayama A	et al.	Prevalence of Sarcopenia and Its Association with Quality of Life, Postural Stability, and Past Incidence of Falls in Postmenopausal Women with Osteoporosis: A Cross-Sectional Study	2022	10.3390/healthcare10020192	One or more keywords are missing
Okugawa Y	et al.	Circulating miR-203 derived from metastatic tissues promotes myopenia in colorectal cancer patients	2019	10.1002/jcsm.12403	One or more keywords are missing
Okugawa Y	et al.	Prognostic impact of sarcopenia and its correlation with	2018	10.3892/or.2018.6270	One or more keywords are missing

		circulating miR-21 in colorectal cancer patients			
Okun J	et al.	Liver alanine catabolism promotes skeletal muscle atrophy and hyperglycaemia in type 2 diabetes	2021	10.1038/s42255-021-00369-9	One or more keywords are missing
Okyar BA	et al.	Ultrasonografically assessed osteosarcopenic obesity is associated with frailty in community-dwelling older adults	2022	10.1016/j.nut.2022.111827	One or more keywords are missing
Okyar BA	et al.	Osteoporosis Is Associated With Low Muscle Mass Defined By Muscle Ultrasonography,	2023	10.1016/j.clnesp.2022.09.077	Conference Abstract
Omaña-Guzmán I	et al.	Undernutrition risk and obesity increase the risk of osteosarcopenia in Mexican adults aged 50 and over: a prospective cohort study	2025	10.3389/fnut.2024.1499453	One or more keywords are missing
Ortiz A	et al.	Sarcopenia in CKD: A roadmap from basic pathogenetic mechanisms to clinical trials,	2019	10.1093/ckj/sfz001	Review
Ostovar N	et al.	The association of dietary inflammatory index and osteosarcopenia in Iranian adults: results of iranian multicenter osteoporosis study,	2025	10.1007/s40200-025-01558-z	One or more keywords are missing
Ozer FF	et al.	Relation of bone mineral density with fat infiltration of paraspinal muscles: The Goutallier classification,	2024	10.1016/j.afos.2024.04.002	One or more keywords are missing
Paintin J	et al.	Osteosarcopenia	2018	10.12968/hmed.2018.79.5.253	One or more keywords are missing
Panahi G	et al.	The association between anti-diabetic agents and osteoporosis, sarcopenia, and osteosarcopenia among Iranian older adults Bushehr Elderly Health (BEH) program""	2023	10.1007/s40199-023-00497-5.	One or more keywords are missing

Pang BWJ	et al.	Coexistence of osteoporosis, sarcopenia and obesity in community-dwelling adults â€“ The Yishun Study,	2021	10.1016/j.afos.2020.12.002	One or more keywords are missing
Papadopetraki A	et al.	Physical Exercise Restrains Cancer Progression through Muscle-Derived Factors	2022	10.3390/cancers14081892	One or more keywords are missing
Papadopoulou S	et al.	Exercise and nutrition impact on osteoporosis and sarcopeniaâ€"the incidence of osteosarcopenia: A narrative review,	2021	10.3390/nu13124499	Review
Parikh K	et al.	Editorial: Insights in thoracic oncology: 2021/2022	2022	10.3389/fonc.2022.1089540	Editorial
Parinandi N	et al.	Antioxidants in longevity and medicine	2015	10.1155/2015/739417	Editorial
Park CH	et al.	Association between osteosarcopenia and coronary artery calcification in asymptomatic individuals,	2022	10.1038/s41598-021-02640-1	One or more keywords are missing
Park K	et al.	Disability, Frailty and Depression in the community-dwelling older adults with Osteosarcopenia,	2021	10.1186/s12877-021-02022-2	One or more keywords are missing
Park S	et al.	Circulating lumican as a potential biomarker for osteosarcopenia in older adults,	2024	10.1016/j.bone.2023.116959	One or more keywords are missing
Park S	et al.	Association between Disability and Edema Index Values in Rural Older Adult Osteosarcopenia Patients,	2022	10.3349/ymj.2022.63.9.873	One or more keywords are missing
Park S	et al.	Relationship between osteosarcopenic obesity and dietary inflammatory index in postmenopausal Korean women: 2009 to 2011 Korea National	2018	10.3164/jcbn.18-10	One or more keywords are missing

		Health and Nutrition Examination Surveys,			
Park SJ	et al.	Circulating lumican as a potential biomarker for osteosarcopenia in older adults	2024	10.1016/j.bone.2023.116959	One or more keywords are missing
Pasco J	et al.	Sarcopenia and estimates of fracture risk,	2017	10.1002/jbmr.3363	Conference Abstract
Pasco J	et al.	Musculoskeletal decline and mortality: prospective data from the Geelong Osteoporosis Study,	2017	10.1002/jcsm.12177	One or more keywords are missing
Patadia P	et al.	Anamorelin: Ghrelin Receptor Agonism as a Potential Intervention for Osteosarcopenia,	2024	10.1210/clinem/dgae043	Note
Paulin T	et al.	Osteosarcopenia: Prevalence and 10-Year Fracture and Mortality Risk â€" A Longitudinal, Population-Based Study of 75-Year-Old Women,	2024	10.1007/s00223-023-01181-1	One or more keywords are missing
Pechmann L	et al.	Osteosarcopenia and trabecular bone score in patients with type 2 diabetes mellitus,	2021	10.20945/2359-3997000000418	One or more keywords are missing
Penna F	et al.	Extracellular Vesicles and Exosomes in the Control of the Musculoskeletal Health	2024	10.1007/s11914-024-00866-2	One or more keywords are missing
Peppa M	et al.	Bioimpedance analysis vs. DEXA as a screening tool for osteosarcopenia in lean, overweight and obese caucasian postmenopausal females,	2017	10.14310/horm.2002.1732	One or more keywords are missing
Pérez-Baos S	et al.	Mediators and patterns of muscle loss in chronic systemic inflammation	2018	10.3389/fphys.2018.00409	Review
Peterson J	et al.	Editorial: MicroRNAs and Muscle Cell Death in Cancer	2022	10.3389/fgene.2022.892136	Editorial
Petrella G	et al.	Urinary metabolic markers of bladder cancer: A reflection of the	2021	10.3390/metabo11110756	Review

		tumor or the response of the body?			
Phu S	et al.	Effect of Denosumab on Falls, Muscle Strength, and Function in Community-Dwelling Older Adults,	2019	10.1111/jgs.16165	Letter
Pivtorak K	et al.	RELATIONSHIP BETWEEN SARCOPENIA AND OSTEOPOROSIS IN NON-ALCOHOLIC FATTY LIVER DISEASE,	2022		One or more keywords are missing
Pizzonia M	et al.	Osteosarcopenia in Very Old Age Adults After Hip Fracture: A Real-World Therapeutic Standpoint,	2021	10.3389/fmed.2021.612506	One or more keywords are missing
Poggiogalle E	et al.	Body Composition, IGF1 Status, and Physical Functionality in Nonagenarians: Implications for Osteosarcopenia,	2019	10.1016/j.jamda.2018.07.007	One or more keywords are missing
Polito A	et al.	Osteosarcopenia: A Narrative Review on Clinical Studies,	2022	10.3390/ijms23105591	Review
Poon C	et al.	Traditional Chinese medicine (TCM) formula Er Zhi Wan (EZW) ameliorates aging-induced muscle and bone loss in aging osteosarcopenic animal model,	2024		Conference Abstract
Poon C	et al.	Kidney-tonifying Chinese herbal medicine Fructus Ligustri Lucidi (FLL) promotes myogenesis in C2C12 myoblasts via increasing vitamin D sensitivity,	2023	10.1186/s13020-023-00796-8	Conference Abstract
Pourhassan M	et al.	Three-Year Mortality of Older Hospitalized Patients with Osteosarcopenia: Data from the OsteoSys Study,	2024	10.3390/nu16091328	One or more keywords are missing

Pourhassan M	et al.	Osteosarcopenia, an asymmetrical overlap of two connected syndromes: Data from the osteosys study,	2021	10.3390/nu13113786	One or more keywords are missing
Pourhassan M	et al.	Three-Year Mortality of Older Hospitalized Patients with Osteosarcopenia: Data from the OsteoSys Study	2024	10.3390/nu16091328	One or more keywords are missing
Proctor C	et al.	Using computer simulation models to investigate the most promising microRNAs to improve muscle regeneration during ageing	2017	10.1038/s41598-017-12538-6	One or more keywords are missing
Pugliese N	et al.	Osteosarcopenia in autoimmune cholestatic liver diseases: Causes, management, and challenges,	2022	10.3748/wjg.v28.i14.1430	Review
Qian L	et al.	Peroxisome proliferator-activated receptor gamma coactivator-1 (PGC-1) family in physiological and pathophysiological process and diseases	2024	10.1038/s41392-024-01756-w	Review
Qiu C	et al.	Spatial Transcriptome Crosstalk for Bone & Muscle in Mouse Femur	2024	10.1093/jbmr/zjad017	Conference Abstract
Qiu D	et al.	Muscle-enriched microRNA-486-mediated regulation of muscular atrophy and exercise	2024	10.1007/s13105-024-01043-w	Review
Radkowski M	et al.	Osteosarcopenia in rheumatoid arthritis treated with glucocorticosteroids - Essence, significance, consequences	2020	10.5114/reum.2020.95364	Review
Rahman F	et al.	Mitochondrial Apoptotic Signaling Involvement in Remodeling During Myogenesis and Skeletal Muscle Atrophy	2023	10.1016/j.semcdb.2022.01.011	Review
Raleigh S	et al.	Sarcopenia as a Risk Factor for Alzheimer's	2024	10.3390/genes15050561	Review

		Disease: Genetic and Epigenetic Perspectives			
Rashid A	et al.	Low Muscle Mass Is Associated with Low Bone Mineral Density in Patients with CKD Stages G4-5,	2024		Conference Abstract
Raskina T	et al.	Sarcopenic syndrome in patients with coronary heart disease,	2020	10.1007/s00198-020-05696-3	Conference Abstract
Raskina T	et al.	Frequency and clinical characteristics of osteosarcopenia in patients with ischemic heart disease,	2019	10.1007/s00198-019-04993-w	Conference Abstract
Reddy P	et al.	Preface	2017	10.1016/S1877-1173(17)30028-5	Editorial
Reiss J	et al.	Sarkopenie und Osteoporose sind bei geriatrischen Krankenhauspatienten miteinander assoziiert,	2019	10.1007/s00391-019-01553-z	One or more keywords are missing
Rivas D	et al.	Sphingosine-1-phosphate analog FTY720 reverses obesity but not age-induced anabolic resistance to muscle contraction	2019	10.1152/ajpcell.00455.2018	One or more keywords are missing
Rivas D	et al.	Diminished skeletal muscle microRNA expression with aging is associated with attenuated muscle plasticity and inhibition of IGF-1 signaling	2014	10.1096/fj.14-254490	One or more keywords are missing
Rivero-Segura N	et al.	Promising biomarkers of human aging: In search of a multi-omics panel to understand the aging process from a multidimensional perspective	2020	10.1016/j.arr.2020.101164	Review
Rosas-Carrasco O	et al.	Osteosarcopenia predicts greater risk of functional disability than sarcopenia: a longitudinal analysis of FraDySMex cohort study,	2024	10.1016/j.jnha.2024.100368	One or more keywords are missing

Ruan L	et al.	Long Non-coding RNA MALAT1 Is Depleted With Age in Skeletal Muscle in vivo and MALAT1 Silencing Increases Expression of TGF-?1 in vitro	2022	10.3389/fphys.2021.742004	One or more keywords are missing
Rubek Nielsen B	et al.	Osteosarcopenia: Prevalence and consequences,	2022	10.1007/s00198-021-06117-9	Conference Abstract
Rupp T	et al.	Beneficial effects of denosumab on muscle performance in patients with low BMD: a retrospective, propensity score-matched study,	2022	10.1007/s00198-022-06470-3	One or more keywords are missing
Rusanova I	et al.	Analysis of Plasma MicroRNAs as Predictors and Biomarkers of Aging and Frailty in Humans	2018	10.1155/2018/7671850	One or more keywords are missing
Rusanova I	et al.	Involvement of plasma miRNAs, muscle miRNAs and mitochondrial miRNAs in the pathophysiology of frailty	2019	10.1016/j.exger.2019.110637	Review
Saeki C	et al.	Association of chronic liver disease with bone diseases and muscle weakness,	2024	10.1007/s00774-023-01488-x	Review
Saeki C	et al.	Osteosarcopenia predicts poor survival in patients with cirrhosis: a retrospective study,	2023	10.1186/s12876-023-02835-y	One or more keywords are missing
Saeki C	et al.	Relationship between osteoporosis, sarcopenia, vertebral fracture, and osteosarcopenia in patients with primary biliary cholangitis,	2021	10.1097/MEG.0000000000001791	One or more keywords are missing
Saeki C	et al.	Comparative assessment of sarcopenia using the JSH, AWGS, and EWGSOP2 criteria and the relationship between sarcopenia, osteoporosis, and osteosarcopenia in patients with liver cirrhosis,	2019	10.1186/s12891-019-2983-4	One or more keywords are missing

Saeki C	et al.	Influencing Factors and Molecular Pathogenesis of Sarcopenia and Osteosarcopenia in Chronic Liver Disease	2021	10.3390/life11090899	One or more keywords are missing
Saeki C	et al.	Relationship between Osteosarcopenia and Frailty in Patients with Chronic Liver Disease	2020	10.3390/jcm9082381	One or more keywords are missing
Saeki C	et al.	Osteosarcopenia predicts poor survival in patients with cirrhosis: a retrospective study	2023	10.1186/s12876-023-02835-y	One or more keywords are missing
Safer V	et al.	Ultrasonographically Measured Rectus Femoris Cross-sectional Area might Predict Osteosarcopenia,	2024	10.2174/011573405627513323121115630	One or more keywords are missing
Said Noor M	et al.	A case study implementing a strength training programme with the aim to reduce the risk of osteosarcopenia and bone fractures,	2021	10.1093/bjs/znab259.396	Conference Abstract
Salamanna F	et al.	A Pilot Study on Circulating, Cellular, and Tissue Biomarkers in Osteosarcopenic Patients,	2024	10.3390/ijms25115879	One or more keywords are missing
Salamanna F	et al.	Sharing Circulating Micro-RNAs between Osteoporosis and Sarcopenia: A Systematic Review	2023	10.3390/life13030602	Review
Salech F	et al.	Osteosarcopenia Predicts Falls, Fractures, and Mortality in Chilean Community-Dwelling Older Adults,	2021	10.1016/j.jamda.2020.07.032	One or more keywords are missing
Salvadori L	et al.	Equisetum arvense standardized dried extract hinders age-related osteosarcopenia,	2024	10.1016/j.biopha.2024.116517	One or more keywords are missing
Sannicandro A	et al.	Micro(RNA)-managing muscle wasting	2019	10.1152/japplphysiol.00961.2018	Review
Santos H	et al.	The Effects of Dietary Supplements, Nutraceutical Agents, and	2022	10.3390/metabo12111146	Review

		Physical Exercise on Myostatin Levels: Hope or Hype?			
Sárközy M	et al.	Mechanisms and Modulation of Oxidative/Nitrative Stress in Type 4 Cardio-Renal Syndrome and Renal Sarcopenia	2018	10.3389/fphys.2018.01648	Review
Sasaki K	et al.	Sarcopenia as a comorbidity of cardiovascular disease,	2022	10.1016/j.jjcc.2021.10.013	Review
Sasaki K	et al.	Author's reply,	2020	10.1016/j.jjcc.2020.06.012	Letter
Sasaki K	et al.	The prevalence of sarcopenia and subtypes in cardiovascular diseases, and a new diagnostic approach,	2020	10.1016/j.jjcc.2020.03.004	One or more keywords are missing
Sasako T	et al.	Deletion of skeletal muscle Akt1/2 causes osteosarcopenia and reduces lifespan in mice,	2022	10.1038/s41467-022-33008-2	One or more keywords are missing
Sato R	et al.	Sarcopenia and Frailty in Heart Failure: Is There a Biomarker Signature?	2022	10.1007/s11897-022-00575-w	Review
Saul D	et al.	Epigenetics of aging and aging-associated diseases	2021	10.3390/ijms22010401	Review
Scheuren A	et al.	Hallmarks of frailty and osteosarcopenia in prematurely aged PolgA((D257A/D257A)) mice,	2020	10.1002/jcsm.12588	One or more keywords are missing
Scheuren A	et al.	Longitudinal evaluation of the musculoskeletal and frailty phenotype in a mouse model of accelerated aging,	2019	10.1007/s00223-019-00544-x	Conference Abstract
Schmidmaier R	et al.	Osteosarcopenia is more than sarcopenia and osteopenia alone,	2016		Conference Abstract
Schröder G	et al.	Evaluierung von Knochendichte und Handgriffkraft im Verlauf einer medikamentÃ¶sen Osteoporosetherapie : Eine Real-World-Studie,	2023	10.1007/s00132-023-04367-5	One or more keywords are missing

Schröder G	et al.	[Impact of osteoporosis on physical performance parameters of middle-aged and elderly individuals-a cross-sectional study]	2023	10.1007/s00132-022-04329-3	One or more keywords are missing
Scott D	et al.	‘Giant’ Claims Require Strong Evidence: A Comment on ‘Osteosarcopenia: A Geriatric Giant of the XXI Century’	2021	10.1007/s12603-021-1659-6	Letter
Scott D	et al.	Comment on: Osteosarcopenia: where osteoporosis and sarcopenia collide,	2021	10.1093/rheumatology/keab066	Letter
Scott D	et al.	Does Combined Osteopenia/Osteoporosis and Sarcopenia Confer Greater Risk of Falls and Fracture Than Either Condition Alone in Older Men? The Concord Health and Ageing in Men Project,	2019	10.1093/gerona/gly162	One or more keywords are missing
Scott D	et al.	Letter to the Editor: 'Giant' Claims Require Strong Evidence: A Comment on 'Osteosarcopenia: A Geriatric Giant of the XXI Century'	2021	10.1007/s12603-021-1659-6	One or more keywords are missing
Seaton M	et al.	Associations of Lean Mass, Muscular Strength, and Physical Function with Trabecular Bone Score in Older Adults,	2023	10.1016/j.jocd.2023.101370	One or more keywords are missing
Seco-Cervera M	et al.	Circulating miR-28-5p is overexpressed in patients with sarcopenia despite long-term remission of Cushing’s syndrome: a pilot study	2024	10.3389/fendo.2024.1410080	One or more keywords are missing
Seldeen K	et al.	Chronic vitamin D insufficiency impairs physical performance in C57BL/6J mice	2018	10.18632/aging.101471	One or more keywords are missing
Sellami M	et al.	Regular, Intense Exercise Training as a Healthy	2021	10.3389/fgene.2021.652497	Review

		Aging Lifestyle Strategy: Preventing DNA Damage, Telomere Shortening and Adverse DNA Methylation Changes Over a Lifetime			
Sepúlveda-Loyola W	et al.	The Joint Occurrence of Osteoporosis and Sarcopenia (Osteosarcopenia): Definitions and Characteristics	2020	10.1016/j.jamda.2019.09.005	One or more keywords are missing
Sepúlveda-Loyola W	et al.	Clinical implications of osteosarcopenia and its components in community-dwelling older adults	2019		Conference Abstract
Serra V	et al.	A possible role for essential amino acid supplementation in improving functional recovery and reducing the risk for malnutrition in COVID-19 patients,	2024	10.23751/pn.v26i3-4.15859	One or more keywords are missing
Shafiee G	et al.	The impact of osteosarcopenia and its parameters on mortality of COVID-19 in-hospitalized older patients: the findings of BEH (Bushehr elderly health) program,	2024	10.1007/s40200-024-01443-1	One or more keywords are missing
Shafiee G	et al.	Overlap between Osteosarcopenia and Frailty and their Association with Poor Health Conditions: The Bushehr Elderly Health Program,	2024	10.4235/agmr.23.0220	One or more keywords are missing
Shafiee G	et al.	THE RELATIONSHIP BETWEEN OSTEOSARCOPENIA AND COGNITIVE FRAILTY AMONG OLDER PEOPLE: THE BUSHEHR ELDERLY HEALTH (BEH) PROGRAM,	2022	10.1007/s40520-022-02147-3	Conference Abstract

Shafiee G	et al.	Nutrition status and inflammatorypotentialof the diet and risk of osteosarcopenia: The bushehr elderly health (BEH) program,	2022	10.1007/s00198-021-06125-9	Conference Abstract
Shafiee G	et al.	Osteosarcopenia and biomarkers of bone health in Iranian older people: The bushehr elderly health (BEH) program,	2022	10.1007/s00198-021-06125-9	Conference Abstract
Shafiee G	et al.	Association between osteosarcopenia and depression in older people: The bushehr elderly health (BEH) program,	2020	10.1007/s00198-020-05696-3	Conference Abstract
Shafiee G	et al.	The impact of osteosarcopenia and its parameters on mortality of COVID-19 in-hospitalized older patients: the findings of BEH (Bushehr elderly health) program	2024	10.1007/s40200-024-01443-1	One or more keywords are missing
Shao X	et al.	Atrophic skeletal muscle fibre-derived small extracellular vesicle miR-690 inhibits satellite cell differentiation during ageing	2022	10.1002/jcsm.13106	One or more keywords are missing
Sharma A	et al.	Targeting Crosstalk of Signaling Pathways among Muscles-Bone-Adipose Tissue: A Promising Therapeutic Approach for Sarcopenia	2024	10.14336/AD.2023.00903	Review
Sharma M	et al.	Mega roles of microRNAs in regulation of skeletal muscle health and disease	2014	10.3389/fphys.2014.00239	Review
Sheng R	et al.	Muscle-bone crosstalk via endocrine signals and potential targets for osteosarcopenia-related fracture,	2023	10.1016/j.jot.2023.09.007	Review
Shepherd J	et al.	3D optical scans for assessment sarcopenia, obesity, and osteopenia,	2017	10.1007/s00198-017-3950-2	Conference Abstract

Shiba T	et al.	Features of older community-dwelling adults with osteosarcopenia requiring support or care	2022	10.1589/jpts.34.341	One or more keywords are missing
Shimada H	et al.	Impact of osteosarcopenia on disability and mortality among Japanese older adults,	2023	10.1002/jcsm.13209	One or more keywords are missing
Shimagaki T	et al.	Prognostic impact of osteosarcopenia on postoperative outcomes in patients with biliary tract cancer,	2024	10.1007/s00595-024-02972-1	One or more keywords are missing
Shin D	et al.	Exosomes Secreted During Myogenic Differentiation of Human Fetal Cartilage-Derived Progenitor Cells Promote Skeletal Muscle Regeneration through miR-145-5p	2024	10.1007/s13770-023-00618-w	One or more keywords are missing
Shin H	et al.	Circulating small non-coding RNA profiling for identification of older adults with low muscle strength and physical performance: A preliminary study	2024	10.1016/j.exger.2024.112598	One or more keywords are missing
Shin H	et al.	MicroRNAs as commonly expressed biomarkers for sarcopenia and frailty: A systematic review	2024	10.1016/j.exger.2024.112600	Review
Shin Y	et al.	The role of non-coding RNAs in muscle aging: regulatory mechanisms and therapeutic potential	2023	10.3389/fmolb.2023.1308274	Review
Shin Y	et al.	A subset of microRNAs in the Dlk1-Dio3 cluster regulates age-associated muscle atrophy by targeting Atrogin-1	2020	10.1002/jcsm.12578	One or more keywords are missing
Shorter E	et al.	Skeletal Muscle Wasting and Its Relationship With Osteoarthritis: a Mini-Review of Mechanisms and Current Interventions	2019	10.1007/s11926-019-0839-4	Review

Shrestha A	et al.	Acute Sarcopenia after Elective and Emergency Surgery	2022	10.14336/AD.2022.0404	Review
Silva WJ	et al.	MicroRNAs and their Modulatory Effect on the Hallmarks of Osteosarcopenia	2024	10.1007/s11914-024-00880-4	Review
Silveira EA	et al.	Osteosarcopenia later in life: Prevalence and associated risk factors,	2023	10.1016/j.clnesp.2023.08.030	One or more keywords are missing
Simon A	et al.	Compartment-specific effects of muscle strength on bone microarchitecture in women at high risk of osteoporosis,	2022	10.1002/jcsm.13044	One or more keywords are missing
Singh G	et al.	Tiny Regulators of Massive Tissue: MicroRNAs in Skeletal Muscle Development, Myopathies, and Cancer Cachexia	2020	10.3389/fonc.2020.598964	Review
Skrzypczak D	et al.	A vicious cycle of osteosarcopeniain inflammatory bowel diseasesâ€”aetiology, clinical implications and therapeutic perspectives,	2021	10.3390/nu13020293	Review
Smith C	et al.	The Interconnection Between Muscle and Bone: A Common Clinical Management Pathway,	2024	10.1007/s00223-023-01146-4	Review
Smith C	et al.	Letter to the editor: Osteosarcopenia in reproductive-aged women with polycystic ovary syndrome: A multicenter case-control study,	2021	10.1210/clinem/dgaa669	Letter
Soares D	et al.	P-8 OSTEOSARCOPENIA AND FIBROSIS SEVERITY IN NON-ALCOHOLIC FATTY LIVER DISEASE,	2024	10.1016/j.aohep.2023.101195	Conference Abstract
Sohi Y	et al.	The association between anti-diabetic agents and osteoporosis, sarcopenia, and osteosarcopenia among Iranian older	2024	10.1007/s40199-023-00497-5	One or more keywords are missing

		adults; Bushehr Elderly Health (BEH) program,			
Solla-Suarez P	et al.	Osteosarcopenia and Mortality in Older Adults Undergoing Transcatheter Aortic Valve Replacement,	2024	10.1001/jamacardio.2024.0911	One or more keywords are missing
Sorci G	et al.	Report and Abstracts of the 17th Meeting of IIM, the Interuniversity Institute of Myology: Virtual meeting, October 16-18, 2020	2021	10.4081/ejtm.2020.9485	One or more keywords are missing
Soriano-Arroquia A	et al.	Age-related changes in miR-143-3p: Igfbp5 interactions affect muscle regeneration	2016	10.1111/acel.12442	One or more keywords are missing
Soriano-Arroquia A	et al.	The functional consequences of age-related changes in microRNA expression in skeletal muscle	2016	10.1007/s10522-016-9638-8	One or more keywords are missing
Sorokina A	et al.	PATHOLOGICAL PHENOTYPES OF BODY COMPOSITION IN PATIENTS WITH RHEUMATIC DISEASES,	2022	10.47360/1995-4484-2022-487-494	One or more keywords are missing
Stangl M	et al.	Sarcopenia - Endocrinological and Neurological Aspects	2019	10.1055/a-0672-1007	Review
Stefanaki C	et al.	Lean women on metformin and oral contraceptives for polycystic ovary syndrome demonstrate a dehydrated osteosarcopenic phenotype: A pilot study,	2019	10.3390/nu11092055	One or more keywords are missing
Stefanaki C	et al.	The impact of probioticsâ€™ administration on glycemic control, body composition, gut microbiome, mitochondria, and other hormonal signals in adolescents with prediabetes â€“ A	2018	10.1016/j.conctc.2018.06.002	One or more keywords are missing

		randomized, controlled trial study protocol,			
Stefanaki C	et al.	Chronic stress and body composition disorders: implications for health and disease,	2018	10.1007/s42000-018-0023-7	Review
Stefanaki C	et al.	Healthy overweight/obese youth: early osteosarcopenic obesity features,	2016	10.1111/eci.12659	One or more keywords are missing
Strasser B	et al.	Importance of Assessing Muscular Fitness in Secondary Care	2020	10.3389/fgene.2020.583810	One or more keywords are missing
Su YH	et al.	A study of correlations between metabolic syndrome factors and osteosarcopenic adiposity,	2021	10.1186/s12902-021-00880-w	One or more keywords are missing
Suleymanova A	et al.	Main osteosarcopenia risk factors in stable COPD patients,	2022	10.1007/s00198-021-06125-9	Conference Abstract
Suleymanova A	et al.	Osteosarcopenia and severe osteosarcopenia in COPD patients,	2020	10.1007/s00198-020-05696-3	Conference Abstract
Suleymanova A	et al.	The main predictors and consequences of osteosarcopenia in COPD patients,	2020	10.1183/13993003.congress-2020.2985	Conference Abstract
Suleymanova A	et al.	Musculoskeletal disorders in patients with chronic obstructive pulmonary disease,	2019	10.18093/0869-0189-2019-29-1-94-105	One or more keywords are missing
Sun C	et al.	Able & stable-get more stable, be more able: A review of a falls prevention programme, a first step towards frailty and osteosarcopenia prevention,	2018	10.1007/s00198-018-4465-1	Conference Abstract
Suriyaarachchi P	et al.	High parathyroid hormone levels are associated with osteosarcopenia in older individuals with a history of falling,	2018	10.1016/j.maturitas.2018.04.006	One or more keywords are missing

Suriyaarachchi P	et al.	Hyperparathyroidism is associated with osteosarcopenia in older individuals with a history of falling,	2015	10.1002/jbmr.2763	Conference Abstract
Suzuki T	et al.	MicroRNAs in muscle wasting	2018	10.1002/jcsm.12384	One or more keywords are missing
Swan M	et al.	Meeting report: American Aging Association 40th Annual Meeting, Raleigh, North Carolina, June 3-6, 2011	2011	10.1089/rej.2011.1216	Conference Pa[illegible]
Tabatabaei-Malazy O	et al.	Editorial: Community series - reducing the burden of age-related disease in relation to osteoporosis, sarcopenia and osteosarcopenia, volume II,	2023	10.3389/fmed.2023.1344694	Editorial
Tabatabaei-Malazy O	et al.	Editorial: Reducing the Burden of Age-Related Disease in Relation to Osteoporosis, Sarcopenia and Osteosarcopenia,	2022	10.3389/fmed.2022.882140	Editorial
Tada M	et al.	Osteosarcopenia synergistically increases the risk of falls in patients with rheumatoid arthritis,	2021	10.1016/j.afos.2021.11.002	One or more keywords are missing
Tada M	et al.	Osteosarcopenia increases the risk of falls in patients with rheumatoid arthritis: Results of a four-year longitudinal study,	2021	10.1136/annrheumdis-2021-eular.942	Conference Abstract
Takada Y	et al.	Tumor Necrosis Factor-? Blunts the Osteogenic Effects of Muscle Cell-Derived Extracellular Vesicles by Affecting Muscle Cells	2023	10.1007/s00223-022-01056-x	One or more keywords are missing
Takano Y	et al.	Prognostic significance of osteosarcopenia in older adults with colorectal cancer,	2023	10.1002/ags3.12663	One or more keywords are missing
Takeda T	et al.	The impact of osteosarcopenia in patients with unresectable or recurrent biliary tract	2023	10.1093/jjco/hyad097	One or more keywords are missing

		cancer receiving palliative chemotherapy,			
Takeda T	et al.	Prognostic impact of osteosarcopenia in patients with advanced pancreatic cancer receiving gemcitabine plus nab-paclitaxel: Osteosarcopenia in pancreatic cancer,	2023	10.1016/j.pan.2023.02.002	One or more keywords are missing
Tamura Y	et al.	Comparison of the ability between dual-energy X-ray absorptiometry and bioelectrical impedance analysis for diagnosing low skeletal muscle mass and sarcopenia in patients with chronic liver disease,	2025	10.1111/jgh.16806	One or more keywords are missing
Tan L	et al.	Molecular genetic studies of gene identification for sarcopenia	2012	10.1007/s00439-011-1040-7	Review
Tan Y	et al.	Osteoporosis in Parkinson's Disease: Relevance of Distal Radius Dual-Energy X-Ray Absorptiometry (DXA) and Sarcopenia,	2021	10.1016/j.jocd.2020.07.001	One or more keywords are missing
Tang H	et al.	The association between computed tomography-based osteosarcopenia and osteoporotic vertebral fractures: a longitudinal study,	2025	10.1007/s40618-024-02415-1	One or more keywords are missing
Tang M	et al.	Botulinum Toxin A and Osteosarcopenia in Experimental Animals: A Scoping Review,	2021	10.3390/TOXINS13030213	Review
Tang S	et al.	Effects of soy foods in postmenopausal women: A focus on osteosarcopenia and obesity,	2021	10.7570/jomes20006	Review
Taniai T	et al.	OSTEOSARCOPENIA IS ASSOCIATED WITH POOR ONCOLOGIC OUTCOMES IN PATIENTS WITH INTRAHEPATIC CHOLANGIOCARCINO	2023	10.1097/HEP.0000000000000580	Conference Abstract

		MA AFTER HEPATIC RESECTION,			
Taniai T	et al.	Osteosarcopenia predicts poor prognosis for patients with intrahepatic cholangiocarcinoma after hepatic resection,	2023	10.1007/s00595-022-02550-3	One or more keywords are missing
Taniguchi Y	et al.	Associations of the Alpha-Actinin Three Genotype with Bone and Muscle Mass Loss among Middle-Aged and Older Adults,	2022	10.3390/jcm11206172	One or more keywords are missing
Tarantino U	et al.	Osteosarcopenia and Long-COVID: a dangerous combination,	2022	10.1177/1759720X221130485	Review
Tarantino U	et al.	Sarcopenia and bone health: new acquisitions for a firm liaison,	2022	10.1177/1759720X221138354	Review
Tarantino U	et al.	T-score and handgrip strength association for the diagnosis of osteosarcopenia: A systematic review and meta-analysis,	2021	10.3390/jcm10122597	Review
Tegola L	et al.	Diagnostic imaging of two related chronic diseases: Sarcopenia and Osteoporosis	2018	10.22540/JFSF-03-138	One or more keywords are missing
Teixeira L	et al.	Inflammatory biomarkers of osteosarcopenia in community-dwelling older woman,	2024	10.1016/j.nutos.2024.03.014	One or more keywords are missing
Tembo MC	et al.	The contribution of musculoskeletal factors to physical frailty: a cross-sectional study,	2021	10.1186/s12891-021-04795-4	One or more keywords are missing
Teng Z	et al.	The analysis of osteosarcopenia as a risk factor for fractures, mortality, and falls,	2021	10.1007/s00198-021-05963-x	One or more keywords are missing
Terentyeva N	et al.	The structure of complications in patients with different musculoskeletal disorders after coronary artery bypass surgery,	2022	10.20333/25000136-2022-4-54-60	One or more keywords are missing

Terentyeva N	et al.	RISK FACTORS FOR UNFAVORABLE PROGNOSIS IN PATIENTS WITH CORONARY ARTERY DISEASE AND AGE-RELATED DISORDERS UNDERGOING CORONARY ARTERY BYPASS GRAFTING,	2022	10.17802/2306-1278-2022-11-4-13-24	One or more keywords are missing
Terentyeva N	et al.	Impact of musculoskeletal conditions on the perioperative period in patients with stable coronary artery disease,	2021	10.1093/eurheartj/ehab724.1233	Conference Abstract
Thomson M	et al.	Letter: Prioritising osteosarcopenia assessment in the ongoing care of patients with cholestatic liver diseases. Authorsâ€™ reply,	2024	10.1111/apt.17877	Letter
Toroptsova N	et al.	Body composition in patients with rheumatoid arthritis,	2020	10.1136/annrheumdis-2020-eular.2318	Conference Abstract
Trajanoska K	et al.	Clinical correlates and prevalence of sarcopenia and osteosarcopenia in a population based cohort,	2017	10.1007/s00223-017-0267-2	Conference Abstract
Trajanoska K	et al.	Prevalence of sarcopenia and osteosarcopenia,	2017	10.1002/jbmr.3107	Conference Abstract
Trottier M	et al.	Preoperative optimization: Physical and cognitive pre-habilitation and management of chronic medication,	2023	10.4103/sja.sja_583_23	One or more keywords are missing
Trovato E	et al.	Extracellular vesicles: Delivery vehicles of myokines	2019	10.3389/fphys.2019.00522	Review
Tsai C	et al.	Endothelin-1-mediated miR-let-7g-5p triggers interleukin-6 and TNF? to cause myopathy and chronic adipose inflammation in elderly patients with diabetes mellitus	2022	10.18632/aging.204034	One or more keywords are missing

Tsekoura M	et al.	ÎŸsteosarcopenia A brief overview of the disease of the future,	2020		One or more keywords are missing
Tufail E	et al.	The role of glucagon-like peptides in osteosarcopenia,	2025	10.1530/JOE-24-0210	Review
Tuna M	et al.	Evaluation of Osteosarcopenia and Biochemical Parameters in People Living with HIV,	2023	10.5578/flora.20239608	One or more keywords are missing
Turkmen I	et al.	Osteosarcopenia increases hip fracture risk: A case-controlled study in the elderly,	2019	10.3233/BMR-181389	One or more keywords are missing
Uemura K	et al.	Assessing the utility of osteoporosis self-assessment tool for Asians in patients undergoing hip surgery,	2024	10.1016/j.afos.2024.01.003	One or more keywords are missing
Vahdani A	et al.	Assessment of quality of life and its affecting factors in osteosarcopenic individuals in the Iranian older adult population: Bushehr Elderly Health (BEH) program,	2023	10.1016/j.afos.2023.12.002	One or more keywords are missing
Vainshtein A	et al.	Signaling pathways that control muscle mass	2020	10.3390/ijms21134759	Review
Vaishya R	et al.	Hand grip strength as a proposed new vital sign of health: a narrative review of evidences,	2024	10.1186/s41043-024-00500-y	Review
Valášková S	et al.	The Severity of Muscle Performance Deterioration in Sarcopenia Correlates With Circulating Muscle Tissue-Specific miRNAs	2021	10.33549/PHYSIOLRES.934778	One or more keywords are missing
Valente A	et al.	Birth weight associated with dual energy X-ray absorptiometry-determined muscle-bone unit in young healthy women from the Nutritionists' Health Study,	2021	10.1017/S2040174419000874	One or more keywords are missing

Valiño-Rivas L et al.	NIK as a Druggable Mediator of Tissue Injury,	2019	10.1016/j.molmed.2019.02.005	Review
Vanderschueren D et al.	Bone disorders: Mechanisms and targets	2016	10.1016/j.mce.2016.06.001	Editorial
Veronese N et al.	Osteosarcopenia increases the risk of mortality: a systematic review and meta-analysis of prospective observational studies,	2024	10.1007/s40520-024-02785-9	Review
Veronesi F et al.	Unlocking diagnosis of sarcopenia: The role of circulating biomarkers – A clinical systematic review	2024	10.1016/j.mad.2024.112005	One or more keywords are missing
Vinícius-Souza GE et al.	Effectiveness of exercise for osteosarcopenia in older adults: A systematic review protocol,	2021	10.1136/bmjopen-2020-045604	Review
Vlak T et al.	Relationship of body composition with osteoporosis,	2020	10.1007/s00198-020-05695-4	Conference Abstract
Vlietstra L et al.	Using minimal clinically important differences to measure long-term transitions of osteosarcopenia: The New Mexico Aging Process Study,	2023	10.1016/j.exger.2023.112106	Review
Vogrin S et al.	Clinical Utility of Thigh and Mid-Thigh Dual-Energy x-Ray Absorptiometry to Identify Bone and Muscle Loss,	2023	10.1002/jbm4.10704	One or more keywords are missing
Vrbová P et al.	Biomarkers of the Physical Function Mobility Domains Among Patients Hospitalized in Internal Medicine	2021	10.33549/PHYSIOLRES.934777	One or more keywords are missing
Vucic V et al.	Nutrition and Physical Activity as Modulators of Osteosarcopenic Adiposity: A Scoping Review and Recommendations for Future Research,	2023	10.3390/nu15071619	Review

Wang A	et al.	Age and low intensity resistance exercise on exosome-like vesicle and skeletal muscle microRNA profiles	2023	10.1113/JP284547	Note
Wang CC	et al.	Osteosarcopenia in patients with cancer: A systematic review and meta-analysis,	2024	10.1097/MD.0000000000040476	Review
Wang K	et al.	Chronic kidney disease-induced muscle atrophy: Molecular mechanisms and promising therapies	2023	10.1016/j.bcp.2022.115407	Review
Wang L	et al.	Investigating the Causal Effects of Exercise-Induced Genes on Sarcopenia	2024	10.3390/ijms251910773	One or more keywords are missing
Wang L	et al.	Factors inducing transdifferentiation of myoblasts into adipocytes	2021	10.1002/jcp.30074	Review
Wang Q	et al.	Serum AMPK-? mRNA, SIRT1 and GDF-8 levels in patients with sarcopenia	2022	10.16766/j.cnki.issn.1674-4152.002545	One or more keywords are missing
Wang R	et al.	Computed tomography (CT)-based osteosarcopenia evaluation during chest CT scans,	2025	10.1007/s40618-024-02433-z	Letter
Wang R	et al.	The association between HDL-c levels and computed tomography-based osteosarcopenia in older adults,	2024	10.1186/s12891-024-08059-9	One or more keywords are missing
Wang S	et al.	A Novel MRI-Based Paravertebral Muscle Quality (PVMQ) Score for Evaluating Muscle Quality and Bone Quality: A Comparative Study with the VBQ Score,	2024	10.2147/CIA.S464187	One or more keywords are missing
Wang W	et al.	Biogenesis and function of extracellular vesicles in pathophysiological processes of skeletal muscle atrophy	2022	10.1016/j.bcp.2022.114954	Review

Wang X	et al.	Impact of osteopenia and osteosarcopenia on the outcomes after surgery of hepatobiliary-pancreatic cancers,	2024	10.3389/fonc.2024.1403822	One or more keywords are missing
Wang Y	et al.	Interrelationships between sarcopenia, bone turnover markers and low bone mineral density in patients on hemodialysis,	2023	10.1080/0886022X.2023.2200846	One or more keywords are missing
Wang Y	et al.	lncRNA DLEU2 acts as a miR-181a sponge to regulate SEPP1 and inhibit skeletal muscle differentiation and regeneration	2020	10.18632/aging.104095	One or more keywords are missing
Watanabe H	et al.	Sarcopenia in chronic kidney disease: Factors, mechanisms, and therapeutic interventions	2019	10.1248/bpb.b19-00513	One or more keywords are missing
Weaver AA	et al.	Relationship of sarcopenia and osteosarcopenia with age, sex, injury severity, and fracture in seriously injured motor vehicle crash occupants,	2019	10.002/jbm4.10329	Conference Abstract
Weaver AA	et al.	Sarcopenia and osteosarcopenia in seriously injured motor vehicle crash occupants,	2019	10.1080/15389588.2019.1659620	One or more keywords are missing
Weigl L	et al.	Lost in translation: Regulation of skeletal muscle protein synthesis	2012	10.1016/j.coph.2012.02.017	Review
Weiwei	et al.	Methods and research progress in the construction of animal models of osteosarcopenia: a scoping review	2023	10.3389/fendo.2023.1228937	One or more keywords are missing
Wilhelmsen A	et al.	Recent advances and future avenues in understanding the role of adipose tissue cross talk in mediating skeletal muscle mass and function with ageing	2021	10.1007/s11357-021-00322-4	One or more keywords are missing

Williams J	et al.	Are microRNAs true sensors of ageing and cellular senescence?	2017	10.1016/j.arr.2016.11.008	Review
Witkowski J	et al.	Proteodynamics and aging of eukaryotic cells	2021	10.1016/j.mad.2021.111430	One or more keywords are missing
Wong R	et al.	High Prevalence of Osteosarcopenia amongst Hip Fracture Patients - Risk and Protective Factors?,	2024		Conference Abstract
Wu J	et al.	Proteomics Analysis Provides Insights into the Role of Lipid Metabolism in T2DM-Related Sarcopenia	2024	10.1021/acsomega.4c04668	One or more keywords are missing
Wu P	et al.	A case report of traditional chinese medicine in treating osteosarcopenia,	2016	10.1007/s00198-016-3530-x	Conference Abstract
Wu S	et al.	Recent advances in cell-based and cell-free therapeutic approaches for sarcopenia	2022	10.1096/fj.202200675R	Review
Wyce A	et al.	Research resource: The androgen receptor modulates expression of genes with critical roles in muscle development and function	2010	10.1210/me.2010-0138	One or more keywords are missing
Xhuti D	et al.	Circulating exosome-like vesicle and skeletal muscle microRNAs are altered with age and resistance training	2023	10.1113/JP282663	One or more keywords are missing
Xiang T	et al.	OSTEOSARCOPENIA AMONG PATIENTS UNDERGOING HEMODIALYSIS IS RELATED WITH MORTALITY,	2023	10.1093/ndt/gfad063c_4093	Conference Abstract
Xiang T	et al.	Sarcopenia and osteosarcopenia among patients undergoing hemodialysis,	2023	10.3389/fendo.2023.1181139	One or more keywords are missing

Xiao T	et al.	Prevalence and risk factors of osteosarcopenia in elderly patients with uncontrolled type 2 diabetes,	2025	10.1007/s12020-024-04001-0	One or more keywords are missing
Xie W	et al.	The Effect of MicroRNA-Mediated Exercise on Delaying Sarcopenia in Elderly Individuals	2020	10.1177/1559325820974543	Review
Xu J	et al.	Sarcopenia in liver cirrhosis: perspectives from epigenetics and microbiota	2023	10.3389/fmed.2023.1264205	Review
Xu Q	et al.	Colorectal Cancer Chemotherapy Drug Bevacizumab May Induce Muscle Atrophy Through CDKN1A and TIMP4	2022	10.3389/fonc.2022.897495	One or more keywords are missing
Xu R	et al.	Circulating miRNA-1-3p as Biomarker of Accelerated Sarcopenia in Patients Diagnosed with Chronic Heart Failure	2022	10.24875/RIC.22000151	One or more keywords are missing
Yam M	et al.	Sarcopenia in Distal Radius Fractures: A Scoping Review,	2022	10.14283/jfa.2022.6	Review
Yamada S	et al.	Association of Damaged Cardiovascular-Bone-Skeletal Muscle Axis with All-Cause Mortality and CKD Progression in Patients with Predialysis CKD: The Fukuoka Kidney Disease Registry (FKR) Study,	2024		Conference Abstract
Yamasaki T	et al.	Advances in Research on Brain Health and Dementia: Prevention and Early Detection of Cognitive Decline and Dementia	2024	10.3390/brainsci14040353	Editorial
Yanagaki M	et al.	Prognostic impact of osteosarcopenia in patients undergoing pancreatic resection for pancreatic ductal adenocarcinoma,	2024	10.1007/s00423-024-03315-x	One or more keywords are missing

Yanagaki M	et al.	The significance of osteosarcopenia as a predictor of the long-term outcomes in hepatocellular carcinoma after hepatic resection,	2023	10.1002/jhbp.1246	One or more keywords are missing
Yanai K	et al.	Micrornas in sarcopenia: A systematic review	2020	10.3389/fmed.2020.00180	Review
Yang J	et al.	Bibliometrics Analysis and Visualization of Sarcopenia Associated with Osteoporosis from 2000 to 2022,	2023	10.2147/JPR.S403648	One or more keywords are missing
Yang J	et al.	Bioinformatics and systems biology approaches to identify potential common pathogeneses for sarcopenia and osteoarthritis	2024	10.3389/fmed.2024.1380210	One or more keywords are missing
Yang YJ	et al.	An overview of the molecular mechanisms contributing to musculoskeletal disorders in chronic liver disease: Osteoporosis, sarcopenia, and osteoporotic sarcopenia,	2021	10.3390/ijms22052604	Review
Yanishi M	et al.	Factors Related to Osteosarcopenia in Kidney Transplant Recipients,	2018	10.1016/j.transproceed.2018.04.032	One or more keywords are missing
Yanishi M	et al.	Related factors of osteosarcopenia in kidney transplant recipients,	2018		Conference Abstract
Yannis D	et al.	Osteosarcopenia in chronic paraplegia,	2019		Conference Abstract
Yasuta S	et al.	Early postoperative decrease of skeletal muscle mass predicts recurrence and poor survival after surgical resection for perihilar cholangiocarcinoma	2022	10.1186/s12885-022-10453-2	One or more keywords are missing
Yedigaryan L	et al.	Shared and Divergent Epigenetic Mechanisms in Cachexia and Sarcopenia	2022	10.3390/cells11152293	Review

Yedigaryan L	et al.	Therapeutic implications of miRNAs for muscle-wasting conditions	2021	10.3390/cells10113035	Review
Yekta EB	et al.	Comparative study on muscle-tendon stiffness and balance impairment in postmenopausal women: a focus on osteosarcopenia and osteoporosis,	2024	10.1007/s40520-024-02888-3	One or more keywords are missing
Yin J	et al.	MicroRNA regulatory networks in the pathogenesis of sarcopenia	2020	10.1111/jcmm.15197	Review
Yin L	et al.	Skeletal muscle atrophy: From mechanisms to treatments	2021	10.1016/j.phrs.2021.105807	Review
Yin T	et al.	Identification of the circRNA-miRNA-mRNA regulatory network and its prognostic effect in colorectal cancer	2021	10.12998/wjcc.v9.i18.4520	One or more keywords are missing
Yoo Jl	et al.	Poor dietary protein intake in elderly population with sarcopenia and osteosarcopenia: A nationwide population-based study,	2020	10.11005/JBM.2020.27.4.301	One or more keywords are missing
Yoo Jl	et al.	The protective effect of IL12/23 p40 neutralizing antibody in sarcopenia induced by chronic inflammatory bowel disease,	2020	10.1016/j.bonr.2020.100521	Conference Abstract
Yoo Jl	et al.	Review of epidemiology, diagnosis, and treatment of osteosarcopenia in Korea,	2018	10.11005/jbm.2018.25.1.1	Review
Yoo Jl	et al.	Osteosarcopenia in Patients with Hip Fracture Is Related with High Mortality,	2018	10.3346/jkms.2018.33.e27	One or more keywords are missing
Yoshikoshi S	et al.	Prevalence of osteosarcopenia and its association with mortality and fractures among	2024	10.1007/s00774-024-01503-9	One or more keywords are missing

		patients undergoing hemodialysis,			
Yoshikoshi S	et al.	Osteosarcopenia Predicts Fractures and Mortality in Hemodialysis Patients,	2022		Conference Abstract
Yoshimura N	et al.	Profiles and risk factors for the occurrence of osteosarcopenia: The road study,	2018	10.1007/s00198-018-4465-1	Conference Abstract
Yoshimura N	et al.	Is osteoporosis a predictor for future sarcopenia or vice versa? Four-year observations between the second and third ROAD study surveys,	2017	10.1007/s00198-016-3823-0	One or more keywords are missing
Yu C	et al.	Research advances in crosstalk between muscle and bone in osteosarcopenia (Review),	2023	10.3892/etm.2023.11888	Review
Yu M	et al.	lncRNA GPRC5D-AS1 as a ceRNA inhibits skeletal muscle aging by regulating miR-520d-5p	2023	10.18632/aging.205279	One or more keywords are missing
Yu W	et al.	Differential expression profiles of miRNA in the serum of sarcopenic rats	2022	10.1016/j.bbrep.2022.101251	One or more keywords are missing
Zainul Azlan N	et al.	Chlorella vulgaris Modulates Genes and Muscle-Specific microRNAs Expression to Promote Myoblast Differentiation in Culture	2019	10.1155/2019/8394648	One or more keywords are missing
Zamboni M	et al.	The Role of Crosstalk between Adipose Cells and Myocytes in the Pathogenesis of Sarcopenic Obesity in the Elderly	2022	10.3390/cells11213361	Review
Zanker J	et al.	Osteosarcopenia: the Path Beyond Controversy,	2020	10.1007/s11914-020-00567-6	Review
Zanker J	et al.	Osteoporosis in Older Persons: Old and New Players,	2019	10.1111/jgs.15716	Review
Zemrani S	et al.	Dietary recommendations of the Moroccan Society of Rheumatology (SMR)	2024	10.1007/s11657-024-01461-9	Review

	for patients with ostÃ©osarcopenia,			
Zeng N et al.	Sestrins are differentially expressed with age in the skeletal muscle of men: A cross-sectional analysis	2018	10.1016/j.exger.2018.05.006	One or more keywords are missing
Zeng P et al.	MiR-378 attenuates muscle regeneration by delaying satellite cell activation and differentiation in mice	2016	10.1093/abbs/gmw077	One or more keywords are missing
Zhang H et al.	Exploring the effects of the enterokine FGF19 on bone in vivo and in vitro: A potential anti-osteosarcopenia agent?,	2023	10.1007/s40520-023-02442-7	Conference Abstract
Zhang J et al.	Protein Nutritional Support: The Classical and Potential New Mechanisms in the Prevention and Therapy of Sarcopenia	2020	10.1021/acs.jafc.0c00688	Review
Zhang M et al.	Alginate oligosaccharides relieve estrogen-deprived osteosarcopenia by affecting intestinal Th17 differentiation and systemic inflammation through the manipulation of bile acid metabolism,	2025	10.1016/j.ijbiomac.2025.139581	One or more keywords are missing
Zhang Y et al.	Neoadjuvant therapy increases the risk of metabolic disorders and osteosarcopenia in patients with early breast cancer,	2024	10.1093/jjco/hyae070	One or more keywords are missing
Zhang Y et al.	Transcriptomics, NF-?B pathway, and their potential spaceflight-related health consequences	2017	10.3390/ijms18061166	Review
Zhang Z et al.	A newly identified lncRNA MAR1 acts as a miR-487b sponge to promote skeletal muscle differentiation and regeneration	2018	10.1002/jcsm.12281	One or more keywords are missing

Zhao S	et al.	Associations of Body Mass Index and Percent Body Fat with Osteoporosis, Sarcopenia, and Osteosarcopenia: A Retrospective Study Based on Postmenopausal Women in China	2024	10.3390/healthcare13010028	One or more keywords are missing
Zheng Y	et al.	Role of miRNAs in skeletal muscle aging	2018	10.2147/CIA.S169202	One or more keywords are missing
Zhong J	et al.	Bioinformatics and system biology approach to identify potential common pathogenesis for COVID-19 infection and sarcopenia	2024	10.3389/fmed.2024.1378846	One or more keywords are missing
Zhu M	et al.	Luteolin: A promising multifunctional natural flavonoid for human diseases	2024	10.1002/ptr.8217	Review
Zhu X	et al.	Inflammation, epigenetics, and metabolism converge to cell senescence and ageing: the regulation and intervention	2021	10.1038/s41392-021-00646-9	Review
Zia A	et al.	Key miRNAs in Modulating Aging and Longevity: A Focus on Signaling Pathways and Cellular Targets	2022	10.2174/187446721466621091714154 1	Review
Zou Q	et al.	Research progress of exercise-mediated exosomes miRNA in the prevention and treatment of type 2 diabetes mellitus with sarcopenia	2023	10.3969/j.issn.1000-4718.2023.05.018	One or more keywords are missing
Zupan J	et al.	Age-related alterations and senescence of mesenchymal stromal cells: Implications for regenerative treatments of bones and joints	2021	10.1016/j.mad.2021.111539	One or more keywords are missing
		Abstracts from the Bone and Muscle Interactions: The Mechanical and Beyond Meeting	2019		Meeting
